# Distinct baseline immune characteristics associated with responses to conjugated and unconjugated pneumococcal polysaccharide vaccines in older adults

**DOI:** 10.1038/s41590-023-01717-5

**Published:** 2024-01-05

**Authors:** Sathyabaarathi Ravichandran, Fernando Erra-Diaz, Onur E. Karakaslar, Radu Marches, Lisa Kenyon-Pesce, Robert Rossi, Damien Chaussabel, Djamel Nehar-Belaid, David C. LaFon, Virginia Pascual, Karolina Palucka, Silke Paust, Moon H. Nahm, George A. Kuchel, Jacques Banchereau, Duygu Ucar

**Affiliations:** 1 The Jackson Laboratory for Genomic Medicine, Farmington, CT USA; 2 UConn Center on Aging, University of Connecticut, Farmington, CT USA; 3 Division of Pulmonary, Allergy and Critical Care Medicine, School of Medicine, University of Alabama at Birmingham, Birmingham, AL USA; 4 Drukier Institute for Children’s Health and Department of Pediatrics, Weill Cornell Medicine, New York, NY USA; 5 Department of Immunology and Microbiology, The Scripps Research Institute, La Jolla, CA USA; 6 Institute for Systems Genomics, University of Connecticut Health Center, Farmington, CT USA; 7 Department of Genetics and Genome Sciences, University of Connecticut Health Center, Farmington, CT USA; 8 Present Address: University of Buenos Aires, School of Medicine, Buenos Aires, Argentina; 9 Present Address: Leiden University Medical Center (LUMC), Leiden, the Netherlands; 10 Present Address: Immunoledge LLC, Montclair, NJ USA

**Keywords:** Conjugate vaccines, Conjugate vaccines

## Abstract

Pneumococcal infections cause serious illness and death among older adults. The capsular polysaccharide vaccine PPSV23 and conjugated alternative PCV13 can prevent these infections; yet, underlying immunological responses and baseline predictors remain unknown. We vaccinated 39 older adults (>60 years) with PPSV23 or PCV13 and observed comparable antibody responses (day 28) and plasmablast transcriptional responses (day 10); however, the baseline predictors were distinct. Analyses of baseline flow cytometry and bulk and single-cell RNA-sequencing data revealed a baseline phenotype specifically associated with weaker PCV13 responses, which was characterized by increased expression of cytotoxicity-associated genes, increased frequencies of CD16^+^ natural killer cells and interleukin-17-producing helper T cells and a decreased frequency of type 1 helper T cells. Men displayed this phenotype more robustly and mounted weaker PCV13 responses than women. Baseline expression levels of a distinct gene set predicted PPSV23 responses. This pneumococcal precision vaccinology study in older adults uncovered distinct baseline predictors that might transform vaccination strategies and initiate novel interventions.

## Abstract (web summary)

Ravichandran et al. performed a systems immunology study to profile the responses to pneumococcal vaccines in older adults. They identified distinct baseline features that could capture responses to Prevnar and Pneumovax and sex-biased differences in Prevnar responses.

## Main

*Streptococcus pneumoniae* (pneumococcus) infections lead to life-threatening diseases and are responsible for thousands of hospitalizations and deaths annually in the United States^1^. Infants and older adults have the highest rates of invasive pneumococcal disease, with an ~10% mortality rate particularly affecting older adults according to reports from the Centers for Disease Control and Prevention reports. Two types of pneumococcal vaccines were approved by the Food and Drug Administration (FDA) for use in this population: the capsular polysaccharide vaccine Pneumovax® (PPSV23) and protein-polysaccharide conjugate vaccines (for example, Prevnar® (PCV13)). PPSV23 contains capsular polysaccharide antigens for 23 serotypes that elicit antibody responses in the absence of CD4^+^ T cell collaboration^2^. Responses to unconjugated polysaccharides are predominantly mediated by the activation of marginal zone (MZ) B cells, which possess hypermutated immunoglobulin genes and express high levels of complement receptor 2 (CD21)^3,4^. MZ B cells are not fully mature in newborns and children, resulting in limited success of unconjugated polysaccharide vaccines^3,5,6^. Furthermore, older individuals show a marked decrease in frequencies of total memory and MZ B cells, which might contribute to the age-related decline of responses^7^. After activation, MZ B cells terminally differentiate into IgM-, IgG- and IgA-secreting plasma cells^8^. Although MZ B cell activation and IgM production are considered to be T cell independent, IgG and IgA class switching might rely on the presence of non-cognate T cells that secrete IL-21 and/or trigger the CD40/CD40L signaling axis^8^. Conjugated vaccines have been developed (for example, PCV13) to enhance immunogenicity. PCV13 is composed of polysaccharides for the most prevalent 13 serotypes conjugated to a nontoxic variant of diphtheria toxin CRM197. PCV13 induces T cell-dependent activation and expansion of B2 follicular cells, promoting the generation of memory B cells and differentiation of long-lived plasma cells^9–11^.

Conjugated pneumococcal vaccines are effective among children and young adults^1^ and in preventing invasive disease among older adults^12^, yet their efficacy declines with age^13–15^. PCV13 is 90% effective in infants and children, which decreases to 72.8% among older adults. Similarly, the efficacy of PPSV23 is 54% for adults^16^. In 2021, other conjugated vaccines were approved, including PCV15 and PCV20, to extend the range of serotypes covered and to simplify pneumococcal immunizations^17^. In the United States, the current recommendation for older adults is to receive either PCV20 or PCV15 followed by PPSV23 1 year later.

Peripheral blood leukocyte gene expression studies revealed transcriptional differences in responses to PPSV23 and influenza vaccines among healthy adults^18,19^. However, these studies did not include conjugated pneumococcal vaccines and excluded older adults partially due to the complexity of evaluating their responses. To address these gaps, we recruited 39 healthy pneumococcal vaccine-naive adults ≥60 years old who received PCV13 or PPSV23. We collected blood from this cohort longitudinally and studied their baseline immune phenotypes and immune responses following vaccination by using multiple assays. Our systems vaccinology approach uncovered distinct baseline gene expression modules associated with PCV13 and PPSV23 responses, which can pave the way toward more precise administration of these vaccines.

## Results

### Serological responses of older adults to PCV13 and PPSV23

**Fig. 1:**
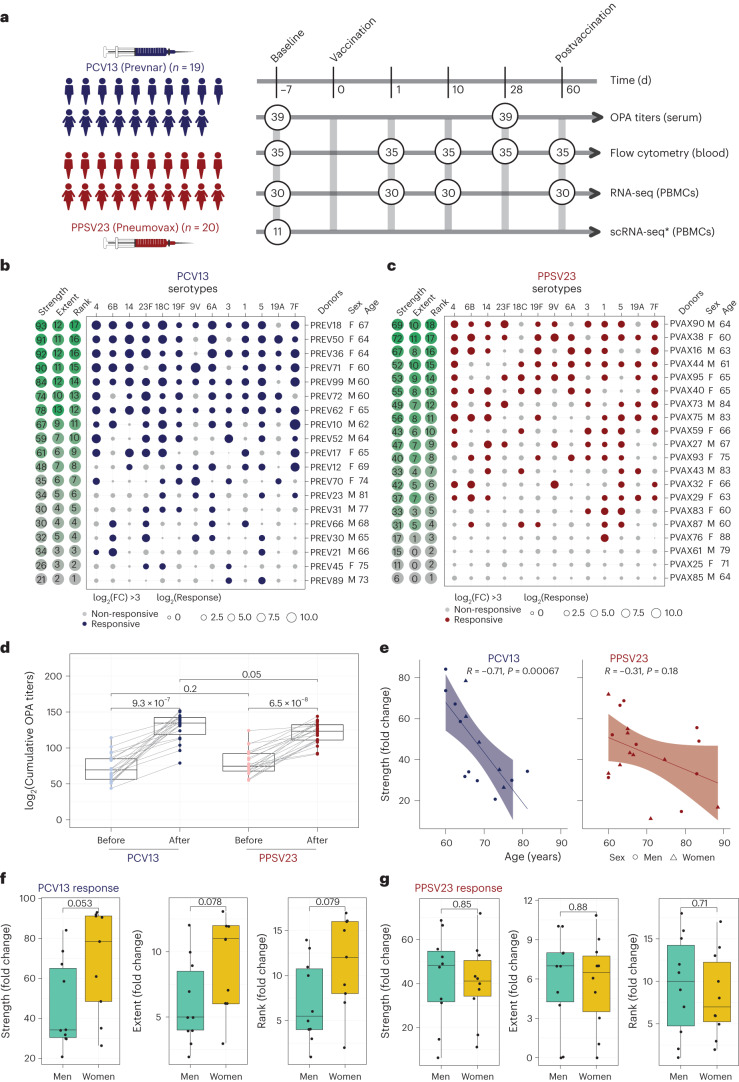
Functional antibody response to PCV13 and PPSV23 in older adults. **a**, Schematic representation of the study design. Nine women and ten men received the PCV13 vaccine, and ten women and ten men received PPSV23. OPA titers for the 13 serotypes were assessed from serum samples obtained 7 d before vaccination (baseline) and 28 d after vaccination for both vaccines. Anticoagulated blood samples were used for flow cytometric analysis of whole-blood cell populations. PBMCs were isolated for bulk RNA-seq. Prevaccination PBMCs from four women and seven men who received PCV13 were isolated for scRNA-seq. The numbers in circles represent the total numbers of biologically independent samples processed for the indicated assays at the indicated times. Figure created with BioRender.com. The star indicates scRNA-seq data generated exclusively for the PCV13 baseline samples. **b**, Bubble plot of fold change (FC) in antibody titers for individual serotypes in response to PCV13 (*n* = 19); M, male; F, female. **c**, Bubble plot of fold change in antibody titers for individual serotypes in response to PPSV23 (*n* = 20). Dot size represents the fold change value, and color indicates a significant response (log_2_(fold change) is >3), with blue for PCV13 and red for PPSV23. Donors are ordered from top to bottom according to the vaccine response rank. On the left, the strength (log_2_(sum fold change)), extent of response (number of serotypes out of 13 to which an individual mounted a significant response) and rank are presented. **d**, Prevaccination and postvaccination cumulative OPA titers (expressed as sum log_2_) in response to PCV13 (*n* = 19) and PPSV23 (*n* = 20). **e**, Correlation analysis between the cumulative fold change (sum log_2_(fold change)) and age (in years). **f**,**g**, Sex-specific differences in strength, extent and rank in donors who received PCV13 (*n* = 19; **f**) and PPSV23 (*n* = 20; **g**). A Wilcoxon matched-pairs signed-rank test (two sided) was used in **d** to compare titers before and after vaccination with PCV13 or PPSV23. Box plots display the median and interquartile range (IQR; 25–75%), with whiskers representing the upper and lower quartiles ±1.5× IQR. A Wilcoxon rank-sum test (two sided) was used to compare strength, extent and rank between men and women vaccinated with PCV13 and PPSV23. The Pearson correlation metric was used to perform correlation analyses between strength and age (**e**), and *P* values were computed using two-sided *t*-tests; *n* represents the number of biological replicates. Source data

During the 2017–2018 seasons, 39 older adults (≥60 years of age with no history of pneumonia disease or vaccination) were recruited and randomized to receive either a dose of PCV13 (ten men and nine women) or PPSV23 (ten men and ten women). Vaccines were administered from May to early fall to avoid overlap with the influenza vaccine season. Donors in each arm of the study and the men and women in each arm were comparable in terms of age, body mass index (BMI), cytomegalovirus (CMV) seropositivity and frailty index (Fig. 1a). Donors were not on any confounding treatments, but some were on medications for common chronic diseases (Supplementary Table 1a,b).

Blood samples were collected 7 d before vaccination (baseline) and at 1, 10, 28 and 60 d after vaccination. Previously, we showed that transcriptional innate responses to PPSV23 peaked at day 1, whereas adaptive responses peaked at day 7 and remained high until day 14 in adults^19^. Hence, days 1 and 10 were chosen to capture the innate and adaptive responses while also ensuring participant safety and adherence to the institutional review board recommendations regarding the maximum volume of blood one can collect from older adults (550 ml over an 8-week period or 69 ml per week). We generated longitudinal flow cytometry and RNA-sequencing (RNA-seq) data to study immune responses to these two vaccines (Fig. 1a). Pneumococcal vaccine responses can be assessed by measuring antibody levels using enzyme-linked immunosorbent assays (ELISAs) or antibody function using opsonophagocytic activity (OPA). OPA measures the ability of antibodies to effectively opsonize bacteria and is ideal for quantifying responses because it mimics the in vivo mechanisms of antibody protection^20^ and is hence considered state of the art for FDA approvals^21^. Unlike children, many older adults already possess high ELISA antibody levels before vaccination, making functional OPA assays more attractive in this population^20^. Therefore, we used OPA to quantify antibody responses from serum (at baseline and 28 d after vaccination). Because OPA assays require a substantial volume of serum, we prioritized the 13 most prevalent serotypes: the 12 shared serotypes and serotype 6A from PCV13 (Supplementary Table 2)^22^. Quantifying the common serotypes enabled a side-by-side comparison of these two vaccines.

**Extended Data Fig. 1:**
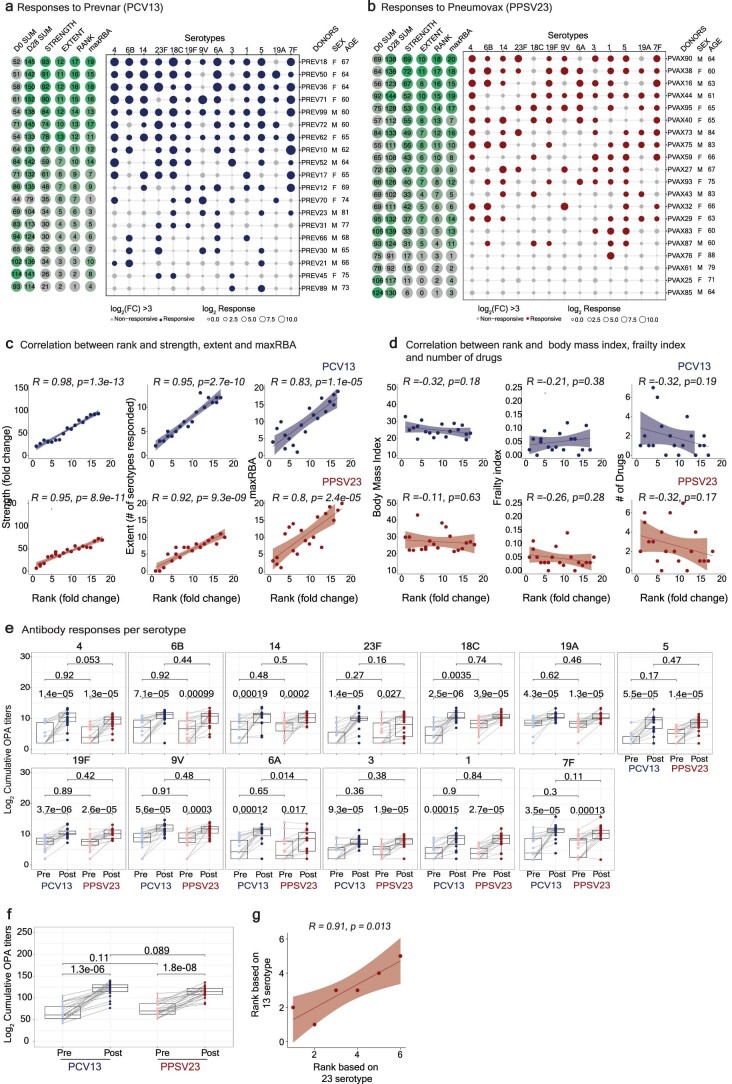
Functional antibody response to PCV13 and PPSV23. Bubble plot showing the fold change in the antibody titers for individual serotypes in response to PCV13 (n = 19) **a)** and PPSV23 (n = 20) **b)**. The size of the dots represents the magnitude of the fold change (FC) value, and the color indicates significant response (Log_2_ FC is > 3): blue for PCV13 and red for PPSV23. Donors are ordered from top to bottom according to the vaccine response Rank. On the left side, D0 SUM (sum of pre-vaccination OPA titers to 13 serotypes), D35 SUM (sum of 28 days post-vaccination OPA titers to 13 serotypes), the Strength, the Extent, the Rank and the maxRBA rank (sum of baseline adjusted fold changes to 13 serotypes) for each donor is displayed. **c)** Correlation between the Rank and the Strength, the Extent, the maxRBA for PCV13 (n = 19; top panel) and PPSV23 (n = 20; bottom panel). **d)** Correlation between the Rank and body mass index (BMI), frailty index (FI) and number of concomitant drugs. **e)** OPA titers (Log_2_) for individual serotypes showing the connecting lines between Pre and post-vaccination. **f)** Pre-vaccination and post-vaccination cumulative OPA titers (expressed as sum Log_2_) excluding serotype 6A. **g)** Correlation of Rank determined from all 23 serotypes and a subset of 13 serotypes present in the PCV13 in an independent cohort. The Pearson correlation metric was used to perform correlation analysis (c, d, and g) and p-value was generated using two-sided t-test. Boxplots display the median and IQR (25–75%), with whiskers representing the upper- and lower-quartile ±1.5 × IQR. The Wilcoxon matched-pairs signed-rank test (two-sided) was used in to compare the difference in mean between pre- and post-vaccination OPA titers for 13 serotypes (e, f) in PCV13 and PPSV23. The ‘n’ represents number of biological replicates. Source data

We developed three multivariate measures to quantify vaccine responses: (1) strength of the response (the sum of fold change values between baseline and day 28 for all serotypes), (2) extent (the number of serotypes (of 13) to which the donor elicits significant (that is, an OPA titer of ≥8) responses) and (3) rank (the rank of an individual’s vaccine responsiveness in the cohort based on aggregate responses for all serotypes, where higher ranks represent stronger responses; Fig. 1b,c, Extended Data Fig. 1a,b and Supplementary Table 2d,h). To assess whether quantifying PPSV23 responses using 13 serotypes fully captures responsiveness to this vaccine, we reanalyzed OPA titers for all 23 serotypes from another study^11^, which revealed a strong and significant correlation between ranking using 13 serotypes and ranking using all serotypes (*R* = 0.91, *P* = 0.013; Extended Data Fig. 1g). Strength and extent of responsiveness were highly correlated with the rank, while capturing different aspects of vaccine responses (Extended Data Fig. 1c). BMI, frailty index and number of concomitant medications were not associated with responsiveness to either vaccine (Extended Data Fig. 1d and Supplementary Table 1a,b).

**Extended Data Fig. 2:**
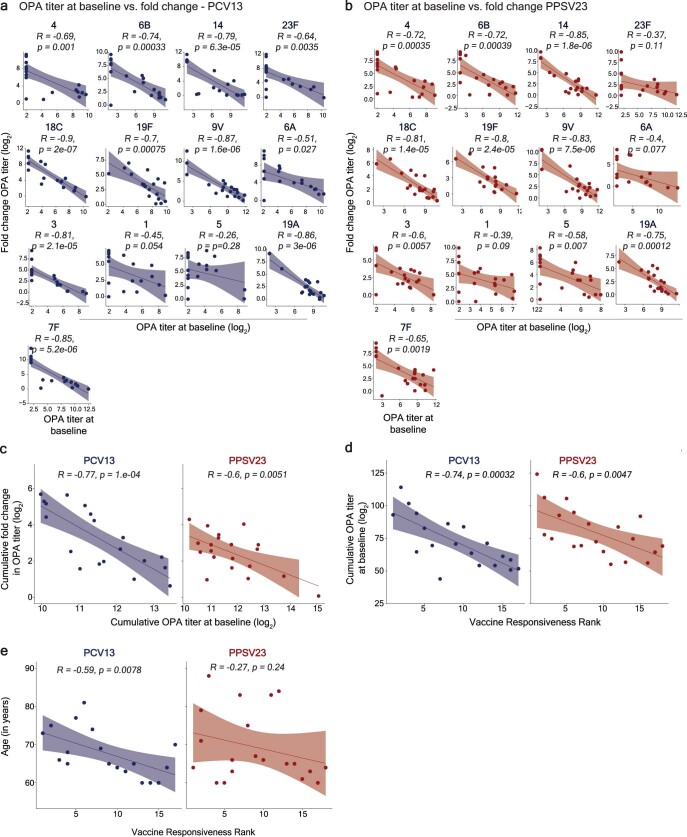
Baseline OPA titers inversely correlate with fold change in OPA titers and vaccine responsiveness ranks. Correlation between baseline OPA titers and their post-vaccination fold change for individual serotypes for PCV13 (n = 19) **a)** and PPSV23 (n = 20) **b). c)** Association between cumulative baseline OPA titer and fold change for both PCV13 (n = 19) and PPSV23 (n = 20). Both baseline and fold change in OPA titers are expressed on a log_2_ scale (a, b, c). **d)** Correlation between cumulative baseline OPA titer and vaccine responsiveness rank for both vaccines. **e)** Correlation between Rank and age (in years) for PCV13 and PPSV23. The Pearson correlation metric was used to perform correlation analysis and p-value was generated using two-sided t-test. The ‘n’ represents number of biological replicates. Source data

After vaccination, OPA titer levels significantly increased for both vaccines cumulatively (Fig. 1d) and for each serotype (Extended Data Fig. 1e). Stronger PCV13 responses were detected for serotype 4 and the PCV13-specific serotype 6A (Extended Data Fig. 1e). Although PPSV23 does not contain serotype 6A, it induced increased 6A responses potentially due to cross-reactivity with serotype 6B. Baseline OPA titers were comparable between the two cohorts (*P* = 0.2); however, individuals vaccinated with PCV13 had stronger responses (Fig. 1d; *P* = 0.05) even after excluding serotype 6A (*P* = 0.089; Extended Data Fig. 1f). Level of responsiveness varied from one individual to another. In the group treated with PCV13, top responder PREV18 (female, 67 years old) responded significantly to all serotypes except for 19A, whereas the bottom responder PREV89 (male, 73 years old) elicited significant responses to only two serotypes (Fig. 1b). In PPSV23, top responder PVAX90 (male, 64 years old) responded to ten serotypes, whereas the bottom responder PVAX25 (female, 71 years old) did not respond significantly to any of the tested serotypes (Fig. 1c). Our ranking strategy captured this heterogeneity at the donor level. There was a significant inverse correlation between baseline OPA titer levels and fold change in titers (Extended Data Fig. 2a–c), in alignment with previous studies^23^. There was a significant negative correlation between cumulative OPA titers at baseline and vaccine responsiveness rank (Extended Data Fig. 2d). Given this association, we calculated the maximum residual after baseline adjustment (maxRBA) values (Extended Data Fig. 1a,b), which correct for baseline titer levels by modeling fold increase in titer levels as an exponential function of the baseline titer levels. maxRBA and rank correlated significantly for both vaccines (Extended Data Fig. 1c).

Donor age was negatively associated with both strength (Fig. 1e; *R* = −0.71 and *P* = 0.00067 for PCV13, *R* = −0.31 and *P* = 0.18 for PPSV23) and rank (Extended Data Fig. 2d). Although male and female donors were comparable in terms of age, frailty and BMI (Supplementary Table 1b), women mounted stronger responses to the PCV13 vaccine (*P* = 0.053 for strength, *P* = 0.079 for rank; Fig. 1f), which was not observed for PPSV23 (Fig. 1g). Four of five top responders were women and four of five bottom responders were men in the group treated with PCV13. Overall, these data showed that both pneumococcal vaccines induced strong antibody responses; yet, at the individual level, the degree of responsiveness was variable. For PCV13, donor age significantly contributed to this variation, and there was a trend for stronger responsiveness for women. Interestingly, neither age nor sex was significantly associated with PPSV23 responsiveness.

### PCV13 and PPSV23 induce similar transcriptional responses

**Fig. 2:**
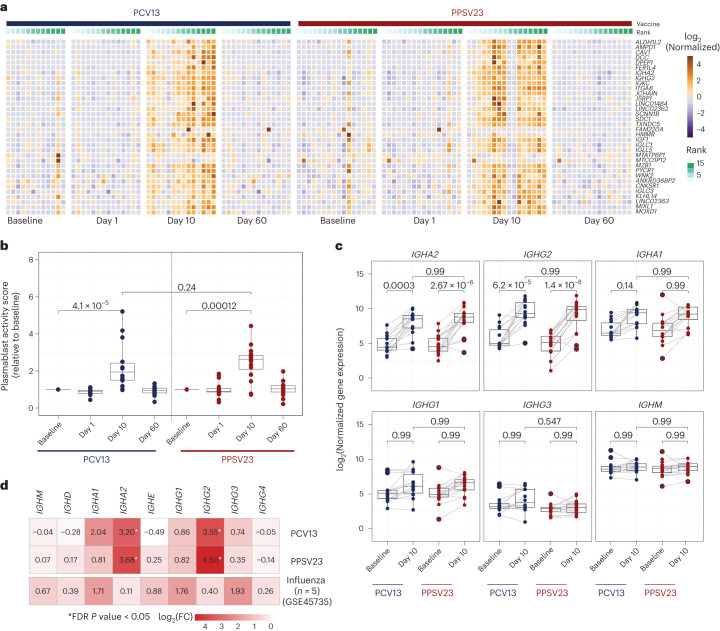
Plasmablast response elicited after vaccination at day 10 in PBMCs. **a**, Heat map of differentially expressed genes between day 10 and baseline assessed using normalized gene expression values. **b**, Box plot of plasmablast activity scores at baseline and days 1, 10 and 60 in response to PCV13 (*n* = 14) and PPSV23 (*n* = 16), calculated using a published gene set (M4.11) and scaled with reference to baseline. **c**, Box plots showing normalized expression of genes encoding the constant region of the immunoglobulin heavy chain structure in response to PCV13 (*n* = 14) and PPSV23 (*n* = 16). **d**, Heat map showing differential expression of genes encoding the constant region of the immunoglobulin heavy chain structure at day 10 in response to PCV13 (*n* = 14) and PPSV23 (*n* = 16) and at day 7 in response to Fluzone (GSE45735 (influenza vaccine); *n* = 5). Genes with a >1.5-fold difference and FDR-corrected *P* value of <0.05 are marked with an asterisk (*). A Wilcoxon rank-sum test (two sided) was used to compare plasma cell activity scores between baseline and days 1, 10 and 60 (**b**). Box plots display the median and IQR (25–75%), with whiskers representing the upper and lower quartiles ±1.5× IQR. A Wilcoxon matched-pairs signed-rank test (two sided) was used to compare the expression of immunoglobulin genes at baseline and day 10 for PCV13 and PPSV23. FDR-corrected *P* values are shown in **c**; *n* represents the number of biological replicates. Source data

**Extended Data Fig. 3:**
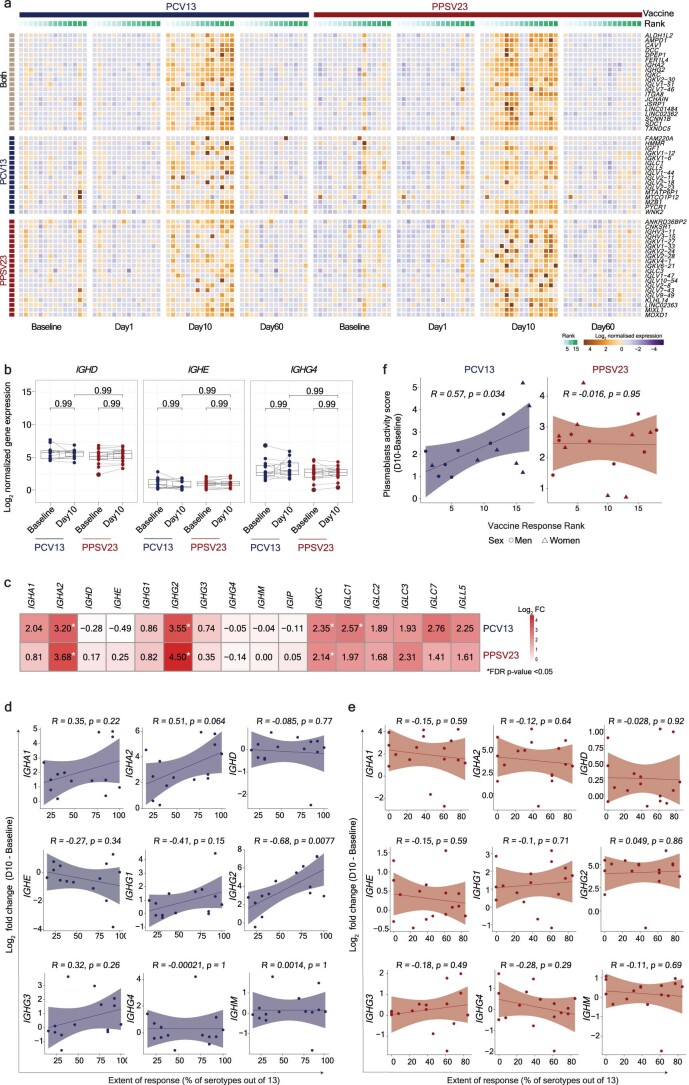
Differential transcriptional changes in response to PCV13 and PPSV23 at day 10. **a**) Heatmap showing differentially expressed genes between Day 10 and baseline, using the normalized gene expression. Differentially expressed genes that are common for both vaccines or show differential expression only in PCV13 or PPSV23 are grouped separately. **b**) Boxplots of the normalized expression of genes coding for the constant region of immunoglobulin heavy chain structure. **c**) Heatmap of differential expressed genes coding for the constant region of immunoglobulin heavy chain structure at Day 10 in response to PCV13 and PPSV23. Genes with a FC >1.5-fold difference and a FDR p-value <0.05 are marked with stars. **d)** Correlation analysis between the fold difference in immunoglobulin genes (gene expression at Day 10 - baseline) and the extent of the response (% of serotypes out of 13) for PCV13. **e)** Correlation analysis between the fold difference in immunoglobulin genes (gene expression at Day 10 - baseline) and the extent of the response (% of serotypes out of 13) for PPV23. **f**) Correlation between the plasmablasts activity scores (Day 10 vs. baseline) and vaccine responsiveness Rank. The Pearson correlation metric was used to perform correlation analysis (a, c, and d). The Wilcoxon matched-pairs signed-rank test (two-sided) was used to compare the expression of immunoglobulin genes at baseline and Day 10 for PCV13 and PPSV23 (c). The ‘n’ represents number of biological replicates. Source data

We generated longitudinal bulk RNA-seq data at baseline and days 1, 10 and 60 from peripheral blood mononuclear cells (PBMCs) from 31 donors (15 PCV13 and 16 PPSV23), including strong responders (SRs) and weak responders (WRs) for each vaccine while also balancing female and male samples. Samples from 30 donors that passed quality control were used in differential analyses. Gene expression profiles after vaccination were compared to those at baseline (day −7; Supplementary Table 3). There were no significant transcriptional changes for days 1 or 60. However, at day 10, 50 and 41 genes were differentially expressed compared to baseline (false discovery rate (FDR) < 0.05), respectively, for PCV13 and PPSV23 (Extended Data Fig. 3a). Upregulated genes after vaccination included immunoglobulin heavy chain genes *IGHG2* and *IGHA2* and plasmablast-associated genes *JCHAIN* and *MZB1* (Fig. 2a). The activity of these genes peaked at day 10 and returned to baseline levels by day 60 (Fig. 2b). A cumulative plasmablast activity score using plasma cell-specific genes^19^ (*n* = 33 genes) confirmed a significant and comparable increase in the expression of plasmablast-associated genes at day 10 for both vaccines that returned to baseline levels at day 60 (Fig. 2b). *IGHG2*, encoding the IgG2 isotype, was among the most upregulated genes for both vaccines (Fig. 2c). *IGHA2*, encoding the IgA2 isotype, was also upregulated for both vaccines (Fig. 2c and Extended Data Fig. 3b), in alignment with the serology data^24^.

Pneumococcal vaccines upregulated distinct immunoglobulin genes compared to the influenza vaccine. Reanalysis of prior PBMC RNA-seq data on influenza vaccine responses in younger adults showed upregulated expression of *IGHG1* and *IGHG3* at day 7, whereas pneumococcal vaccines upregulated the expression of *IGHA2* and *IGHG2* (Fig. 2d and Extended Data Fig. 3c). Transcriptional activation of plasmablast-associated genes significantly correlated with PCV13 responsiveness (*R* = 0.57, *P* = 0.034), mainly driven by *IGHG2* expression (Extended Data Fig. 3d,f), which was not the case for PPSV23 (Extended Data Fig. 3e,f). Together, transcriptional profiling before and after vaccination revealed that both pneumococcal vaccines induced significant and similar plasmablast responses 10 d after vaccination, including increased transcription of genes encoding IgG2 and IgA2 antibody isotypes, contrasting with those (IgG1, IgG3 and IgA1) induced by the influenza vaccine.

### Baseline frequencies of T_H_1 and T_H_17 cells are linked to PCV13 responses

**Fig. 3:**
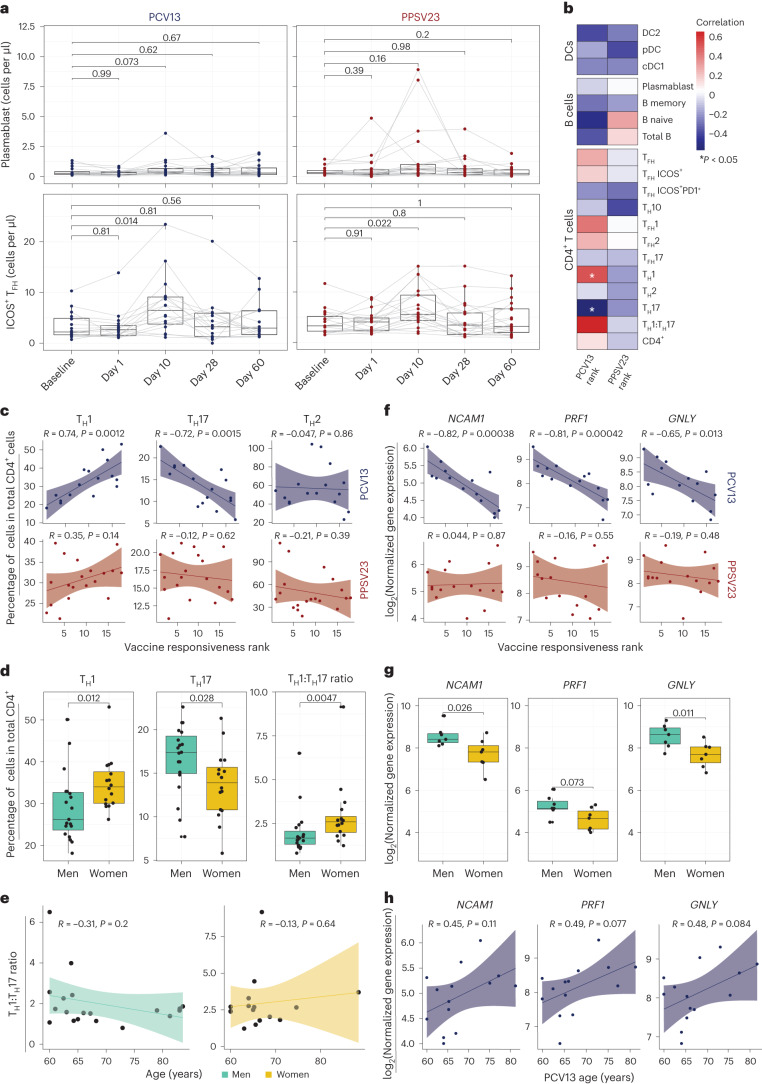
Baseline T_H_1:T_H_17 cell ratio and cytotoxic gene expression are predictive of PCV13 vaccine responsiveness rank. **a**, Longitudinal analysis of the absolute numbers of plasmablasts (cells per μl; top) and ICOS^+^ T_FH_ cells (cells per μl; bottom) among the memory CD4^+^ T cell population in response to PCV13 (*n* = 16) and PPSV23 (*n* = 19). **b**, Correlation analysis between the absolute number of different cell types (DC, B cell and CD4^+^ T cell subsets) analyzed in whole blood and ranks. **c**, Correlation analysis between ranks and frequencies of T_H_1, T_H_17 and T_H_2 cells evaluated at baseline. **d**, Sex differences in the frequencies of T_H_1 and T_H_17 cells and T_H_1:T_H_17 cell ratio at baseline (*n* = 16 for PCV13; *n* = 19 for PPSV23). T_H_1 and T_H_17 cell frequencies were calculated relative to the total CD4^+^ T cell count. **e**, Association between T_H_1:T_H_17 cell ratio and age among men (green) and women (dark yellow) in response to PCV13 and PPSV23. **f**, Correlation analysis between baseline expression of cytotoxic genes (*NCAM1*, *GNLY* and *PRF1*) and PCV13 vaccine responsiveness rank (top; *n* = 14) and PPSV23 vaccine responsiveness rank (bottom; *n* = 16). **g**, Sex differences in the expression of *NCAM1*, *PRF1* and *GNLY* at baseline (*n* = 14 for PCV13; *n* = 16 for PPSV23). **h**, Association between *NCAM1*, *PRF1* and *GNLY* expression and age (*n* = 14 for PCV13; *n* = 16 for PPSV23). Box plots display the median and IQR (25–75%), with whiskers representing the upper and lower quartiles ±1.5× IQR. A Wilcoxon matched-pairs signed-rank test (two sided) was used to compare the absolute numbers of plasmablasts and ICOS^+^ T_FH_ cells longitudinally (**a**). Correlation analyses were performed using the Pearson correlation metric (**b**, **c**, **e**, **f** and **h**), and *P* values were computed using two-sided *t*-tests; *n* represents the number of biological replicates. Source data

**Extended Data Fig. 4:**
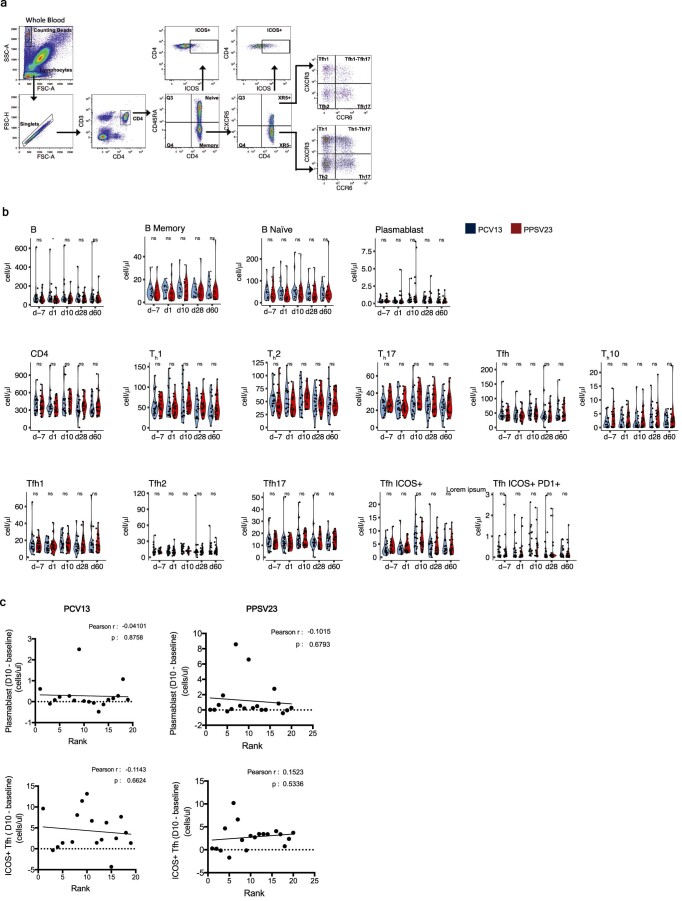
No association between changes in plasmablasts and ICOS^+^ Tfh cells at Day 10 and responses to both vaccines. **a**) Representative plots showing the gating strategy for the characterization by flow cytometry of the different memory CD4+ T cell populations. **b**) Comparative analysis of the absolute number of the different cell types between PCV13 (n = 16) and PPSV23 (n = 19) cohorts over time. **c**) Correlation between fold difference in plasmablasts cell numbers (d10 - baseline) and vaccine responsiveness Rank (top panel). Correlation between fold difference in ICOS+ Tfh cell numbers (d10 - baseline) and vaccine responsiveness Rank (bottom panel). The Wilcoxon Rank sum test was used to compare the absolute numbers of different cell types: * (p < 0.05), ** (p < 0.01), ***(p < 0.001), **** (p < 0.0001) (b). The Pearson correlation metric was used to perform correlation analysis (a, b, and c), and the p-value was computed using two-sided t test (c). The ‘n’ represents number of biological replicates. Source data

We performed a longitudinal flow cytometry analysis of different cell populations in freshly isolated PBMCs at baseline (day −7) and after vaccination at days 1, 10, 28 and 60 to study B cell (naive, memory and plasmablast cells), CD4^+^ T cell (naive, type 1 helper T (T_H_1), type 2 helper T (T_H_2), interleukin-17 (IL-17)-producing helper T (T_H_17), IL-10-producing helper T (T_H_10), follicular helper T (T_FH_), type 1 T_FH_ (T_FH_1), type 2 T_FH_ (T_FH_2) and IL-17-producing T_FH_ (T_FH_17) cells) and dendritic cell (DC) subsets (type 2 classical DCs (cDC2s), type 1 cDCs (cDC1s) and plasmacytoid DCs (pDCs); Supplementary Table 4a and Extended Data Fig. 4a,b). Despite the robust plasmablast transcriptional signature, their absolute numbers were only marginally increased at day 10 for both vaccines (Fig. 3a, top). There were no significant changes in other B cell subsets after vaccination or between the two cohorts (Extended Data Fig. 4b). Among the studied T cell subsets, only ICOS^+^ T_FH_ cell numbers significantly increased at day 10 for both vaccines (Fig. 3a, bottom, and Extended Data Fig. 4b). There were no significant changes in other T cell or DC subsets after vaccination (Extended Data Fig. 4b). Cell numbers were similar between the two cohorts for all cell types both at baseline and after vaccination (Extended Data Fig. 4b). Therefore, the most significant changes included an increase in plasmablasts and ICOS^+^ T_FH_ cells at day 10, which was comparable between the two vaccines. However, unlike our previous observations for influenza vaccines^25^, changes in these cell types did not correlate with vaccine responsiveness (Extended Data Fig. 4c).

**Extended Data Fig. 5:**
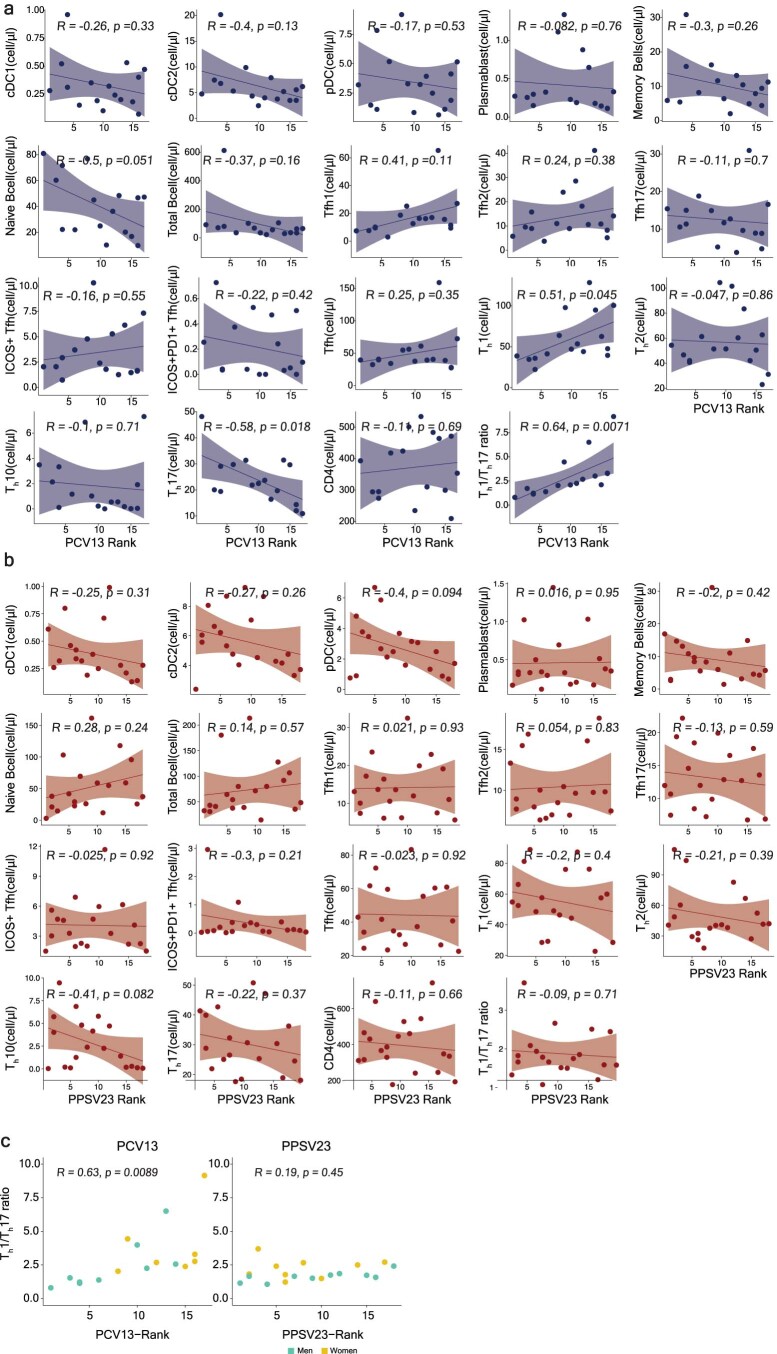
Higher T_h_1/T_h_17 ratio mount stronger responses to PCV13. **a**) Correlation analysis between PCV13 Rank and the absolute numbers (cells/ul) of the different cell populations at baseline (d-7) (n = 16). **b**) Correlation analysis between PPSV23 Rank and the absolute numbers (cells/ul) of the different cell populations at baseline (d-7) (n = 19). **c)** Correlation analysis of T_h_1/T_h_17 ratio and vaccine responsiveness Ranks of PCV13 (n = 16) and PPSV23 (n = 19), respectively. The Pearson correlation metric was used to perform correlation analysis (a, b, and c), and the p-value was computed using two-sided t test. The ‘n’ represents number of biological replicates. Source data

To uncover baseline cellular predictors, we performed correlation analyses between the prevaccination absolute cell numbers for B cell, T cell and DC subsets and vaccine responsiveness (rank) for each vaccine. None of the profiled cell types were significantly associated with PPSV23 (Fig. 3b). By contrast, T_H_1 cell frequency (*R* = 0.74, *P* = 0.0012) was positively associated with PCV13 responses, while proinflammatory T_H_17 cell frequency (*R* = −0.72, *P* = 0.0015) was negatively associated with PCV13 responses (Fig. 3b,c; see Extended Data Fig. 5a,b for cell numbers). The ratio of T_H_1 cell frequency to T_H_17 cell frequency was the strongest predictor of PCV13 responses (*R* = 0.63, *P* = 0.0089) but not PPSV23 responses (Fig. 3b and Extended Data Fig. 5c). There was no significant difference in T_FH_ frequency between SRs and WRs, although T_FH_1 cells showed a positive association with PCV13 responsiveness, and T_FH_17 cells showed a negative association with PCV13 responsiveness (Fig. 3b). We observed sex differences in T_H_1 and T_H_17 cell frequencies; women had higher T_H_1 (*P* = 0.012) and lower T_H_17 cell frequencies (*P* = 0.028) than men (Fig. 3d and Extended Data Fig. 5c). Women with higher T_H_1:T_H_17 cell ratios mounted stronger responses to PCV13 (Extended Data Fig. 5c). These two cell types and their ratios were not significantly associated with age (Fig. 3e). Together, these data established that individuals with higher frequencies of T_H_1 cells and lower frequencies of T_H_17 cells before vaccination mount stronger responses to the conjugated PCV13 vaccine.

### Baseline CYTOX level is negatively associated with PCV13 responses

**Extended Data Fig. 6:**
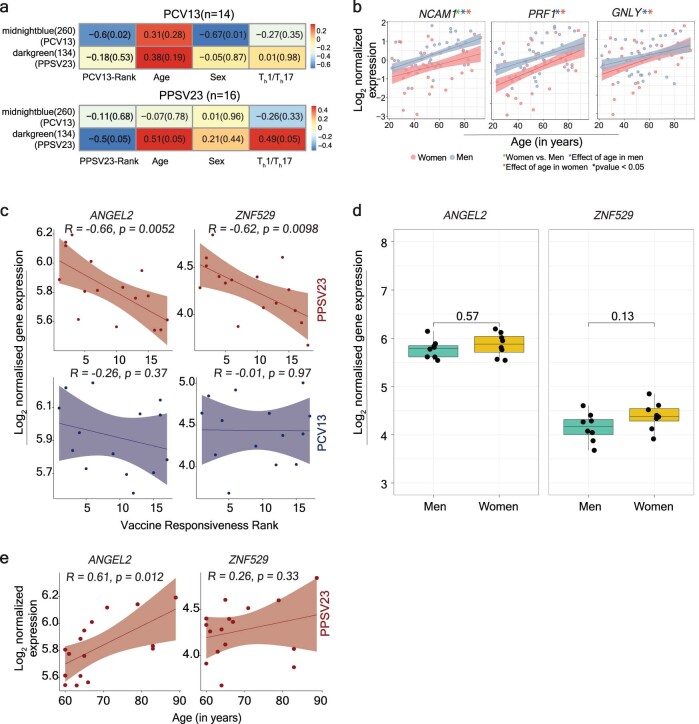
Baseline expression of ‘darkgreen module’ genes negatively associated with PPSV23 responses. **a**) Heatmap showing the modules correlated with PCV13 Rank, PPSV23 Rank, sex, age and T_h_1/T_h_17 ratio. **b**) Correlation between the expression of cytotoxic genes and age (in years) in an independent dataset. **c**) Correlation analysis between top correlates of darkgreen module (*ANGEL2*, and *ZNF529)* and PPSV23 Rank (n = 16). Correlation analysis between top correlates of darkgreen module (*ANGEL2*, and *ZNF529)* and PCV13 Rank (n = 14). **d)** Boxplot showing the expression of top correlates of darkgreen module (*ANGEL2*, and *ZNF529*) in men and women (n = 16). **e)** Association between *ANGEL2*, and *ZNF529* expression with age (in years). Boxplots display the median and IQR (25–75%), with whiskers representing the upper- and lower-quartile ±1.5× IQR. The Wilcoxon Rank sum test (two-sided) was used to compare the expression of genes in men and women (e). Correlation analysis was performed using the Pearson correlation metric (b,c,e), and the p-value was computed using two-sided t test. The ‘n’ represents number of biological replicates. Source data

To identify baseline transcriptional signatures associated with vaccine responsiveness, we conducted weighted gene coexpression network analysis (WGCNA) on PBMC RNA-seq data, which yielded 33 gene modules. We associated these modules with vaccine responsiveness, sex, age and T_H_1:T_H_17 cell ratio. Of 33 modules, only 1 (midnight blue, 260 genes) was significantly (*P* = 0.02) and specifically associated with PCV13 responses (Extended Data Fig. 6a and Supplementary Table 5a). Genes in this module were enriched in the ‘natural killer cell mediated cell cytotoxicity’ KEGG pathway (*P* = 0.000003639) and included *NCAM1*, the marker gene for natural killer (NK) cells, and several transcripts (*GNLY*, *PRF1* and *GZMB*) encoding cytotoxic functions^26^ (Supplementary Table 6a). This cytotoxicity-associated gene module is hereafter referred to as the CYTOX module. Baseline expression levels of CYTOX genes in PBMCs was negatively associated with responsiveness to PCV13 (for example, for *NCAM1*, *R* = −0.82 and *P* = 0.00038) but not responsiveness to PPSV23 (Fig. 3f). In addition, on average, PBMCs from men showed higher expression of CYTOX genes than PBMCs from women in both cohorts (Fig. 3g and Supplementary Table 6a). Expression levels of the top CYTOX genes increased with age, albeit not significantly (Fig. 3h); age-related upregulation of these genes was also observed in a larger cohort (*n* = 41 men and 34 women; *P* < 0.05 (ref. ^27^); Extended Data Fig. 6b). A different module (dark green, 134 genes) was specifically associated with reduced PPSV23 responses (*P* = 0.05; Extended Data Fig. 6a,c and Supplementary Table 6b), including genes associated with the cell cycle and transcriptional regulation (*ANGEL2*, *MRE11*, *MTERF1*, *NUP107* and *YTHDC2*); however, they were not associated with any immune cell type or function. The top genes in this module showed no significant association with age and sex (Extended Data Fig. 6d,e). To summarize, WGCNA of the baseline PBMC RNA-seq data revealed two distinct gene sets that are significantly associated with PCV13 and PPSV23 responses.

### The CYTOX signature stems from CD16^+^ NK cells

**Fig. 4:**
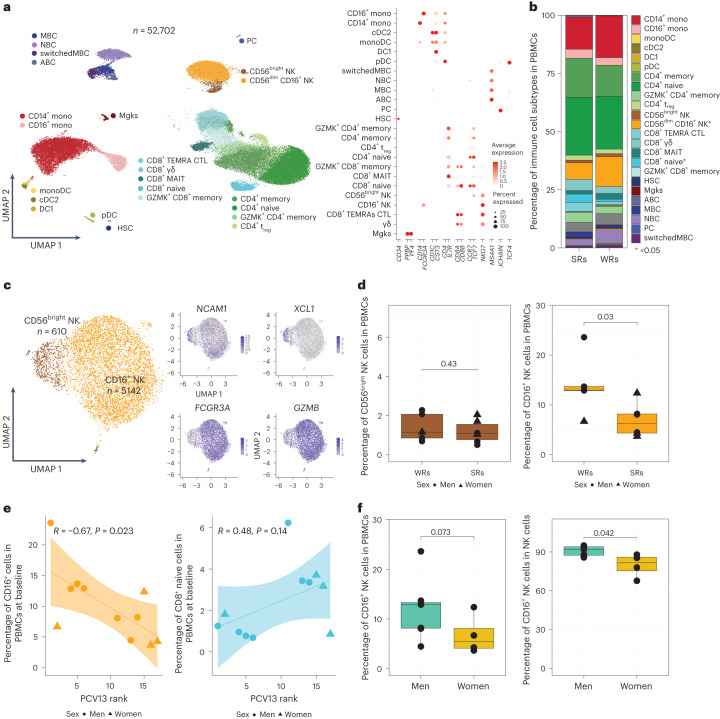
CD16^+^ NK cell frequency in PBMCs is negatively associated with PCV13 vaccine responses. **a**, Uniform manifold approximation and projection (UMAP) of PBMCs from 11 PCV13 donors (six SRs and five WRs) showing 24 clusters from 52,702 cells colored by immune cell type. Immune subsets were identified in a supervised manner. Lineage markers are shown in the dot plot; MBC, memory B cell; NBC, naive B cell; ABC, age-associated B cell; mono, monocyte; monoDC, monocyte-derived DC; HSC, hematopoietic stem cell; Mgk, megakaryocyte; MAIT, mucosal-associated invariant T cell; GZMK, granzyme K; PC, plasma cells; CTL, cytotoxic T cell. **b**, Stacked bar plot of immune cell frequencies in SRs and WRs. The cell types with significant differences in their frequencies between SRs and WRs are marked with a red asterisk (*; *P* < 0.05). **c**, UMAP of NK cell subsets with feature plots showing the expression *NCAM1*, *XCL1*, *FCGR3A* and *GZMB* in blue, highlighting the two NK populations: CD56^dim^CD16^+^ NK cells and CD56^bright^ NK cells. **d**, Box plots of CD16^+^ NK cell and CD56^bright^ NK cell frequencies in SRs (*n* = 6) and WRs (*n* = 5). **e**, Correlation analysis between PCV13 rank and prevaccination frequencies of CD16^+^ NK and CD8^+^ naive T cells (*n* = 11). **f**, Sex differences in the prevaccination percentages of CD16^+^ NK cells in total PBMCs and in total NK cells (*n* = 11). Box plots display the median and IQR (25–75%), with whiskers representing the upper and lower quartiles ±1.5× IQR. A Wilcoxon rank-sum test (two sided) was used to compare cell percentages between SRs and WRs (**b** and **d**) and CD16^+^ NK cell percentages between men and women (**f**). Correlations were computed using the Pearson correlation metric (**e**), and *P* values were computed using two-sided *t*-tests; *n* represents the number of biological replicates. Source data

**Extended Data Fig. 7:**
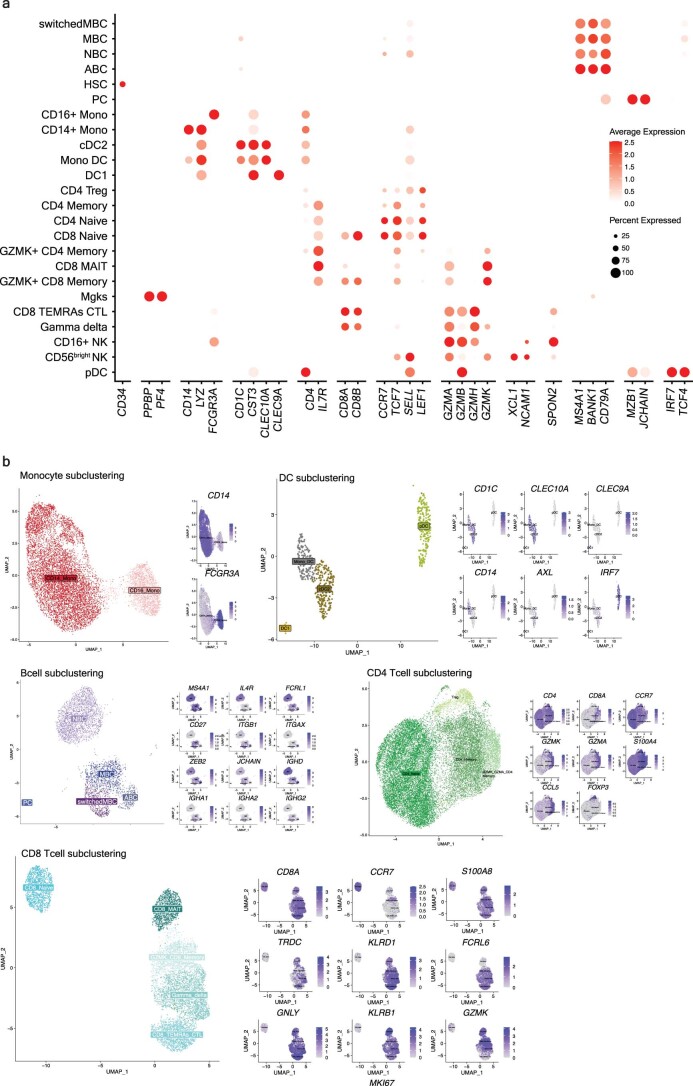
Subclustering of baseline PBMC RNA-Seq at the single-cell level. **a)** Dot plot showing the marker gene expression for each subset detected in the PBMC scRNA-seq data**. b)** Single cell subclustering of Monocytes, DCs, B, CD4 and CD8 T cells. Feature plots showing the known markers in blue.

**Extended Data Fig. 8:**
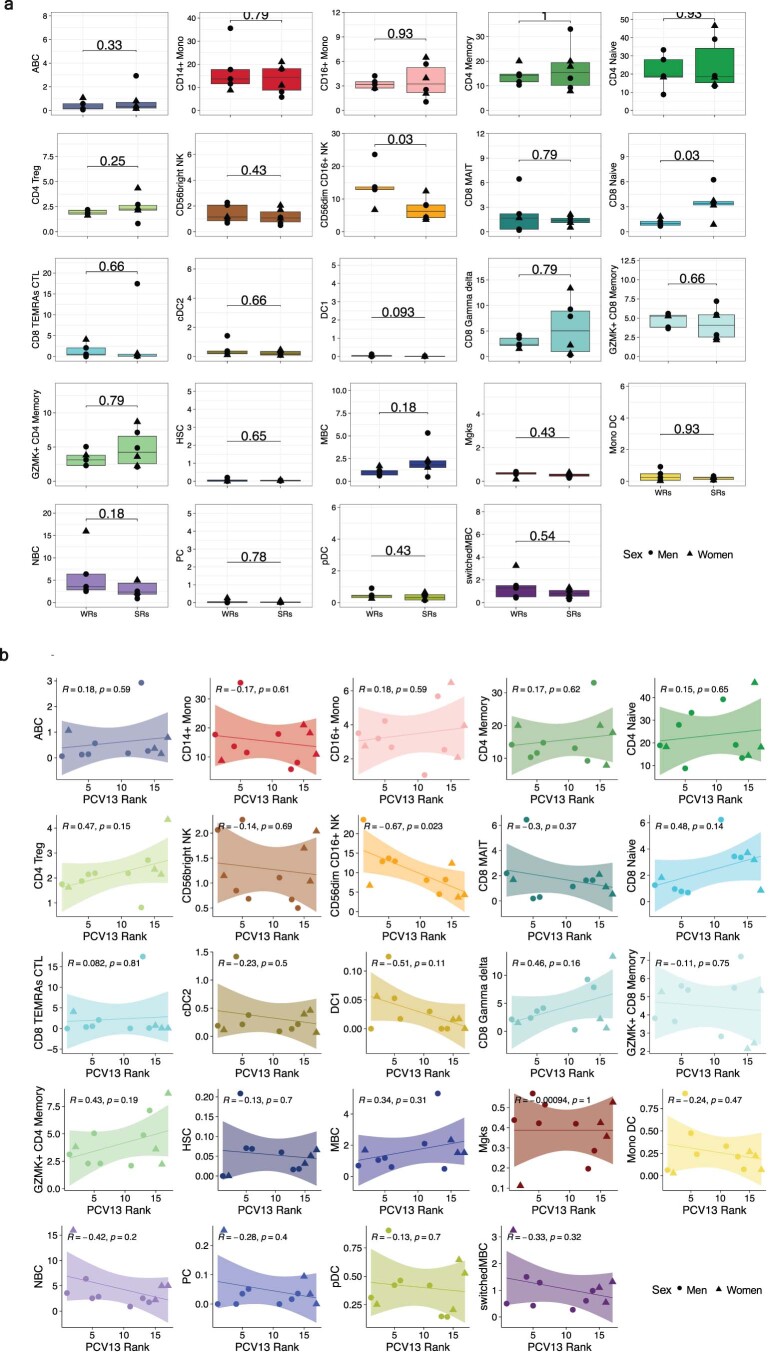
Differential immune cell frequencies between PCV13 SRs and WRs. **a**) Stacked bar plot showing immune cell compositions in strong responders (SRs, n = 6) and weak responders (WRs, n = 5). Cell types showing significant difference between SRs and WRs are marked with a star (pvalue < 0.05 starred in red). Boxplot showing the frequency of different immune cell subsets in SRs and WRs. **b**) Correlation analysis between PCV13 rank and the frequency of different immune cell subsets. Boxplots display the median and IQR (25–75%), with whiskers representing the upper- and lower-quartile ±1.5× IQR. The Wilcoxon Rank sum test (two-sided) was used to compare the cell percentages between PCV13- SRs and WRs. Correlation analysis was performed using the Pearson correlation metric and p-value was computed using two-tailed t-test. The ‘n’ represents number of biological replicates. Source data

To identify cells bearing the CYTOX signature, we generated single-cell RNA-seq (scRNA-seq) data from prevaccination PBMCs of 11 PCV13 donors (6 SRs and 5 WRs), resulting in 52,702 cells after quality control (Fig. 4a and Supplementary Table 7). Clustering of PBMCs generated 24 clusters, which were annotated into monocytes (CD14 and CD16), DCs (monocyte-derived DCs, cDC1s and cDC2s), B cells (naive cells, memory cells, switched memory B cells, age-associated B cells and plasma cells), CD4^+^ T cells (naive, memory, regulatory T (T_reg_) and cytotoxic CD4^+^ T cells), CD8^+^ T cells (naive and memory cells (granzyme K^+^ T cells, effector memory T cells expressing CD45RA (TEMRA), mucosal-associated invariant T cells and γδ T cells)) and NK cells (CD56^bright^CD16^−^ and CD56^dim^CD16^+^; Fig. 4a and Extended Data Fig. 7a,b). Among these, only two cell subsets were significantly different between SRs and WRs (Fig. 4b and Extended Data Fig. 8a), where SRs had more naive CD8^+^ T cells (*P* = 0.03) and fewer CD16^+^ NK cells (*P* = 0.03; Fig. 4b and Extended Data Fig. 8a).

**Extended Data Fig. 9:**
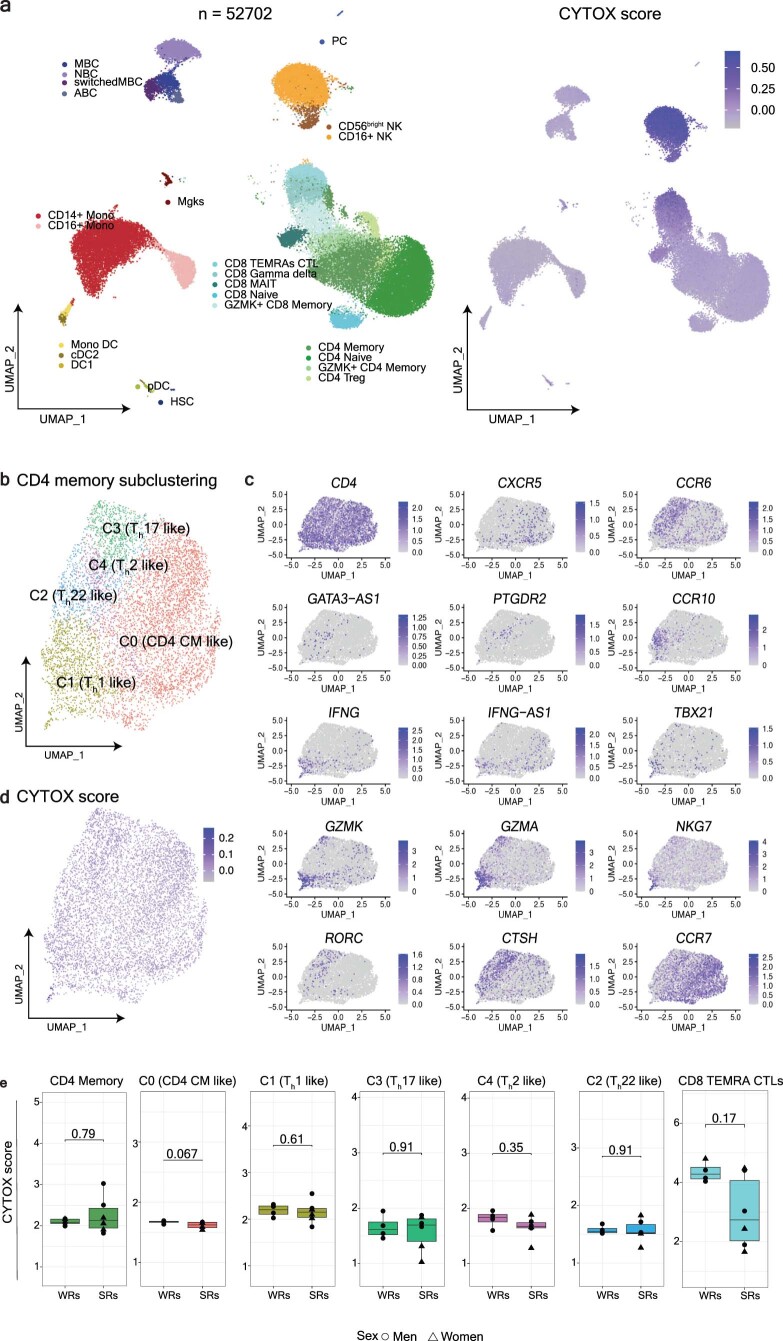
The CYTOX Signature stems from CD16+ NK Cells. **a)** UMAP representation of PBMCs derived from 11 PCV13 donors, consisting of 6 strong responders (SRs) and 5 weak responders (WRs). This visualization encompasses 24 clusters from a total of 52,702 cells, each color-coded by their respective immune cell type. An accompanying feature plot elucidates the distribution of the CYTOX score across these 24 distinct immune cell populations. The CYTOX score is derived from the expression of genes from module associated with PCV13 response (midnightblue module), showing a Pearson correlation coefficient greater than 0.5. **b)** Subclustering of CD4^+^ memory cells highlight subsets expressing marker genes for C0 (central memory like cells), C1 (T_h_1 like), C2 (T_h_22 like), C3(T_h_17 like) and C4 (T_h_2 like). **c)** A feature plot to show marker gene expression for memory CD4^+^ subsets. **d)** A feature plot showing CYTOX score for memory CD4^+^ T cells. **e)** Comparison of CYTOX scores for 6 SRs and 5 WRs for each memory CD4+ subset and CD8+ TEMRA CTLs. Boxplots display the median and IQR (25–75%), with whiskers representing the upper- and lower-quartile ±1.5× IQR. The Wilcoxon Rank sum test (two-sided) was employed to assess differences in the CYTOX scores between the PCV13 SRs and WRs across these CD4 memory subsets and CD8^+^ TEMRA CTLs. The ‘n’ represents number of biological replicates. Source data

Blood NK cells include immature CD56^bright^CD16^−^ NK cells that produce the chemokine XCL1 and the mature, highly cytotoxic CD56^dim^CD16^+^ cells, both of which were captured in the scRNA-seq data (Fig. 4c). However, only the CD16^+^ NK cells were significantly associated with vaccine responsiveness, where WRs had higher frequencies of these cells (Fig. 4d,e and Extended Data Fig. 8b). In line with their stronger PCV13 responses (Fig. 1f), women had lower CD16^+^ NK cell frequencies than men (*P* = 0.042; Fig. 4f). We calculated CYTOX scores at single-cell resolution using the average expression of CYTOX genes. The CYTOX score was highest in the NK cell populations, followed by the cytotoxic CD8^+^ T cell populations (Extended Data Fig. 9a). There was no significant difference between the CYTOX scores of SRs and WRs neither for the memory CD4^+^ T cell clusters (Extended Data Fig. 9b–e) nor for other cell types, including TEMRAs that have high CYTOX scores (Extended Data Fig. 9e). Together, these data establish that CD16^+^ NK cells comprise the population of cells that bears the CYTOX signature, where the increased frequency of CD16^+^ NK cells is associated with reduced PCV13 responses.

**Fig. 5:**
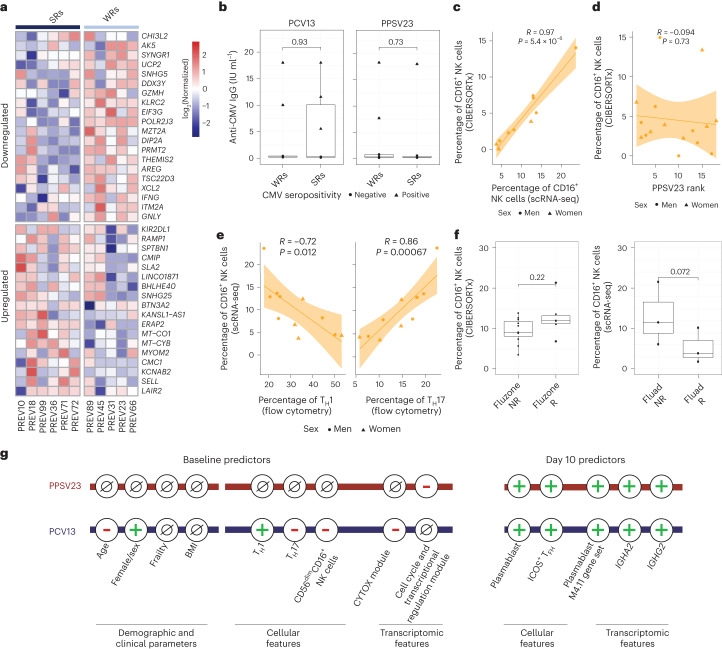
Increased cytotoxicity in CD16^+^ NK cells of PCV13 WRs. **a**, Heat map of the differentially expressed genes in CD16^+^ NK cells of six PCV13 SRs and five WRs at baseline, as assessed using normalized expression values from the scRNA-seq pseudobulk analysis. **b**, Box plots comparing anti-CMV IgG titers between SRs and WRs for PCV13 (left) and PPSV23 (right). **c**, Correlation analysis of prevaccination CD16^+^ NK cell percentages estimated by scRNA-seq and CIBERSORTx (*n* = 11). **d**, Correlation analysis of CIBERSORTx-based estimates of CD16^+^ NK cells and PPSV23 rank (*n* = 16) at baseline. **e**, Correlation analysis of CD16^+^ NK cell percentages determined by scRNA-seq and T_H_1 and T_H_17 cell percentages determined using flow cytometry at baseline. **f**, Box plots of prevaccination CD16^+^ NK cell percentages in Fluad responders (R; *n* = 3) and non-responders (NR; *n* = 3) and Fluzone trivalent inactivated influenza vaccine responders (*n* = 5) and non-responders (*n* = 11). **g**, Summary schema showing the demographic, clinical, cellular and transcriptomic parameters associated with PCV13 and PPSV23 vaccine responsiveness at baseline and day 10. Box plots display the median and IQR (25–75%), with whiskers representing the upper and lower quartiles ±1.5× IQR. A Wilcoxon rank-sum test (two sided) was used to compare the mean anti-CMV IgG titers between SRs and WRs of PCV13 and PPSV13 donors (**b**) and prevaccination CD16^+^ NK cell percentages in Fluad responders and non-responders and Fluzone responders and non-responders (**f**). Correlation analyses were computed using the Pearson correlation metric (**c**, **d** and **e**), and *P* values were computed using two-sided *t*-tests; *n* represents the number of biological replicates. Source data

Differential gene expression analyses between CD16^+^ NK cells of SRs and WRs revealed 38 differentially expressed genes (FDR of 5%; Fig. 5a and Supplementary Table 8). CD16^+^ NK cells from WRs showed higher expression of cytotoxic genes (*GNLY* and *GZMH*) and *IFNG*, indicating a more ‘cytotoxic’ and ‘activated’ phenotype. Furthermore, WR CD16^+^ NK cells expressed higher levels of the inhibitory receptor gene *KLRC2* (*NKG2C*), suggesting a more advanced maturation stage^28^. CD16^+^ NK cells from WRs also overexpressed amphiregulin (*AREG*), a ligand for the epidermal growth factor receptor that is upregulated after inflammation and mitochondrial stress in immune cells^29^. Although CMV can modulate NK cell phenotypes^30^, CMV positivity (10 of 39 donors) was not associated with responsiveness to either vaccine (Fig. 5b). These data suggest that CD16^+^ NK cells from PCV13 WRs had a more cytotoxic and activated transcriptional phenotype than the same cells from SRs.

### PPSV23 responses are independent of CD16^+^ NK cell frequency

**Extended Data Fig. 10:**
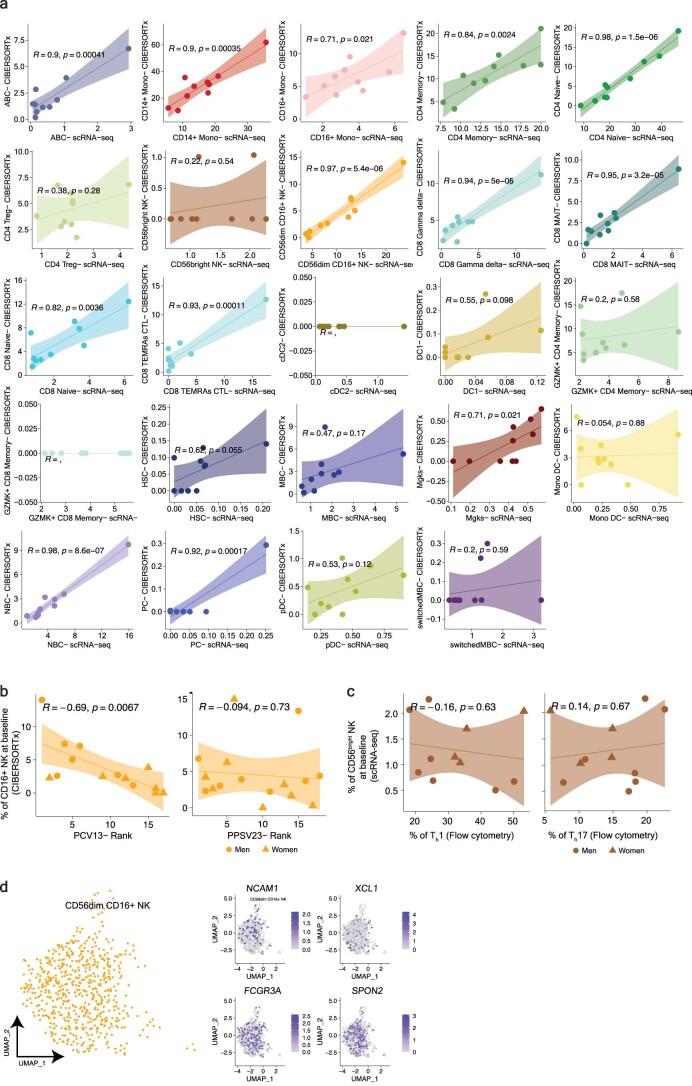
Association between baseline CD16^+^ NK frequency estimated from CIBERSORTx and PCV13 vaccine responses. **a**) Correlation between pre-vaccination cell frequency estimated by scRNA-seq and CIBERSORTx for different immune subtypes. **b**) Correlation analysis of CD16^+^ NK frequency at baseline and vaccine responsiveness Ranks of PCV13 (n = 14) and PPSV23 (n = 16), respectively. For the correlation analysis with PPSV23-Rank, cell frequency estimated by CIBERSORTx from the bulk PPSV23 baseline transcriptomes (n = 16) was considered. **c**) Correlation analysis of CD56^bright^ NK frequency (n = 11) and T_h_1, and T_h_17 frequency at baseline. Pearson correlation was used to perform correlation analysis (a,b,c) and p-value was computed using two-tailed t-test. **d**) UMAP representation of CD16^+^ NK subsets in fluad responders (n = 3) and non-responders (n = 3) at baseline. Feature plots showing *NCAM1*, *XCL1*, *FCG3RA* and *SPON2* in CD16^+^ NK cells in blue. Correlation analysis was performed using the Pearson correlation metric and p-value was computed using two-tailed t-test. The ‘n’ represents number of biological replicates. Source data

To establish whether CD16^+^ NK cells are also associated with PPSV23 responsiveness, we used CIBERSORTx and inferred cell-type frequency from bulk data by using cell-type-specific expression from scRNA-seq data (Fig. 4a). First, we confirmed that inferred CD16^+^ NK cell frequencies match scRNA-seq-based frequencies using 11 donors for whom we have both scRNA-seq and bulk PBMC RNA-seq data (*R* = 0.97, *P* = 5.4 × 10^–6^; Fig. 5c and Extended Data Fig. 10a). In the PCV13 cohort, inferred CD16^+^ NK cell frequency significantly correlated with responsiveness (*R* = −0.69, *P* = 0.0067), reinforcing our observation. By contrast, the CD16^+^ NK cell frequency was not associated with PPSV23 responsiveness (*R* = −0.094, *P* = 0.73; Fig. 5d and Extended Data Fig. 10b). Together with the lack of association between CYTOX genes and PPSV23 responsiveness (Fig. 3e), these analyses established that CD16^+^ NK cells are important only for responses to the conjugated PCV13 vaccine. Notably, donors with higher CD16^+^ NK cell frequencies also had higher T_H_17 cell frequencies (*R* = 0.86, *P* = 0.00067) and lower T_H_1 cell frequencies (*R* = −0.72, *P* = 0.012; Fig. 5e), which was not observed for CD16^−^ NK cells (Extended Data Fig. 10c).

To study the role of CD16^+^ NK cells in responses to other vaccines in older adults, we reanalyzed bulk PBMC RNA-seq data from the previously published Fluzone study (*n* = 16, 65+ years old)^31^. CIBERSORTx-inferred CD16^+^ NK cell frequencies did not show significant differences between responders and non-responders (*P* = 0.22; Fig. 5f). However, reanalyses of scRNA-seq data from individuals treated with the adjuvanted influenza vaccine Fluad (*n* = 6, 65 years old)^32^ showed that CD16^+^ NK cell frequencies were higher in non-responders than in responders (three responders and three non-responders; *P* = 0.072; Fig. 5f). Together, these data suggest that the CD16^+^ NK cell phenotype and the CYTOX signature are specifically associated with the conjugated pneumococcal vaccine PCV13. Furthermore, the CYTOX signature might also be predictive of responses for other vaccines (for example, Fluad) and might provide an effective strategy for precision vaccinology among older adults (Fig. 5g). Associations between this signature and other vaccines should be studied further in larger cohorts.

### Excluding non-informative donors strengthens baseline associations

**Fig. 6:**
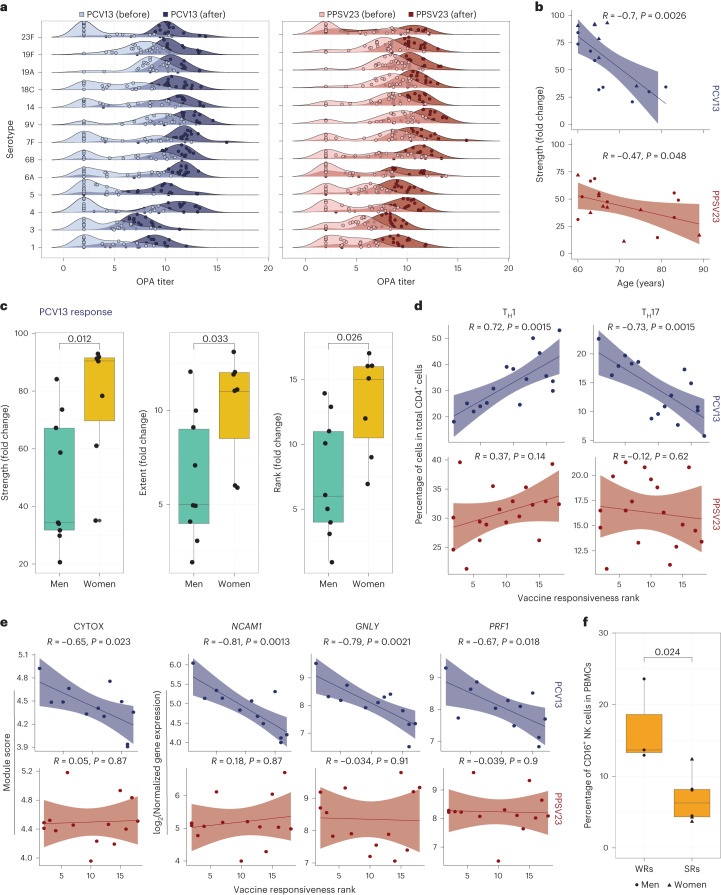
Association between demographic, cellular and transcriptomic parameters and vaccine responsiveness after the exclusion of non-informative donors. **a**, Ridge plot displaying the distribution of before and after vaccination OPA titers for each serotype in the PCV13 (*n* = 19) and PPSV23 (*n* = 20) cohorts. Note that baseline titer levels vary among serotypes. Non-informative donors were identified using a published strategy, and associations were recalculated after exclusion of these donors. **b**, Association between PCV13 (*n* = 16) and PPSV23 (*n* = 18) strength and age (in years). **c**, Sex differences in PCV13 (*n* = 16) and PPSV23 (*n* = 18) vaccine responses. Note that women mount significantly stronger responses to the PCV13 vaccine. **d**, Association between T_H_1 and T_H_17 cell percentages at baseline and PCV13 (*n* = 15) and PPSV23 (*n* = 17) vaccine responsiveness. **e**, Correlations between CYTOX scores at baseline and PCV13 (*n* = 12) and PPSV23 (*n* = 14) vaccine responsiveness (left) and correlations between the baseline expression of *NCAM1*, *GNLY* and *PRF1* and PCV13 and PPSV23 vaccine responsiveness (right). **f**, Baseline abundance of CD16^+^ NK cells in PCV13 SRs (*n* = 6) and WRs (*n* = 3). Correlation analyses were computed using the Pearson correlation metric (**b** and **d**–**f**), and *P* values were computed by using two-sided *t*-tests. Box plots display the median and IQR (25–75%), with whiskers representing the upper and lower quartiles ±1.5× IQR. A Wilcoxon rank-sum test (two sided) was used to compare strength, extent and rank between men and women treated with PCV13 and PPSV23 (**c**); *n* represents the number of biological replicates. Source data

Previous studies of pneumococcal vaccines showed that donors with high baseline antibody levels have lower fold increases after vaccination^33^ (Extended Data Fig. 2a,b). Such donors are likely already protected and can confound conclusions related to vaccine responsiveness and efficacy. However, identification of non-informative (high baseline) donors is particularly challenging for bacterial pneumonia vaccines due to the variability in serotype baselines; some serotypes have consistently higher baselines (9V, 19A and 19F in our cohort; Fig. 6a) due to the variability in baseline titer levels among different cohorts^23^. To overcome this, we previously developed an analytical strategy to bin donors into three groups: SRs (low baseline, high fold increase in titers), WRs (low baseline, low fold increase) and non-informative donors (high baseline titers)^23^. Using this strategy, we identified 10 SRs, 6 WRs and 3 non-informative donors in the PCV13 arm and 11 SRs, 7 WRs and 2 non-informative donors in the PPSV23 arm (Supplementary Table 9a,b).

We excluded the non-informative donors and repeated all the analyses, which confirmed and, in some instances, strengthened all the associations observed with vaccine responsiveness. In summary, there was a significant age-related decline in responsiveness to both PCV13 (*R* = −0.7, *P* = 0.0026) and PPSV23 (*R* = −0.47, *P* = 0.047) vaccines (Fig. 6b and Supplementary Table 9c,d). Women mounted stronger responses to PCV13 than men across the three metrics strength (*P* = 0.012), extent (*P* = 0.033) and rank (*P* = 0.026; Fig. 6c and Supplementary Table 9c), which was not observed for PPSV23 (Supplementary Table 9d). Baseline frequencies of T_H_1 (*R* = 0.72, *P* = 0.0015) and T_H_17 (*R* = −0.73, *P* = 0.0013) cells correlated with PCV13 responses (Fig. 6d) but not with PPSV23 responses. The baseline CYTOX module score and expression of its top genes (*NCAM1*, *GNLY* and *PRF1*) were negatively associated specifically with PCV13 responses (*R* = −0.65, *P* = 0.0023; Fig. 6e). Moreover, men showed higher expression of CYTOX genes than women (Supplementary Table 9c). Finally, PCV13 WRs had higher baseline frequencies of CD16^+^ NK cells than SRs (*P* = 0.024; Fig. 6f). The dark green module score and baseline expression of its top genes (*ANGEL2* and *ZNF529*) negatively correlated specifically with PPSV23 responses (Supplementary Table 9d). These new analyses suggest that, even among pneumococcal vaccine-naive older adults, 10–15% of the cohort have high baseline titers. The demographic, baseline cellular and transcriptional features associated with responsiveness to PCV13 and PPSV23 are independent of these non-informative donors.

## Discussion

Older adults are at high risk for morbidity and mortality due to infectious diseases^1^, including those caused by *S. pneumoniae*. Despite the availability of two types of FDA-approved pneumococcal vaccines, responsiveness of older adults to these vaccines remains poorly characterized. We studied the responses of 39 older adults to PPSV23 or conjugated PCV13 and uncovered (1) strong antibody responses to both vaccines and transcriptional activation of plasmablast genes at day 10, (2) increased ICOS^+^ T_FH_ cells at day 10 for both vaccines, (3) a baseline immune phenotype composed of increased frequencies of T_H_17 cells, reduced frequencies of T_H_1 cells and increased frequencies of CD16^+^ NK cells that is specifically associated with PCV13 responses and is more frequently observed in men than in women and (4) a distinct baseline gene expression module that is associated with PPSV23 responses.

Both vaccines induced strong antibody responses, although PCV13 induced higher increases in OPA titers than PPSV23, in line with observations in younger adults^34^. Both vaccines induced strong responses to the 12 shared serotypes. Although PPSV23 lacks serotype 6A, some donors showed increased antibody responses to this serotype, potentially due to the cross-reactivity with serotype 6B. The strongest transcriptional signal was the upregulation of plasmablast genes, in particular immunoglobulins, at day 10. Both vaccines predominantly induced the expression of the heavy chain constant region genes encoding IgG2 and IgA2 isotypes, distinct from influenza vaccine responses (IgG1 and IgG3). A recent study on PCV13 and PPSV23 (ref. ^24^) showed an increased abundance of these isotypes 30 d after vaccination with either vaccine. Despite the strong transcriptional upregulation of plasmablast genes at day 10, increases in the numbers of circulating plasmablasts were marginal. It is possible that 10 d after vaccination was not the ideal time point to detect peak plasmablast responses or the responses were weaker in older adults. Indeed, in a previous study, we showed that, after PCV13 vaccination^11^, antigen-specific (AIM^+^) T_FH_ cells were expanded in younger donors but not in older donors.

The release of plasma cells into circulation is linked to the frequencies of activated T_FH_ cells that positively correlate with serological responses to influenza vaccine and are affected by aging^35^. For pneumococcal vaccines, there was a significant expansion of ICOS^+^ T_FH_ cells at day 10; however, this expansion did not correlate with antibody responses, which could be due to age of our donors or the timing of our T_FH_ measurement (day 10 versus day 7). The expansion of ICOS^+^ T_FH_ cells in response to PPSV23 (at comparable levels to PCV13) suggests that PPSV23 also induces T cell immunity. A lower frequency of T_H_1 cells and higher frequency of T_H_17 cells at baseline was associated with weaker responses to PCV13. Age-dependent increases in T_H_17 cells or increased production of IL-17 have been observed^36,37^. The frequencies of these cell types were not significantly associated with age, although a significant sex association was observed. Women had higher T_H_1 and lower T_H_17 cell frequencies than age-matched men, which might explain their stronger responses to PCV13. Proinflammatory molecules (IL-6 and IL-1β) that promote T_H_17 cell differentiation^38^ are also linked to systemic chronic inflammation with aging (that is, inflammaging)^39^. Accelerated inflammaging in men^27^ might contribute to the observed sex differences in PCV13 responses. In contrast to our previous study in young adults^19^, we did not detect an innate response at day 1 for either vaccine due to the already elevated myeloid signatures in older individuals or to an age-specific altered response to these vaccines.

The cell types uncovered here (T_H_17, T_H_1 and CD16^+^ NK cells) go through significant age-related changes. Although the global proportion of T_H_17 cells among memory CD4^+^ T cells declines with age^40^, a significant increase in the T_H_17:T_reg_ cell ratio was observed in older individuals^36^. Additionally, when activated in vitro, CD4^+^ naive T cells from older individuals show enhanced T_H_17 differentiation compared to CD4^+^ naive T cells from younger individuals. The proportion of NK cells among peripheral blood lymphocytes increases with age^41–43^. Our study shows that a higher proportion of CD16^+^ NK cells is associated with worse responses to PCV13 in older individuals, highlighting the significance of NK cell aging in the context of age-associated decline in vaccine responses.

Baseline bulk RNA-seq data revealed a cytotoxicity-associated gene set (CYTOX) that predicted reduced responses to conjugated PCV13, and scRNA-seq data indicated that this CYTOX signature stems from mature CD16^+^ NK cells that are more cytotoxic and have antibody-dependent cellular cytotoxicity functions^26^. Although antibody-dependent cellular cytotoxicity-proficient antibody production is a key correlate of vaccine responses, including for bacterial pneumonia vaccines^24^, activated NK cells might be detrimental for vaccine responses. For the yellow fever vaccine YF-17D, the frequency of activated NK cells at day 7 was linked to reduced neutralizing antibody levels^44^. For malaria vaccines, blood transcriptional modules associated with NK cells displayed a strong negative correlation with antibody response and protection at multiple time points^45^.

CD16^+^ NK cells from WRs expressed cytotoxicity-related genes at higher levels than those from SRs. These cytotoxic molecules and some NK receptors are also upregulated with age in CD8^+^ TEMRA cells, which was captured in the scRNA-seq data yet was not associated with PCV13 responses. Interestingly, CD16^+^ NK cell frequency significantly correlated with T_H_ cell subsets associated with PCV13 responses. Donors who had higher frequencies of CD16^+^ NK cells had higher T_H_17 cell frequencies and lower T_H_1 cell frequencies. Whether this reflects a parallel co-regulation of these three subsets in older donors or whether CD16^+^ NK cells contribute to the imbalance of T_H_1 and T_H_17 cells remains to be established. The role of NK cells in modulating T cells has been explored in autoimmunity, transplantation, pregnancy and infection^46–50^. These studies concluded that NK cells can modulate T_H_17 and T_H_1 cell expansion via IL-17A or interferon-γ secretion or by direct killing of activated helper T cells. Additionally, helper T cell killing by NK cells is sensitive to helper T cell-secreted IL-2 quantity and helper T cell expression of HLA-E (inhibitory) or NKG2D or DNAM-1 ligands (stimulatory). Mouse studies showed that NK cells can kill helper T cells and T_FH_ cells in certain viral infection settings, resulting in worse cytotoxic T cell responses and reduced germinal center formation, B cell affinity maturation and neutralizing antibody titers^46,51–54^. Indeed, NK cell depletion before vaccination increased vaccine-induced antibody responses in mice^53^.

Capsular polysaccharidic antigens in pneumococcal vaccines can neutralize preexisting antibodies or paralyze B cells, potentially rendering a vaccine more susceptible to infections^55–57^. This could, in theory, contribute to an increased incidence of pneumonia (although not death) in vaccinated older adults. However, pneumococcal conjugated vaccines are shown to be effective in preventing invasive disease in older adults^58^ and in human immunodeficiency virus (HIV)-infected adults^59^. In agreement, a meta-analysis of 18 randomized trials (*n* = 64,500 donors) showed that pneumococcal polysaccharide vaccines reduce the risk of both invasive and noninvasive pneumococcal disease^60^.

Here, we present a precision vaccinology study of pneumococcal vaccine responses in older adults. Remarkably, the baseline predictors for the two vaccines were distinct, despite that both vaccines include the same polysaccharidic antigens. Our study provides a framework for stratification of older adults for the administration of pneumococcal vaccines, with potential implications for other vaccines, by establishing blood-based clinical assays to assess whether an individual has the CYTOX signature (for example, by quantifying the expression of *NCAM1* by using quantitative real-time PCR or the frequency of CD16^+^ NK cells by using flow cytometry). As the CYTOX signature only affects responsiveness to PCV13, donors with a high CYTOX signature likely benefit from PPSV23, whereas donors with a low CYTOX signature likely benefit from PCV13. Future studies are needed to establish whether these baseline signatures are also predictive in other populations. Our data also show the importance of biological sex for vaccine administration; women had stronger responses to conjugated PCV13 as they had lower CYTOX activity than age-matched men. These findings might also yield novel strategies to change baseline immune phenotypes to alter vaccine responses^61,62^, for example, by temporarily blocking the immunosuppressive or cytolytic functions of CD16^+^ NK cells before vaccination^63^. Strategies such as activating receptor blockade or epigenetic remodeling of NK cell effector functions hold potential for modulating NK cell cytotoxicity. However, these strategies are still in their infancy, and their safety and cell-type specificity are not clear.

### Study limitations

We acknowledge several limitations of our study. First, due to the frequency of the early visits, we collected blood at day 10 to study adaptive responses, which might miss the peak adaptive responses. Future longitudinal studies should investigate adaptive responses of older adults at additional time points. Second, we used OPA assays to quantify vaccine responses because they (1) mimic functional immune responses, (2) are considered state of the art for FDA approvals and (3) are more effective than ELISAs among older adults^21^. However, this decision came at a cost, and we did not have enough material to quantify OPA titers for all 23 serotypes. We therefore prioritized the most prevalent 13, which are the ones selected for the PCV13 vaccine formulation^22,64^. Although our quantification for PPSV23 closely captures donor protection from the most prevalent serotypes and is a good proxy for overall responsiveness, it does not fully capture the immune responses to all 23 serotypes. Third, due to limitations in material, we could not study the B and T cell memory responses in depth (that is, antigen-specific cells and their responses). Future studies are needed to further explore B and T cell responses of older adults to these vaccines.

## Methods

### Participant recruitment

This study was conducted following approval by the UConn Health Center Institutional Review Board (16-071J-1) and registration on ClinicalTrials.gov (NCT03104075). The complete information on clinical trial registration, study protocol, data collection and outcomes can be accessed via http://clinicaltrials.gov/study/NCT03104075. All participants provided informed consent and were compensated for their time and study visits. Blood samples were obtained from 39 healthy volunteers residing in the Greater Hartford, CT, region recruited by the UConn Center on Aging Recruitment and Community Outreach Research Core (http://health.uconn.edu/aging/research/research-cores/). Volunteers were vaccinated from May to early fall of 2017–2018 to avoid potential overlap with seasonal peak periods of influenza vaccination and infection. None of the participants reported a history of previous pneumonia. Recruitment criteria were selected to identify individuals representing ‘usual healthy’ aging, aligning with the 2019 National Institutes of Health (NIH) Policy on Inclusion Across the Lifespan (NOT-98-024). This selection would increase the generalizability of our study and translate to the general population. Inclusion criteria included an age of 60 years or older and willingness to receive PCV13 (Wyeth/Pfizer) or PPSV23 (Merck) vaccination via random assignment. Exclusion criteria included prior PCV13 or PPSV23 vaccination or a history of adverse reactions to these or any diphtheria toxoid-containing vaccine. Individuals were also excluded if they had a recent fever, received a Zostavax (shingles) vaccine in the previous 4 weeks or had notably confounding comorbidities, such as diabetes mellitus, active malignancy or recurrence in the last 5 years, congestive heart failure, unstable cardiovascular disease in the last 6 months, renal failure, impaired hepatic function, autoimmune diseases (for example, rheumatoid arthritis, lupus and inflammatory bowel disease), recent trauma or surgery, HIV or other immunodeficiency, current substance or alcohol abuse and usage of medications that alter the immune system (for example, prednisone ≥10 mg).

### Sample collection and PBMC isolation

Thirty-nine donors (20 men and 19 women) were randomly assigned to receive either PCV13 or PPSV23 at the University of Connecticut Health at Farmington, CT. Peripheral blood was collected at baseline (before vaccination at day −7) and after vaccination (days 1, 10, 28 and 60). Data collection and analysis were not performed blind to the conditions of the experiments. Detailed information on participant demographics is provided in Supplementary Table 1a,b. PBMCs were isolated from blood collected in ACD tubes using a Lymphoprep gradient (StemCell Technologies), while serum was isolated from blood collected in red-top collection tubes (BD Vacutainer). Both PBMCs and serum were cryopreserved before subsequent analyses.

### OPA titer measurements

Multiplexed opsonization assays were performed for 13 serotypes (1, 3, 4, 5, 6A, 6B, 7F, 9V, 14, 18C, 19A, 19F and 23F) across 39 donors, as previously described^22^. Frozen aliquots of target pneumococci were thawed, washed twice with Opsonization Buffer B (Hanks’ balanced salt solution with Mg^2+^/Ca^2+^, 0.1% gelatin and 10% fetal bovine serum (pH 7.2–7.4)) by centrifugation (12,000*g*, 2 min) and diluted to the proper bacterial density (~2 × 10^5^ colony-forming units per ml of each serotype). Equal volumes of four chosen bacterial suspensions were pooled for analysis. All serum samples were preincubated at 56 °C for 30 min, followed by serial dilutions in Opsonization Buffer B. Serially diluted serum (20 μl per well) was mixed with 10 μl of bacterial suspension in each well of a round-bottom 96-well plate.

After a 30-min incubation at 25 °C with shaking at 700 r.p.m. on a mini orbital shaker (Bellco Biotechnology), 10 μl of 3- to 4-week-old rabbit complement (PelFreeze Biologicals) and 40 μl of HL60 cells (4 × 10^5^ cells) were added to each well. HL60 cells (ATCC clone CCL-240) were differentiated to granulocytes by culturing in RPMI-1640 with 10% fetal bovine serum and 1% l-glutamine and 0.8% dimethylformamide at a starting density of 4 × 10^5^ cells per ml for 5–6 d. Plates were incubated in a tissue culture incubator (37 °C, 5% CO_2_) with shaking at 700 r.p.m. After a 45-min incubation, plates were placed on ice for 10–15 min, and an aliquot of the final reaction mixture (10 μl) was spotted onto four different THY agar plates (Todd–Hewitt broth with 0.5% yeast extract and 1.5% agar). When the fluid was absorbed into the agar, an equal volume of an overlay agar (THY with 0.75% agar and 25 mg liter^–1^ triphenyl tetrazolium chloride) containing one of the four antibiotics was applied to each THY agar plate. After an overnight incubation at 37 °C, the number of bacterial colonies in the agar plates was enumerated. Opsonization titers were defined as the serum dilution that kills 50% of bacteria.

### OPA titer ranking methods

Three different antibody response quantification strategies were developed: rank, strength and extent. Individuals in a cohort were ranked based on vaccine responsiveness across all serotypes using the dense ranking method. For each serotype, participants were assigned a dense rank according to their fold change (after vaccination/before vaccination) in OPA titer levels. Participants with the same fold change levels received the same rank, with the next participants getting the subsequent rank. These individual serotype ranks were then summed to obtain an overall score for each individual. Dense ranking was then applied to these overall scores to determine the final rank for each individual. Individuals with higher fold changes in titer levels across many serotypes ranked higher than others, while those with lower levels across many serotypes received lower ranks. This ranking strategy is robust to outliers (that is, a participant with very high titer levels only for one serotype) and is based on a multivariate approach (using all 13 serotypes) to quantify vaccine responsiveness of individuals. Strength represents the sum of fold change in OPA titer levels across all serotypes and explains the dynamics of baseline and postvaccination changes per sample. Higher scores indicate stronger responses in individuals. An OPA titer of 8 or above was considered a significant response to a specific serotype, forming the basis for the extent strategy^65,66^. Extent measures the number of serotypes (out of 13) an individual responds to significantly, with higher values indicating a broader response. These measures have unique advantages. To compute the baseline-adjusted fold change for the 13 serotypes, the maxRBA function (titer package in R) with scoreFun set to ‘sum’ was used^67^. The adjusted fold change was then used to rank individuals through a dense ranking approach.

To further evaluate PPSV23 vaccine responses across all 23 serotypes, we reanalyzed OPA titer data from six donors^11^. These donors received PCV13 first, followed by PPSV23 1 year later. For baseline titers of 13 serotypes that are also in PCV13, we used day 0 measurements before any vaccination, and for the 11 PPSV23-specific serotypes, we used day 360 measurements before PPSV23 vaccination. For postvaccination titer values, we used day 388 measurements. Vaccine response metrics (strength, extent and rank) were computed for all 23 serotypes and the 13-serotype subset. Pearson correlation was used to measure the correlation between vaccine responses from the two sets.

Non-informative donors in our cohorts were identified using baseline (C1) and fold change (C2) thresholds. A baseline cutoff (C1) of 400 and a fold change cutoff (C2) of 8 were used.

Our scoring approach^23^ assigned scores of −1 for weak responses (baseline OPA titers of <C1 and fold change of <C2), 0 for non-informative responses (baseline OPA titers > C1) and 1 for strong responses (baseline OPA titer of <C1 and fold change of >C2) for each serotype. Scores were aggregated across the 13 tested serotypes for each donor. Donors who had seven or more serotypes labeled as non-informative responses were classified as non-informative donors. Others were categorized as strong or weak based on the predominant score.

### Determination of CMV seropositivity

Serum samples from 39 participants were assessed for CMV-specific IgG antibodies using an ELISA kit (Aviva Systems Biology). After thawing and centrifugation (6,000*g*, 1 min), samples and controls were diluted as per the manufacturer’s instructions. Results were calculated using nonlinear regression curve fitting based on vendor-supplied controls. Seropositivity was ≥1 IU ml^–1^.

### Flow cytometry data generation and analyses

Fluorescence-labeled antibody cocktails for different cell-surface staining panels were premixed in BD Horizon Brilliant Stain Buffer (BD Biosciences) 10 min before staining. Antibody cocktails were added over 100-μl aliquots of anticoagulated whole blood in a 5-ml FACS tube within 60 min of blood collection. Samples were incubated for 15 min at 25 °C, lysed and fixed with 2 ml of 1× FACS lysing solution (BD Biosciences) for 8 min at 25 °C. The lysed samples were washed twice to remove the unbound antibodies, lysed red blood cells and platelets and finally resuspended in 250 μl of PBS, to which 50 μl of count bead suspension (Count Bright Absolute Counting Beads, Thermo Fisher) was added for the detection of absolute cell counts. For the analysis of the CD4^+^ T cell compartment, cells were stained with fluorochrome-labeled antibodies targeting the following surface markers: CD3 AF700 (clone UCHT1, BioLegend, 1:200), CD4 APC-Cy7 (clone OKT4, BioLegend, 1:100), CD183 BV421 (clone GO25H7, BioLegend, 1:100), CD196 PE (clone 11A9, BD Biosciences, 1:100), CD185 APC (clone J252D4, BioLegend, 1:100), CD279 FITC (clone MIH4, BD Biosciences, 1:100), CD278 PE-Cy7 (clone C398.4A, BioLegend, 1:200) and CD45RA ECD (clone 3H4, Beckman Coulter, 1:100). Antibody details are provided in Supplementary Table 4a. For analysis of the DC compartment, cells were stained with fluorochrome-labeled antibodies targeting the following surface markers: lineage cocktail (Lin1) FITC (CD3, clone SK7; CD16, clone 3G8; CD19, clone SJ25C1; CD14, clone MΦP9; CD20, clone L27; CD56, clone NCAM16.2; BD Biosciences, 1:100), HLA-DR APC-eFluor780 (clone LN3, Thermo Fisher, 1:100), CD11c V450 (clone B-ly6, BD Biosciences, 1:100), CD1c PerCP-Cy5.5 (clone L161, BioLegend, 1:100), CD141 APC (clone AD5-14H12, Miltenyi Biotec, 1:100), CD303 PE (clone AC144, Miltenyi Biotec, 1:200), CD86 PE-Cy7 (clone IT2.2, BioLegend, 1:100) and CD40 APC-R700 (clone 5C3, BD Biosciences, 1:50). For analysis of the B cell compartment, cells were stained with fluorochrome-labeled antibodies targeting the following surface markers: CD3 AF700 (clone UCHT1, BD Biosciences, 1:200), CD19 ECD (clone J3-119, Beckman Coulter, 1:100), IgD FITC (clone IA6-2, BD Biosciences, 1:25), CD27 PE (clone M-T271, BD Biosciences, 1:50), CD20 APC (clone 2H7, BioLegend, 1:50), CD86 PE-Cy7 (clone IT2.2, BioLegend, 1:100), CD38 BV421 (clone HIT2, BioLegend, 1:100) and CD138 PE-Cy7 (clone MI15, BD Biosciences, 1:50). The stained cells were acquired with an LSR Fortessa X-20 (BD) and analyzed with FlowJo V9.9.6 software (Treestar). A Wilcoxon matched-pairs signed-rank test assessed differences in cell counts in response to PCV13 or PPSV23 at various time points (days 1, 10, 28 and 60) compared to baseline for high-quality control samples (*n* = 35; 19 men and 16 women). We computed the associations between baseline cellular composition and donor vaccine rank using Pearson correlations.

### RNA-seq library generation

Total RNA was isolated from PBMC samples using a Qiagen RNeasy Mini kit (Qiagen) or Arcturus PicoPure (Life Technologies) kits following the manufacturer’s protocols. During RNA isolation, DNase treatment was additionally performed using an RNase-free DNase set (Qiagen). RNA quality was checked using an Agilent 2100 Expert Bioanalyzer (Agilent Technologies). RNA quality was reported as a score from 1 to 10, and samples with <8.0 threshold were omitted from the study. RNA-seq libraries were prepared with a KAPA mRNA HyperPrep kit (Roche) according to the manufacturer’s instructions. Poly(A) RNA was isolated from 200 ng of total RNA using oligo(dT) magnetic beads and fragmented at 85 °C for 6 min, targeting fragments within the range of 250–300 base pairs. Fragmented RNA was reverse transcribed by incubation for 10 min at 25 °C, followed by incubation for 15 min at 42 °C and inactivation for 15 min at 70 °C. This was followed by second-strand synthesis and A tailing at 16 °C for 30 min and at 62 °C for 10 min. A-tailed, double-stranded cDNA fragments were ligated with Illumina unique adapters (Illumina). Adapter-ligated DNA was purified using AMPure XP beads and amplified by ten cycles of PCR amplification. The final library was cleaned up using AMPure XP beads. Quantification of libraries was performed using real-time quantitative PCR (Thermo Fisher). RNA-seq libraries were sequenced using a NovaSeq (2 × 150 base pair paired-end reads) to obtain ~100 million reads per sample.

### RNA-seq preprocessing

RNA-seq samples were processed using the The Jackson Laboratory’s in-house data analysis pipeline. Raw BCL files were converted to fastq files using CASAVA. Trimmomatic (version 0.33) removed adapters, low-quality bases and short reads, and reads were filtered out with more than 50% low-quality bases. The remaining high-quality reads were used for gene expression estimation using the EM algorithm for paired-end read data with default alignment settings. RSEM used bowtie2 as the aligner to align the mapped reads against the hg38 reference genome. Data quality control was performed using Picard (version 1.95) and bamtools to obtain general alignment statistics from the bam file. PREV12 was excluded due to low-quality issues.

### Gene expression data analyses

Gene expression values of PBMC RNA-seq data from 30 samples (15 men and 15 women) were converted into counts per million (CPM) using edgeR (version 3.32.1)^68^. Genes with CPM values less than 0.5 across all samples were filtered out. Differential gene expression analyses were conducted using generalized linear models with default trimmed mean of M values normalization. Significant genes were determined based on *P* values after Benjamini–Hochberg correction for multiple hypothesis testing (FDR *P* value of <0.05 and log_2_ (fold change) > ±0.585). The plasma cell activity score was calculated for each sample by computing the mean expression of genes within the plasma cell (M4.11) gene set^19^.

### Gene coexpression network analysis

Gene modules associated with vaccine responses were identified using WGCNA (v1.70.3)^69^. A signed coexpression network was constructed using baseline transcriptomes (*n* = 30; 15 men and 15 women) from both cohorts. Gene modules were identified using the blockwiseModules() function in the WGCNA R package (v4.0.4). Pearson correlation served as the correlation metric with a soft threshold of 18, deepSplit was set to 4, and mergeCutHeight was set to 0.25. Sizes of the modules were constrained to 50–2,000 genes. Each module was represented by its first principal component, known as the module eigen gene. Pearson correlations were computed to examine the correlation between modules’ eigen genes and metadata, such as PCV13 rank, PPSV23 rank, age, sex and T_H_1:T_H_17 ratio. Modules with a Pearson correlation of 0.5 and a *P* value of <0.05 were considered significant. For genes present in significant modules (midnight blue and dark green modules), gene set enrichment analysis was conducted using the hypergeometric test from the enrichR package in R^70^. KEGG, Reactome and BioPlanet were used as sources for annotations. Pathways with FDR-corrected *P* values of <0.05 were considered significant using Benjamini–Hochberg for multiple hypothesis test corrections.

### Sample processing and blood preparation for scRNA-seq

PBMCs were thawed quickly at 37 °C in DMEM supplemented with 10% fetal bovine serum. After thawing, cells were washed and suspended in PBS containing 0.04% bovine serum albumin. Cell viability was assessed using a Countess II automated cell counter (Thermo Fisher), and up to 12,000 cells (~4,000 cells from each hash-tagged sample) were loaded onto one lane of a 10x Genomics Chromium X. Single-cell capture, barcoding and library preparation were performed using the 10x Chromium platform version 3.1 chemistry following the manufacturer’s protocol (CG000388). cDNA and libraries were checked for quality using an Agilent 4200 Tapestation and Thermo Fisher Qubit Fluorometer, quantified by KAPA quantitative PCR and sequenced using 13% of an Illumina NovaSeq 6000 S4 v1.5 200-cycle flow cell lane with a 28-10-10-90 asymmetric read configuration, targeting 6,000 barcoded cells with a minimum sequencing depth of 50,000 read pairs per cell.

### Single-cell raw data processing

Illumina base call files for all libraries were converted to FASTQs using bcl2fastq v2.20.0.422 (Illumina) and aligned to the GRCh38 reference assembly with v32 annotations from GENCODE (10x Genomics GRCh38 reference 2020-A) using Cell Ranger multi pipeline v6.1.2 (10x Genomics). The generated gene–cell expression matrix was considered for downstream analysis.

### Single-cell gene expression analysis

Count matrices of PBMCs from four men and seven women (six PCV13 SRs and five PCV13 WRs) were obtained. The Scrublet^71^ package in Python (v3.8) was used to estimate the doublet score for each sample with an estimated doublet set to 0.6. Cells identified as doublets were removed. The following criteria were used for further cell exclusion: (1) cells with mitochondrial reads greater than 20% and (2) cells with fewer than 500 or more than 4,000 features (Supplementary Table 7). Filtered gene expression matrices were merged and processed using Seurat (v4.1.0). The total number of reads in each individual cell was normalized to CPM using the ‘NormalizeData’ function. The top 2,000 genes with the highest variance were selected using the ‘FindVariableFeatures’ function with the ‘vst’ method. Data were regressed against the percentage of mitochondrial genes and scaled to unit variance using the ‘ScaleData’ function. Principal-component analysis was performed using the ‘RunPCA’ function, followed by batch correction across samples using the ‘RunHarmony’ function from Harmony (v0.1.0). The first 50 principal components were used in the ‘FindNeighbors’ algorithm to construct the nearest neighbor graph. Next, the Louvain modularity optimization algorithm in the ‘FindClusters’ function was used to generate the clusters (resolution = 1.2). The ‘RunUMAP’ function was used to perform UMAP. Multiple rounds of marker identification, semisupervised cell-type annotation using established markers^72,73^, manual inspection and doublet removal were performed to create the final UMAP. Differentially expressed genes between clusters were identified using the ‘FindMarkers’ function in Seurat with the Wilcoxon rank-sum test.

Differences in cell frequency between PCV13 SRs and WRs were calculated by computing the frequencies of each immune cell type within PBMCs for each donor. A Wilcoxon rank-sum test was used to evaluate differences in cell proportions between SRs and WRs. Differentially expressed genes between PCV13 SRs and WRs for each cell type were identified from pseudobulked data using the edgeR package in R^68^. Genes with a log_2_ (fold change) greater than or less than ±0.25, with an FDR *P* value less than 0.05 and expressed in at least 10% of the cells in both SR and WR groups were considered significantly differentially expressed.

### CIBERSORTx analysis

The cellular frequencies of various immune cell subsets in the baseline bulk transcriptomes of PCV13, PPSV23 (from this study) and Fluzone^31^ data were quantified using CIBERSORTx^74^. A signature matrix from the PCV13 scRNA-seq data with cellular annotations was used to deconvolve the bulk transcriptomes (default parameters, permutation = 100).

### Fluzone response (GSE45735)

Influenza vaccine responses in healthy young adults (*n* = 5) were assessed using published RNA-seq data^75^. Normalized expression values (reads assigned per million mapped reads) for the samples at day 0 (baseline) and day 7 after vaccination were obtained from the Gene Expression Omnibus database. The fold difference in expression of constant heavy chain immunoglobulin genes was calculated by subtracting gene expression values at day 7 after vaccination from the baseline values. The mean fold differences in expression of these genes were then compared with those of the respective genes in our data (day 10 baseline).

### Fluzone response (GSE59654)

Microarray data (GSE59654)^31^ containing non-frail Fluzone responders (*n* = 5) and non-responders (*n* = 11) were used to assess the association between CD16^+^ NK cell frequency at baseline and Fluzone responsiveness. The data were retrieved, normalized, filtered for probes with a detection *P* value of <0.05 in over 20% of the samples and mapped to genes. Immune deconvolution was performed using CIBERSORTx^74^ with parameters specified in the CIBERSORTx analysis section. CD16^+^ NK cell frequency at baseline was compared between Fluzone responders and non-responders using a Wilcoxon rank-sum test.

### Fluad response (GSE211560)

The association between CD16^+^ NK cell frequency at baseline and Fluad responses in older adults^32^ was assessed by reanalyzing scRNA-seq data from Fluad responders (*n* = 3) and non-responders (*n* = 3) using the Seurat R package (v4.0.4). Baseline samples (responders, *n* = 3; non-responders, *n* = 3) were retrieved and preprocessed with filters described in the original study. Subclusters were annotated based on known markers, and immune cell frequencies in total PBMCs were computed. A Wilcoxon rank-sum test was applied to compare CD16^+^ NK cell frequencies between Fluad responders and non-responders at baseline.

### Statistical tests

Statistical analyses for bulk- and scRNA-seq data were performed as described above. *P* values for box plots were calculated using two-sided Wilcoxon rank-sum tests using the stat_compare_means function from the ggpubr package (v0.4.0). For scatter plots, Pearson’s correlation coefficients were reported, and *P* values were calculated using cor.test from the stats package (v4.0.4) at default settings. The sample size was not determined using a specific statistical test but was based on studies by us^19^ and others^32^. Data distribution was assumed to be normal, but this was not formally tested.

### Reporting summary

Further information on research design is available in the Nature Portfolio Reporting Summary linked to this article.

## Online content

Any methods, additional references, Nature Portfolio reporting summaries, source data, extended data, supplementary information, acknowledgements, peer review information; details of author contributions and competing interests; and statements of data and code availability are available at 10.1038/s41590-023-01717-5.

### Supplementary information

Reporting Summary

Peer Review File

Supplementary Table 1 Donor demographics. Donor-level details, including vaccine received, age (in years), sex, race, ethnicity, BMI, CMV seropositivity, frailty index and number of concomitant medications, are provided. Cohort-level demographic summaries are also available. *P* values were determined using a two-sided *t*-test for continuous variables, evaluating each donor parameter individually.

Supplementary Table 2 Functional antibody response to PCV13 and PPSV23. OPA titers at day −7 (baseline) and day 28 and the fold difference in their antibody titers (day 28/baseline) for PCV13 and PPSV23, respectively. Vaccine responsiveness scores, such as strength, extent, rank and maxRBA, which assess the response to PCV13 and PPSV23, are provided.

Supplementary Table 3 Gene expression changes in PBMCs after PCV13 and PPSV23 vaccination. Gene expression changes at days 1, 10 and 60 after PCV13 or PPSV23 vaccination compared to baseline. Genes with a log_2_ (fold change) of >±0.585 (that is, 1.5) and FDR *P* values of <0.05 were considered to be significantly differentially expressed. The data were analyzed using a two-sided likelihood ratio test. Information on the proteins encoded by differentially expressed genes on day 10 compared to baseline for PCV13 and PPSV23.

Supplementary Table 4 Longitudinal profiling of DC, B cell and CD4^+^ T cell subsets in PCV13 and PPSV23 donors. Antibody catalog information. Absolute numbers of immune cell subsets at baseline and days 1, 10 and 60. The markers used for profiling these immune subsets are provided in the table.

Supplementary Table 5 Gene coexpression-based modules inferred using WGCNA. The table presents the 33 modules identified by WGCNA, along with their correlation with metadata such as PCV13 rank, PPSV23 rank, sex, age and T_H_1:T_H_17 ratio. Correlation analysis was performed for PCV13 and PPSV23 donors separately using Pearson correlation, and *P* values were determined by *t*-test (two sided).

Supplementary Table 6 PCV13-associated midnight blue (that is, CYTOX) and PPSV23-associated dark green modules. Genes and pathways enriched in midnight blue and dark green modules. The midnight blue module is significantly and negatively correlated to PCV13, while the dark green module is significantly and negatively correlated to PPSV23. Correlation analysis was performed using the Pearson correlation metric, and *P* values were determined by *t*-test (two sided).

Supplementary Table 7 Number of cells before and after preprocessing filters across 11 PCV13 donors (6 SRs and 5 WRs) at baseline. Preprocessing filters include doublet removal and other steps described in the Methods.

Supplementary Table 8 Differentially expressed genes in CD56^dim^CD16^+^ NK cells. Genes with log_2_ (fold change) values of >±0.25, FDR *P* values of <0.05 and expressed in at least 10% of the cells in both groups (SRs and WRs) were considered to be significantly differentially expressed. The data were analyzed using a two-sided likelihood ratio test.

Supplementary Table 9 Non-informative donors in the PCV13 and PPSV23 cohorts. Identification of potential non-informative donors in both PCV13 and PPSV23 cohorts using a data-driven threshold. The effect of age, sex, T_H_1 and T_H_17 abundance, module scores and CD16^+^ NK cells on vaccine responses to PCV13 and PPSV23 following the exclusion of non-informative donors determined by two different cutoffs. Correlation analysis was performed using the Pearson correlation metric, and *P* values were determined by *t*-test (two sided).

### Source data

Source Data Fig. 1 Statistical source data.

Source Data Fig. 2 Statistical source data.

Source Data Fig. 3 Statistical source data.

Source Data Fig. 4 Statistical source data.

Source Data Fig. 5 Statistical source data.

Source Data Fig. 6 Statistical source data.

Source Data Extended Data Fig. 1 Statistical source data.

Source Data Extended Data Fig. 2 Statistical source data.

Source Data Extended Data Fig. 3 Statistical source data.

Source Data Extended Data Fig. 4 Statistical source data.

Source Data Extended Data Fig. 5 Statistical source data.

Source Data Extended Data Fig. 6 Statistical source data.

Source Data Extended Data Fig. 8 Statistical source data.

Source Data Extended Data Fig. 9 Statistical source data.

Source Data Extended Data Fig. 10 Statistical source data.

## Data Availability

Raw files of the bulk- and scRNA-seq data generated in this study are available in dbGAP under the accession code phs002361.v2.p1. The processed bulk and single-cell transcriptome data have been deposited in NCBI Gene Expression Omnibus under the accession numbers GSE247276 and GSE247277. Source data are provided with this paper.

## Code availability

All the custom code used to analyze the data has been deposited in GitHub and can be accessed using the following link: https://github.com/sathyabaarathisr/pneumococcal_vaccine_BIS.
