# Supplementary material for: Distinct baseline immune characteristics associated with responses to conjugated and unconjugated pneumococcal polysaccharide vaccines in older adults
Source: Nat Immunol. 2024 Jan 5;25(2):316–29. doi: 10.1038/s41590-023-01717-5 (PMC10834365; doi:10.1038/s41590-023-01717-5)
Supplement: Supplementary file 1 — Reporting Summary [file 41590_2023_1717_MOESM1_ESM.pdf]

nature portfolio

Corresponding author(s): Duygu Ucar (duygu.ucar@jax.org)

Last updated by author(s): 11/14/2023

Reporting Summary

Nature Portfolio wishes to improve the reproducibility of the work that we publish. This form provides structure for consistency and transparency in reporting. For further information on Nature Portfolio policies, see our Editorial Policies and the Editorial Policy Checklist.

Statistics

For all statistical analyses, confirm that the following items are present in the figure legend, table legend, main text, or Methods section.

n/a	Confirmed	
☐	☒	The exact sample size (*n*) for each experimental group/condition, given as a discrete number and unit of measurement
☐	☒	A statement on whether measurements were taken from distinct samples or whether the same sample was measured repeatedly
☐	☒	The statistical test(s) used AND whether they are one- or two-sided<br>*Only common tests should be described solely by name; describe more complex techniques in the Methods section.*
☐	☒	A description of all covariates tested
☐	☒	A description of any assumptions or corrections, such as tests of normality and adjustment for multiple comparisons
☐	☒	A full description of the statistical parameters including central tendency (e.g. means) or other basic estimates (e.g. regression coefficient) AND variation (e.g. standard deviation) or associated estimates of uncertainty (e.g. confidence intervals)
☐	☒	For null hypothesis testing, the test statistic (e.g. *F*, *t*, *r*) with confidence intervals, effect sizes, degrees of freedom and *P* value noted<br>*Give P values as exact values whenever suitable.*
☒	☐	For Bayesian analysis, information on the choice of priors and Markov chain Monte Carlo settings
☒	☐	For hierarchical and complex designs, identification of the appropriate level for tests and full reporting of outcomes
☐	☒	Estimates of effect sizes (e.g. Cohen's *d*, Pearson's *r*), indicating how they were calculated

Our web collection on statistics for biologists contains articles on many of the points above.

Software and code

Policy information about availability of computer code

Data collection	Flow cytometry data was generated using BD LSR Fortessa X-20. Bulk RNA Sequencing data was generated using Illumina NovaSeq (2x150 bp paired end reads) platform. Single cell RNA sequencing was generated using the 10x Chromium platform version 3.1 chemistry platform.
Data analysis	FlowJo V9.9.6 software (Treestar Inc); Trimmomatic (v0.33); Picard (v1.95); Cell Ranger multi pipeline v6.1.2; CIBERSORTx; RStudio v3.28.1; Python 3.8; Scrublet (v3.8); WGCNA (v1.70.3); edgeR (version 3.32.1); Seurat (v4.1.0); ggpubr (v0.4.0); Harmony (v0.1.0).<br><br>The scripts used for the data processing and analysis are available here : https://github.com/sathyabaarathisr/pneumococcal_vaccine_BIS/

For manuscripts utilizing custom algorithms or software that are central to the research but not yet described in published literature, software must be made available to editors and reviewers. We strongly encourage code deposition in a community repository (e.g. GitHub). See the Nature Portfolio guidelines for submitting code & software for further information.

Data

Policy information about availability of data

All manuscripts must include a data availability statement. This statement should provide the following information, where applicable:

- Accession codes, unique identifiers, or web links for publicly available datasets
- A description of any restrictions on data availability
- For clinical datasets or third party data, please ensure that the statement adheres to our policy

Raw files of the bulk and single cell RNAseq data generated in this study is available in the dbGAP under the accession code phs002361.v2.p1. The processed bulk and single-cell transcriptome data has been deposited in NCBI GEO under the accession numbers GSE247276 and GSE247277.
GEO IDs of previously published datasets used in this study: GSE45735, GSE59654 and GSE211560.

Research involving human participants, their data, or biological material

Policy information about studies with human participants or human data. See also policy information about sex, gender (identity/presentation), and sexual orientation and race, ethnicity and racism.

Reporting on sex and gender	We studied sex differences in our data and report the differences observed between sexes.
Reporting on race, ethnicity, or other socially relevant groupings	Race and ethnicity information is provided in Supplementary Table S1.
Population characteristics	The population characteristics and demographics are provided in Supplementary Table s1.
Recruitment	All the participants in the study were recruited by the UConn Center on Aging Recruitment and Community Outreach Research Core (http://health.uconn.edu/aging/research/research-cores/). There was no self-selection conflict involved.
Ethics oversight	Study was conducted following approval by the UConn Health Center Institutional Review Board (IRB Number: 16-071J-1) and registration on ClinicalTrials.gov (NCT03104075). All participants provided informed consent and were compensated for their time and study visits.

Note that full information on the approval of the study protocol must also be provided in the manuscript.

Field-specific reporting

Please select the one below that is the best fit for your research. If you are not sure, read the appropriate sections before making your selection.

☒ Life sciences ☐ Behavioural & social sciences ☐ Ecological, evolutionary & environmental sciences

For a reference copy of the document with all sections, see nature.com/documents/nr-reporting-summary-flat.pdf

Life sciences study design

All studies must disclose on these points even when the disclosure is negative.

Sample size	PCV13 cohort: 10 men and 9 women.<br>PPSV23 cohort: 10 men and 10 women.<br>OPA assay was performed for all 39 participants (20 men and 19 women) at baseline and day 28 post vaccination.<br>Flow cytometric analysis was performed for 35 participants (19 men and 16 women) at baseline, day 1, day 10, day 28 and day 60.<br>Bulk RNAseq was performed for 14 (7 men and 7 women) participants from the PCV13 cohort and 16 (8 men and 8 women) participants from the PPSV23 cohort at four different timepoints (baseline, day 1, day 10, day 28).<br>scRNAseq was performed for 4 men and 7 women from PCV13 cohort at baseline. No statistical test was used to determine the number of samples. However, the sample size used was determined as appropriate to detect the effect of vaccine response based on our previous studies and those conducted by others.<br><br>Riese, P. et al. Distinct immunological and molecular signatures underpinning influenza vaccine responsiveness in the elderly. Nat. Commun. 13, 6894 (2022).<br>Obermoser, G. et al. Systems Scale Interactive Exploration Reveals Quantitative and Qualitative Differences in Response to Influenza and Pneumococcal Vaccines. Immunity 38, 831–844 (2013).
Data exclusions	No data was excluded from our analysis after quality check.
Replication	All the experiments (funtional antibody titer estimation using OPA, flow cytometry, bulk and single cell RNAseq profiling) were performed once in multiple biological replicates, and the analysis showed good correlation between the biological replicates.<br>Flow cytometry assays were performed on fresh blood collected from 39 donors over multiple visits pre-and post-vaccination. Cell staining for

different cell compartments was successfully replicated in multiple donors at the same visit time point throughout the vaccine study.

Randomization	Among the recruited older adults, they were randomly assigned to each vaccine arm while trying to balance for biological sex and age and frailty distribution in each arm.
Blinding	This is an open label study and not blinded.

Reporting for specific materials, systems and methods

We require information from authors about some types of materials, experimental systems and methods used in many studies. Here, indicate whether each material, system or method listed is relevant to your study. If you are not sure if a list item applies to your research, read the appropriate section before selecting a response.

Materials & experimental systems

n/a	Involved in the study
☐	☒ Antibodies
☒	☐ Eukaryotic cell lines
☒	☐ Palaeontology and archaeology
☒	☐ Animals and other organisms
☐	☒ Clinical data
☒	☐ Dual use research of concern
☒	☐ Plants

Methods

n/a	Involved in the study
☒	☐ ChIP-seq
☐	☒ Flow cytometry
☒	☐ MRI-based neuroimaging

Antibodies

Antibodies used

Marker; Clone; Label; Antibody Registry ID;
Lin1 (CD3,14,16,19,20,56); CD3(SK7), CD16(3G8), CD19(SJ25C1), CD20(L27),CD14(MφP9), CD56(NCAM16.2); FITC; BD Biosciences Cat# 340546, RRID:AB_400053;
CD138; MI15; PerCP-Cy5.5; BD Biosciences Cat# 341097,RRID:AB_400219;
IgD; IA6-2; FITC; BD Biosciences Cat# 555778, RRID:AB_396113;
PD-1 (CD279); MIH4; FITC; BD Biosciences Cat# 557860, RRID:AB_2159176;
CD3; UCHT1; AF700; BD Biosciences Cat# 557943, RRID:AB_396952;
CCR6 (CD196); 11A9; PE; BD Biosciences Cat# 559562, RRID:AB_397273;
CD11c; B-ly6; V450; BD Biosciences Cat# 560369, RRID:AB_1645557;
CD40; 5C3; APC-R700; BD Biosciences Cat# 565179, RRID:AB_2739094;
CD19; J4-119; ECD; Beckman Coulter Cat# IM2708U, RRID:AB_130854;
CD45RA; 2H4; ECD; Beckman Coulter Cat# IM2711U, RRID:AB_10640553;
CD20; 2H7; APC; BioLegend Cat# 302310, RRID:AB_314258;
ICOS (CD278); C398.4A; PE-Cy7; BioLegend Cat# 313520, RRID:AB_10643411;
CD4; OKT4; APC-Cy7; BioLegend Cat# 317418, RRID:AB_571947;
BDCA-1 (CD1c); L161; PerCP-Cy5.5; BioLegend Cat# 331514, RRID:AB_1227535;
CXCR3 (CD183); G025H7; BV421; BioLegend Cat# 353716, RRID:AB_2561448;
CD27; M-T271; PE; BioLegend Cat# 356406, RRID:AB_2561825;
CD86; IT2.2; PE-Cy7; BioLegend Cat# 305422, RRID:AB_2074981;
CXCR5 (CD185); J252D4; APC; BioLegend Cat# 356908, RRID:AB_2561817;
BDCA-2 (CD303); AC144 PE; Miltenyi; Biotec Cat# 130-090-511, RRID:AB_244168;
BDCA-3 (CD141); AD5-14H12; APC; Miltenyi; Biotec Cat# 130-090-907, RRID:AB_244170;
HLA-DR; LN3; APC-eFluor 780; Thermo Fisher Scientific Cat# 47-9956-42, RRID:AB_1963603;

Validation

All antibodies used were evaluated by the manufacturers as provided in their websites. Refer the link under reference link for the references associated to marker validation.

Marker; Reference
Lin1 (CD3,14,16,19,20,56); https://www.bdbiosciences.com/en-us/products/reagents/flow-cytometry-reagents/clinical-discovery-research/multicolor-cocktails-and-kits-ruo-gmp/anti-human-lineage-cocktail-1-lin-1-cd3-cd14-cd16-cd19-cd20-cd56.340546

CD138; https://www.bdbiosciences.com/en-us/products/reagents/flow-cytometry-reagents/clinical-diagnostics/single-color-antibodies-asr-ivd-ce-ivd/cd138-percp-cy-5-5.341097

IgD; https://www.bdbiosciences.com/en-us/products/reagents/flow-cytometry-reagents/research-reagents/single-color-antibodies-ruo/fitc-mouse-anti-human-igd.562023
PD-1 (CD279); https://www.bdbiosciences.com/en-us/products/reagents/flow-cytometry-reagents/research-reagents/single-color-antibodies-ruo/fitc-mouse-anti-human-cd279.557860

CD3; https://www.bdbiosciences.com/en-us/products/reagents/flow-cytometry-reagents/research-reagents/single-color-antibodies-ruo/alexa-fluor-700-mouse-anti-human-cd3.561027

CCR6 (CD196); https://www.bdbiosciences.com/en-us/products/reagents/flow-cytometry-reagents/research-reagents/single-color-

antibodies-ruo/pe-mouse-anti-human-cd196-ccr6.559562

CD11c; https://www.bdbiosciences.com/en-us/products/reagents/flow-cytometry-reagents/research-reagents/single-color-antibodies-ruo/v450-mouse-anti-human-cd11c.560369

CD40; https://www.bdbiosciences.com/en-us/products/reagents/flow-cytometry-reagents/research-reagents/single-color-antibodies-ruo/apc-r700-mouse-anti-human-cd40.565179

CD19; https://elifesciences.org/articles/60939/figures, https://www.ncbi.nlm.nih.gov/pmc/articles/PMC7873835/, https://pubmed.ncbi.nlm.nih.gov/32661393/

CD45RA; https://www.ncbi.nlm.nih.gov/pmc/articles/PMC6076291/, https://www.nature.com/articles/s41467-022-32491-x, https://www.nature.com/articles/s41467-022-32491-x

CD20; https://www.biolegend.com/en-us/products/apc-anti-human-cd20-antibody-557

CD38; https://www.biolegend.com/en-us/products/brilliant-violet-421-anti-human-cd38-antibody-7145

ICOS (CD278); https://www.biolegend.com/en-us/products/pe-cyanine7-anti-human-mouse-rat-cd278-icos-antibody-6908

CD4; https://www.biolegend.com/en-us/products/apc-cyanine7-anti-human-cd4-antibody-3658

BDCA-1 (CD1c); https://www.biolegend.com/en-us/products/percp-cyanine5-5-anti-human-cd1c-antibody-5182

CXCR3 (CD183); https://www.biolegend.com/en-us/products/brilliant-violet-421-anti-human-cd183-cxcr3-antibody-7712

CD27; https://www.biolegend.com/en-us/products/pe-anti-human-cd27-antibody-8371

CD86; https://www.biolegend.com/en-us/products/pe-cyanine7-anti-human-cd86-antibody-3961

CXCR5 (CD185); https://www.biolegend.com/en-us/products/apc-anti-human-cd185-cxcr5-antibody-8360

BDCA-2 (CD303); https://www.miltenyibiotec.com/US-en/products/cd303-bdca-2-antibody-anti-human-ac144.html#conjugate=pe:size=100-tests-in-200-ul

BDCA-3 (CD141); https://www.miltenyibiotec.com/US-en/products/cd141-bdca-3-antibody-anti-human-ad5-14h12.html#conjugate=apc:size=100-tests-in-200-ul

HLA-DR; https://www.thermofisher.com/antibody/product/HLA-DR-Antibody-clone-LN3-Monoclonal/47-9956-42

Clinical data

Policy information about clinical studies

All manuscripts should comply with the ICMJE guidelines for publication of clinical research and a completed CONSORT checklist must be included with all submissions.

Clinical trial registration

NCT03104075

Study protocol

The full study protocol can accessed here: https://clinicaltrials.gov/study/NCT03104075

Data collection

Blood samples were obtained from 39 healthy volunteers residing in the Greater Hartford, CT, USA region recruited by the UConn Center on Aging Recruitment and Community Outreach Research Core (http://health.uconn.edu/aging/research/research-cores/). Volunteers were vaccinated from May to early fall in 2017-2018 to avoid potential overlap with seasonal peak periods of influenza vaccination and infection.

Outcomes

Primary outcomes of the study:
1. Pneumococcal-specific Antibody Responses
To vaccinate healthy older participants with pneumococcal vaccines, collect longitudinal blood samples and assess pneumococcal-specific antibody responses. The unit of measurement used is log2 titer defined as a measure to quantify the overall strength of responses using the sum of all serotype responses. Data shown below is from longitudinal study timepoints (pre and post) first pneumococcal vaccine. At study endpoint, Visit 7, a second pneumococcal vaccine was administered and no further sample collection visits were conducted as proposed in the study design.

2. Pneumococcal-specific Antibody Responses - Fold Change Post First Vaccination
Fold change between the baseline and post first vaccination titers was calculated for each pneumococcal vaccine cohort.

Secondary outcomes of the study:
1. Number of Genes Upregulated Following Vaccination With PCV13 or PPSV23
RNA-seq and ATAC-seq to enable quantitative assessment of both coding RNA's and ncsRNA's as well as to resolve the epigenetic landscape of immune cells in the context of vaccine responses. We assessed the number of genes upregulated post first vaccination [PCV13 or PPSV23].

2.Alterations to APCs, Tfh Cells or B Cells in Response to PCV13 and PPSV23
Measure of functional status of immune cells in older participants following administration of a single pneumococcal vaccine,

Prevnar13 or Pneumovax23, at baseline and 10 days post first vaccination.

Flow Cytometry

Plots

Confirm that:

☒ The axis labels state the marker and fluorochrome used (e.g. CD4-FITC).

☒ The axis scales are clearly visible. Include numbers along axes only for bottom left plot of group (a 'group' is an analysis of identical markers).

☒ All plots are contour plots with outliers or pseudocolor plots.

☒ A numerical value for number of cells or percentage (with statistics) is provided.

Methodology

Sample preparation	Fluorescent-labeled antibody cocktails for different cell-surface staining panels were pre-mixed in BD Horizon Brilliant Stain Buffer (BD Biosciences) 10 minutes before staining. Antibody cocktails were added over 100 μL aliquots of anticoagulated whole blood in a 5 ml FACS tube within 60 minutes of blood collection. Samples were incubated for 15 minutes at room temperature then lysed and fixed with 2 ml of 1x FACS lysing solution (BD Biosciences) for 8 minutes at room temperature. The lysed samples were washed twice to remove the unbound antibodies, lysed RBCs, and platelets and finally resuspended in 250 μL of PBS to which 50 μL of count beads suspension (Count Bright Absolute Counting Beads, Thermo Fisher) were added for the detection of absolute cell counts.
Instrument	BD LSR Fortessa X-20
Software	Data were acquired using BD FACS Diva and the analyzed using FlowJo V9.9.6 software.
Cell population abundance	No cell sorting procedure was used in the study.
Gating strategy	1. CD4+ T cell compartment<br><br>a. Bulk blood cells via FSC-A vs SSC-A.<br>b. Doublets exclusion via FSC-H vs FSC-A.<br>c. CD4 gating via CD3 vs CD4 of the gated Singlets<br>d. Memory CD4 via CD4 vs CD45RA of the gated CD4<br>e. Th and Tfh via CD4 vs CXCR5 of the gated Memory CD4<br>f. Th1/Th2/Th17/Th1-Th17 via CXCR3 vs CCR6 of the gated Th<br>g. Tfh1/Tfh2/Tfh17/Tfh1-Tfh17 via CXCR3 vs CCR6 of the gated Tfh<br><br>2. B cell compartment<br><br>a. Bulk blood cells via FSC-A vs SSC-A.<br>b. Doublets exclusion via FSC-H vs FSC-A.<br>c. CD19 gating via CD3 vs CD19 of the gated Singlets<br>d. Memory CD19 via IgD vs CD27 of the gated CD19<br>e. CD20(-) Memory CD19 via CD19 vs CD20 of the gated Memory CD19<br>f. Plasmablast/Plasmacell via CD38 vs CD138 of the gated CD20(-) Memory B cells<br><br>3. DC compartment<br><br>a. Bulk blood cells via FSC-A vs SSC-A.<br>b. Doublets exclusion via FSC-H vs FSC-A.<br>c. HLA-DR(+)Lineage(-) via HLA-DR vs Lin of the gated Singlets<br>d. CD11c(+) and BDCA2(+) via CD11c vs CD303 of the gated HLA-DR(+)Lineage(-)<br>e. cDC1 and cDC2 via CD1c vs CD141 of the gated Memory CD11c(+).

☒ Tick this box to confirm that a figure exemplifying the gating strategy is provided in the Supplementary Information.
