# Supplementary material for: Distinct baseline immune characteristics associated with responses to conjugated and unconjugated pneumococcal polysaccharide vaccines in older adults
Source: Nat Immunol. 2024 Jan 5;25(2):316–29. doi: 10.1038/s41590-023-01717-5 (PMC10834365; doi:10.1038/s41590-023-01717-5)
Supplement: Supplementary file 2 — Peer Review File [file 41590_2023_1717_MOESM2_ESM.pdf]

Peer Review Information

Journal: Nature Immunology
Manuscript Title: Distinct baseline immune characteristics associated with responses to conjugated and unconjugated pneumococcal polysaccharide vaccines in older adults
Corresponding author name(s): Dr Duygu Ucar

Editorial Notes:

**Transferred manuscripts**	This manuscript has been previously reviewed at another journal that is not operating a transparent peer review scheme. This document only contains reviewer comments, rebuttal and decision letters for versions considered at Nature Immunology

Reviewer Comments & Decisions:

Decision Letter, initial version:

8th Jun 2023

Dear Duygu,

Thank you for providing a point-by-point response to the referees' concerns on your manuscript entitled, "Distinct baseline immune characteristics associated with responses to conjugated and unconjugated pneumococcal polysaccharide vaccines in older adults". As noted previously, while they find your work of interest, some important points are raised. We are very interested in the possibility of publishing your study in Nature Immunology. It appears from your response letter that many of the referees' concerns can be addressed by textual clarifications as well as articulating the limitations of the current study. I will have to look into whether you can provide the exact donor ages or if a range is preferred for the donor cohorts.

We therefore invite you to revise your manuscript taking into account all reviewer comments along the lines proposed in your response letter. Please highlight all changes in the manuscript text file in Microsoft Word format.

We are committed to providing a fair and constructive peer-review process. Do not hesitate to contact us if there are specific requests from the reviewers that you believe are technically impossible or unlikely to yield a meaningful outcome.

When revising your manuscript:

* Include a "Response to referees" document detailing, point-by-point, how you addressed each referee comment. If no action was taken to address a point, you must provide a compelling argument. This response will be sent back to the referees along with the revised manuscript.

* If you have not done so already please begin to revise your manuscript so that it conforms to our Article format instructions at http://www.nature.com/ni/authors/index.html. Refer also to any guidelines provided in this letter.

* Please include a revised version of any required reporting checklist. It will be available to referees to aid in their evaluation of the manuscript goes back for peer review. They are available here:

Reporting summary:
https://www.nature.com/documents/nr-reporting-summary.pdf

When submitting the revised version of your manuscript, please pay close attention to our href="https://www.nature.com/nature-portfolio/editorial-policies/image-integrity">Digital Image Integrity Guidelines. and to the following points below:

-- that unprocessed scans are clearly labelled and match the gels and western blots presented in figures.
-- that control panels for gels and western blots are appropriately described as loading on sample processing controls
-- all images in the paper are checked for duplication of panels and for splicing of gel lanes.

Finally, please ensure that you retain unprocessed data and metadata files after publication, ideally archiving data in perpetuity, as these may be requested during the peer review and production process or after publication if any issues arise.

Please use the link below to submit your revised manuscript and related files:

[REDACTED]

We hope to receive your revised manuscript within six weeks. If you cannot send it within this time, please let us know. We will be happy to consider your revision so long as nothing similar has been accepted for publication at Nature Immunology or published elsewhere.

Please do not hesitate to contact me if you have any questions or would like to discuss these revisions further.

Nature Immunology is committed to improving transparency in authorship. As part of our efforts in this direction, we are now requesting that all authors identified as 'corresponding author' on published papers create and link their Open Researcher and Contributor Identifier (ORCID) with their account on the Manuscript Tracking System (MTS), prior to acceptance. ORCID helps the scientific community achieve unambiguous attribution of all scholarly contributions. You can create and link your ORCID from the home page of the MTS by clicking on 'Modify my Springer Nature account'. For more information please visit please visit www.springernature.com/orcid.

We look forward to seeing the revised manuscript and thank you for the opportunity to review your work.

Kind regards,

Laurie

Laurie A. Dempsey, Ph.D.
Senior Editor
Nature Immunology
l.dempsey@us.nature.com
ORCID: 0000-0002-3304-796X

Referee expertise:

Referee #1: human vaccine responses

Referee #2: human immunology & infectious disease

Referee #3: bacterial/pneumoncoccal infections

Reviewers' Comments:

Reviewer #1:
Remarks to the Author:
This paper describes a small clinical study where people > 60 years old were immunized with the polysaccharide or conjugate pneumococcal vaccine.

The data confirmed both the pneumococcal plain polysaccharide-based vaccine PPSV23, and a protein conjugate based vaccine PCV13 induced a strong antibody response at day 28.

Transcriptional responses analyzed at day 0 and 60 showed no difference, while those taken at day 10 showed a strong thermoblast and responses in both groups. Interestingly, baseline flow cytometry and RNA-seq data (bulk and single cell) revealed a novel baseline phenotype that is specifically associated with weaker PCV13 responses, characterized by i) increased expression of cytotoxicity-associated genes and increased CD16+ NK frequency; ii) increased Th17 and decreased Th1 cell frequency. Men were more likely to display this cytotoxic phenotype and mounted weaker responses to PCV13 than women. Baseline expression levels of a distinct gene set was predictive of PPSV23 responses.

This work has been well designed and conducted. The results support the study conclusions.

Comments

1.This work has been well designed and conducted. The results support the study conclusions. The number of subjects is small, but the evidence that baseline Th1/Th17/CD16 NK frequencies impact vaccine response is of interest in the context of vaccinating the elderly.
2.Day 10 is probably not the best time to see the transcriptional response. Indeed, the transcriptional activities are expected in the first few doses after vaccination.
3.With weaker PCV13 responses, increased Th17 and decreased Th1 cell frequency have been revealed. A different behavior might be expected in a different target population (e.g., infants post multiple injections)? Does it confirm previous studied (see Reference 52 and 53)?
4.The authors concluded these findings might also give rise to novel intervention strategies to change the baseline immune phenotypes to alter the outcome of vaccine responses, e.g., by temporarily blocking the immunosuppressive or cytolytic functions of CD16+ NK cells prior to vaccination. Can the authors expand this option more in details?
5.Can the Author comment on baseline characteristics of patient population and whether concomitant medication might influence the different observed immune responses? (I don’t have access to supplementary table 1).

6.Have the Authors investigated whether the reported differences in baseline T cell activation are impacting memory B cell responses to PCV vaccination at latest time point?

Minor
1.Line 70. "In the US, the current recommendation for adults aged ≥ 65 years is to give a conjugated vaccine (PCV15/PCV20) followed by PPSV23 at least one year later". If PCV20 is used, a dose of PPSV23 is not indicated.

Reviewer #2:
Remarks to the Author:
The authors combine serology, phagocytosis assays (OPA), bulk and single cell RNAseq and cutoff to present a detailed evaluation of factors that influence antibody responses to the conjugated (PCV) and unconjugated (PPV) pneumococcal vaccines. Their data indicates that poor response to PCV correlated with higher age (expected), perhaps male sex, and (the novel findings) high baseline Th17 and CD16+ NK cell numbers/proportions and a corresponding RNA signature for cytotoxicity genes. The data are all presented and very extensive, and I found the manuscript of significant interest.

Major issues.

There are two really significant issues with the data interpretation:

1. Although they present males as having worse responses than females this different was not statistically significant (Figs 1F) although very very close. This makes all the male/female comparisons further on tricky to fully support. For the male male data has this been adjusted for age? Or at least what is the median age and range for males v females?

2. There are multiple places in the figures where correlations are shown for PCV to immune data that are shown not to be present for PPV. However, the data shown in figure 2A suggests there is a significant plasma blast signature for many of the PPV non-responders; the authors comment on this and suggest this represents antibody responses to serotypes that they have not analysed in their serology data, and this is probably true.. However, if so that completely invalidates the ranking for the PPV recipients (not for the PCV recipients) and means the differences in the immune factors correlated to good/bad response in PCV versus PPV recipients are also not really valid. The PPV data can be used as a sort of negative control but I don't think can be interpreted was showing PPV poor responders do not have the same immune profile as PCV poor responders. This affects multiple aspects of the text and the figures. They could do the serology for the 10 or so missing serotypes for the PPV recipients; but the

number of responders is high so may not be enough differential for all the correlations to have adequate power.

A few less major issues:
1. Can the authors make it clearer how much of the CYTOX signatures associated with poor response from bulk RNAseq is shown by the scRNAseq to originate from the CD16+ NK cells rather than Th17 etc
2. Some data has negatively correlated NK cell numbers with Th17 CD4s; can the authors discuss more whether these are two independent markers of poor response or could be functional related and what mechanisms might be involved?
3. the Th1/17 ratio and age correlation needs to be in figure 3 (and maybe some of the other data eg NCAM1 expression)
4. One for the statistical editor; are they happy adequate correction for multiple comparisons has been specifically for correlations between datasets?

Minor issues:
lines 67 – 68 decline with age – this is not unexpected now given the data we have for all other vaccines

lines 69-70 not for improving immunogenicity but for extending range of serotypes covered

lines 110 to 114 could be merged (‘no differences between vaccine group or males versus females for….’)

fig 1b and c why is age a range?

so is rank purely decided on by the OPA data? that is fine but for clarity this needs to be a bit more explicit perhaps

interesting how many subjects have really poor responses even to PCV - is this due to high baseline antibody to those serotypes which is perhaps unlikely but needs checking

line 321 this text is too early as pre-gene signature asociations: 'Together these data establish that the increased frequency of CD16+ NK cells bear the CYTOX signature that is associated with reduced PCV13 responses'

fig 2A and suppl fig 3A some sort of trasnlation of genes for what they encode would be useful

supple fig 6b symbols covered over by p value text

line 376 - should be S. pneumoniae

Reviewer #3:
Remarks to the Author:
General Comments

At first, I read this paper finding it incremental and confirmatory either of past results or assumptions. But as I read in more detail, I found many gems hidden in the data and in the text. The study is excellent and important, but the write-up needs to be expanded and clarified to draw proper attention to the importance of the work.

Specific Comments

Title: the very important implication of the data that is missing from the title is that men and women respond differently to vaccine. This should be included in the title and abstract.

Abstract; Needs to draw attention to better PCV13 response in women than men, and underlying mechanisms.

Intro: I found the repeated reference to T-independent responses to polysaccharide somewhat irritating. Certainly, this is an immunological definition, derived from animal models, but it is not a complete description of the response of healthy adult humans to polysaccharide vaccine. It is even mis-leading as the data in the Results and the subsequent Discussion acknowledge. A proper introduction as to how PCV and PPV are handled in adults, to include discussion of marginal zone and follicular function in spleen, is an essential part of this paper that is missing.

Results: The results are very impressive in their rigour and careful analysis. Really detailed descriptions of both B and T cell function are presented. The immunoglobulin, OPA and plasmablast data might be somewhat incremental on what has been published before, but the RNA work and the T cell descriptions are novel, interesting and of translational importance.

Discussion: I would like to see a more comprehensive discussion. It is well known and not cited that PPV causes an excess of pneumonia (but not death) in vaccinated elderly adults, probably by immunoglobulin and maybe by B cell depletion. This should be reviewed and discussed. The APC and T-cell role in both PCV and PPV response should be compared. This will allow a proper appreciation of the paper. The suggestion of personalised vaccination should be more fully expanded. Would simple differentiation by gender be enough, or are there additional (simple, available) tests that could pick out the Th1/Th17 dependent functions described?

Journal requested comments

Summary of the key results
Older men and women received either PPV or PCV and subsequent immune responses were described in detail. IgG to polysaccharides, OPA data, plasmablast frequency and function as well as T-cell phenotype and function are described in a uniquely comprehensive study.

Originality and significance: if not novel, please include reference
The originality is not in the question that is addressed in this study, but in the extremely detailed manner in which it is answered. The significance of the paper is a profound translational suggestion that personalised vaccination may be considerably more effective than the current strategy.

Data & methodology: validity of approach, quality of data, quality of presentation
This study succeeds in being as completely thorough in addressing the question as modern methods will allow. The data are very, very extensive and the methodology cutting-edge. The data are presented in a very clear and comprehensive manner.

Appropriate use of statistics and treatment of uncertainties
These seem robust to me, but I would defer to more expert opinion.

Conclusions: robustness, validity, reliability
This is a state-of-the-art paper.
Suggested improvements: experiments, data for possible revision
I have put some suggestions about emphasis, implications and translation in the text; no additions to the experiments or analysis.

References: appropriate credit to previous work?
I've mentioned the concerns about PPV (not mentioned), the presentation of known immunology (impaired responses to PPV, T cell role in "T-independent" responses).

Clarity and context: lucidity of abstract/summary, appropriateness of abstract, introduction and conclusions
(these comments already above).

Author Rebuttal to Initial comments

Point-by-point response to reviewers' concerns

Reviewer #1:
Remarks to the Author:

This paper describes a small clinical study where people > 60 years old were immunized with the polysaccharide or conjugate pneumococcal vaccine. The data confirmed both the pneumococcal plain polysaccharide-based vaccine PPSV23, and a protein conjugate-based vaccine PCV13 induced a strong antibody response at day 28.

Transcriptional responses analyzed at day 0 and 60 showed no difference, while those taken at day 10 showed a strong thermoblast and responses in both groups. Interestingly, baseline flow cytometry and RNA-seq data (bulk and single cell) revealed a novel baseline phenotype that is specifically associated with weaker PCV13 responses, characterized by i) increased expression of cytotoxicity-associated genes and increased CD16+ NK frequency; ii) increased Th17 and decreased Th1 cell frequency. Men were more likely to display this cytotoxic phenotype and mounted weaker responses to PCV13 than women. Baseline expression levels of a distinct gene set was predictive of PPSV23 responses.

This work has been well designed and conducted. The results support the study conclusions.

Comments

1. This work has been well designed and conducted. The results support the study conclusions. The number of subjects is small, but the evidence that baseline Th1/Th17/CD16 NK frequencies impact vaccine response is of interest in the context of vaccinating the elderly.

Response: We thank the reviewer for appreciating the unique design and the novel clinically relevant findings of our study.

2. Day 10 is probably not the best time to see the transcriptional response. Indeed, the transcriptional activities are expected in the first few doses after vaccination.

Response: We agree, it is possible that other time points could have provided more information about the transcriptional responses to vaccination. The timepoints in our study (baseline, day 1, day 10) were selected to capture both innate and adaptive immune responses, while also ensuring participant safety, and retention, as well as adherence to our IRB's regulations in terms of the maximum volume of blood one can collect from older adults within a week (550 ml over 8-week period or 69 ml per week). In a previous study conducted in healthy younger adults, we showed that transcriptional innate responses to PPSV23 peaked at day 1, whereas adaptive responses peaked at day 7 and remained high until day 14[1]. We selected day 10 for this study since it enabled us to collect enough blood at multiple timepoints for the various assays used in

our study, while also capturing adaptive responses. It is possible that in this cohort adaptive responses were stronger at day 7 compared to day 10, and if additional phlebotomy had been allowed by our IRB, measurements at that time point might have provided further transcriptional signatures of vaccine responses. We want to emphasize that little is known about plasmablast responses of older adults in the context of these vaccines. It would be interesting to study this in the future and compare responses at multiple earlier timepoints (days 7, 8, 9). Interestingly, in the younger adult cohort[1], we had previously captured a significant innate response at day 1, which we did not observe in this older adult cohort. This could potentially be due to either already active innate immunity of older adults at baseline and/or the lack of additional reactivity of their myeloid cells to the pneumococcal vaccine components. In the revised manuscript, we have clarified the reasons and the necessity behind selecting these timepoints and emphasize the need to further study innate and adaptive responses of older adults to these vaccines at other time points. We also stated that we cannot exclude the possibility that timepoints earlier than day 10 might reveal additional differences.

3. With weaker PCV13 responses, increased Th17 and decreased Th1 cell frequency have been revealed. A different behavior might be expected in a different target population (e.g., infants post multiple injections)? Does it confirm previous studied (see Reference 52 and 53)?

Response: We agree with the reviewer that different signatures might be associated with responsiveness to pneumococcal vaccines across different cohorts. Unfortunately, there are no previous genomic studies investigating the baseline predictors of these vaccines in any other populations, so it is not possible to assess the specificity of this signature. However, infants and children respond strongly to these vaccines, unlike older adults, therefore it is very unlikely that they will have this baseline signature. It is possible that immuno-compromised donors might have similar signatures, future studies are needed to clarify this. We further clarified in the manuscript why we focused on this population and discussed in the revised manuscript the need to conduct similar studies in other cohorts (**pages 3, 4 and 21 in the revised manuscript**).

4. The authors concluded these findings might also give rise to novel intervention strategies to change the baseline immune phenotypes to alter the outcome of vaccine responses, e.g., by temporarily blocking the immunosuppressive or cytolytic functions of CD16+ NK cells prior to vaccination. Can the authors expand this option more in details?

Response: Our findings suggest that it could potentially be beneficial to pre-screen older individuals for their CD16$^+$ NK cell frequency and T_h1/T_h17 ratio to identify persons who may respond (or not) to PCV13 vaccination. Regarding novel interventions, while NK cell immunotherapy has had a significant impact on the treatment of human diseases[2,3], short-term NK cell inhibition may be more complicated to achieve[4]. Activating receptor blockade or inhibitory receptor ligation, the use of cytokine-mediated NK cell modulation, or epigenetic remodeling of NK cell effector functions are potential strategies to modulate NK

cell cytotoxicity. However, these intervention strategies are in their infancy and their safety and NK cell-specificity is not clear. Future work is needed to determine if local or short-term NK cell modulation is a feasible vaccine adjuvant approach. Although very important for developing new strategies in the long-term, given the infancy of this field at present, we kept the discussion about modulating NK cell activity short.

In the meantime, our results strongly suggest that simple clinical assays could be established to stratify older adults based on baseline CYTOX signature in terms of individual risk. We propose that ultimately our approach might provide clinical guidance whereby older adults with low baseline CYTOX would be encouraged to receive PCV13, whereas for individuals with high CYTOX may be more appropriate PPSV23 (**also see response to Reviewer #3, page 14**). Such strategies seeking to improve clinical outcomes by stratifying the heterogeneity of risk and underlying mechanisms lie at the core of the well-established field of *Precision Medicine*, or *Precision Vaccinology* as we articulate in our manuscript. We expanded on these points in the revised manuscript (**page 21**).

5. Can the Author comment on baseline characteristics of patient population and whether concomitant medication might influence the different observed immune responses? (I don't have access to supplementary table 1).

Response: Some of these data were provided in **Supplementary Tables S1a and S1b**. Study participants were all older than 60 years of age (average age: 68 years) and were all pneumococcal-vaccine naive. Furthermore, all the individuals were non-frail based on Frailty Index[5], and they did not have any potentially confounding immune diseases. In terms of treatments, none were taking confounding treatments (e.g., oral steroids). However, as expected for this age group, most were on other widely used medications for common well-controlled chronic diseases such as hypertension, and hyperlipidemia (provided in revised **Supplementary Table S1b**). We were also curious about the effects of these treatments on vaccine responses. However, due to the heterogeneity in terms of the treatments and the sparsity of donors receiving any specific treatment (20% for the most frequent treatments), we did not have the statistical power to draw meaningful conclusions about the effects of specific treatments on

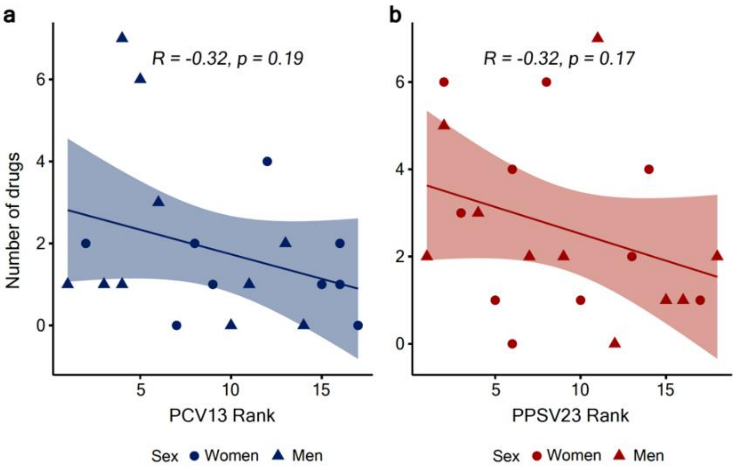

Figure L1. Correlation analyses between vaccine responsiveness rank and the number of medications taken by the donors for the PCV13 cohort (**a**) and the PPSV23 cohort (**b**). Note that there is no significant association for either vaccine.

vaccine responses. Nonetheless, as commonly done in aging research, we studied whether the number of concomitant medications taken by participants had any association with vaccine responsiveness (PCV13 Rank and PPSV23 Rank) and showed that there is no significant association (**Fig. L1**). The detailed medication history data and these new analyses are included in the revised manuscript (provided in **Fig. s1d and Supplementary Table S1b**).

6.Have the Authors investigated whether the reported differences in baseline T cell activation are impacting memory B cell responses to PCV vaccination at latest time point?

Response: We agree with the reviewer that this is an important question. However, due to limitations in blood volume we could collect from this population (**see above page 2**), we could not perform additional assays to study antigen-specific B cells and their responses in this study. It would be a very interesting addition to a future study. We have discussed this as a limitation of the current study in the revised manuscript.

Minor

1.Line 70. “In the US, the current recommendation for adults aged ≥ 65 years is to give a conjugated vaccine (PCV15/PCV20) followed by PPSV23 at least one year later”. If PCV20 is used, a dose of PPSV23 is not indicated.

Response: These recommendations have recently changed. We thank the reviewer for catching this, based on the latest CDC recommendations “All patients above 65 years should be administered either one dose of PCV20 or a combination of PCV15 followed by PPSV23 at least one year later[6] ”. We have rewritten this section accordingly in the revised manuscript (**page 4**).

****

Reviewer #2

Remarks to the Author:

The authors combine serology, phagocytosis assays (OPA), bulk and single cell RNAseq and cutoff to present a detailed evaluation of factors that influence antibody responses to the conjugated (PCV) and unconjugated (PPV) pneumococcal vaccines. Their data indicates that poor response to PCV correlated with higher age (expected), perhaps male sex, and (the novel findings) high baseline Th17 and CD16+ NK cell numbers/proportions and a corresponding RNA signature for cytotoxicity genes. The data are all presented and very extensive, and I found the revised manuscript of significant interest.

Response: We thank the reviewer for finding our work of significant interest!

Major issues.
There are two really significant issues with the data interpretation:

1. Although they present males as having worse responses than females this different was not statistically significant (Figs 1F) although very very close. This makes all the male/female comparisons further on tricky to fully support. For the male male data has this been adjusted for age? Or at least what is the median age and range for males v females?

Response: We appreciate the reviewer's careful examination of our data. We put extra effort into recruiting the same number of men and women who are clinically comparable for both vaccine cohorts. Therefore, men and women cohorts are comparable in terms age distribution, BMI, and Frailty index values (see below **Table L1** and **Supplementary Tables S1a and S1b** in the revised manuscript) for both arms of the study, which gave us confidence for the observed sex difference trends. Although the sex differences in PCV13 responses did not reach statistical significance (p=0.079 for Rank in **Fig. 1f** in the revised manuscript), we were encouraged by the fact that, for PCV13, 4 out of 5 strong responders were female (with a median vaccine responsiveness rank of 12 (95% confidence interval [7.38, 15.29]) and 4 out of 5 weak responders were male (with a median vaccine responsiveness rank of 5.5 (95% confidence interval [3.86, 10.34]). Such a skew in responsiveness was not observed for PPSV23. Hence, we decided to include these results in our manuscript. In fact, Reviewer #3 agreed with us and found this observation very interesting.

Our enthusiasm for the observed sex differences further strengthened based on new analyses we have conducted to study the effects of high baseline titers on vaccine responses. Using a previously published strategy[7], we uncovered donors who have high baseline OPA titers, who are already protected hence non-informative in terms of understanding vaccine responsiveness. In the PCV13 arm, three donors were identified as non-informative since they had higher baseline titers for the majority of serotypes. Interestingly, when the non-informative donors were excluded from our comparisons, the observed sex differences reached statistical significance for PCV13 (p=0.026 for Rank). We still did not detect any sex differences in the PPSV23 arm even upon the exclusion of two non-informative donors with high baseline titers for this group. These new analyses and observations are further explained in the letter (**see page 11 in the letter for further details**) and included in the revised manuscript (**Fig. 6 and Supplementary Table S9**).

Table L1. Cohort level demographics summary for age (in years), BMI, and frailty index. P-values were calculated using the two-sided *t-test* for continuous variables, considering each donor parameter individually. *ns = not significant (p >0.05).

	**All Subjects (n=39)**	**PCV13 (n=19)**	**PPSV23 (n=20)**	**p-value** PCV13 vs PPSV23
**Age** (in years) Mean ± SD (range)				
Both	68 ± 8 (60-88)	67 ± 6 (60-81)	69 ± 9 (60-88)	ns
Women	68± 7 (60-88)	67 ± 5 (60-75)	67 ± 9 (60-88)	ns
Men	69± 9 (60-84)	68 ± 7 (60-81)	71 ± 10 (60-84)	ns
Age (Women vs. Men)	ns	ns	ns	
**BMI** (in kg/m2) Mean ± SD (range)				
Both	26.02 ± 5.07 (19.22-42.95)	24.83 ± 3.77 (19.22-32.86)	27.16 ± 5.94 (21.39-42.95)	ns
Women	24.43 ± 4.22 (19.22-38.25)	23.14 ± 2.48 (19.22-27.79)	25.58 ± 5.19 (21.39-38.25)	ns
Men	27.54 ± 5.44 (19.52-42.95)	26.36 ± 4.18 (19.52-32.86)	28.73 ± 6.48 (22.23-42.95)	ns
BMI (Women vs. Men)	ns	ns	ns	
**Rockwood Frailty Index** (count/65) Mean ± SD (range)				
Both	0.054 ± 0.037 (0.00-0.153)	0.054 ± 0.036 (0.00-0.138)	0.053 ± 0.034 (0.015-0.153)	ns
Women	0.051 ± 0.032 (0.015-0.123)	0.056 ± 0.037 (0.015-0.123)	0.046 ± 0.027 (0.015-0.092)	ns
Men	0.057 ± 0.042 (0.00-0.153)	0.053 ± 0.045 (0.00-0.138)	0.061 ± 0.041 (0.015-0.153)	ns
Rockwood Frailty Index Women vs. Men	ns	ns	ns	

2. There are multiple places in the figures where correlations are shown for PCV to immune data that are shown not to be present for PPV.

Response: We have carefully revised the revised manuscript and included the missing correlation plots for PPSV23 (see new supplementary figures **Fig. s6d, e**).

However, the data shown in figure 2A suggests there is a significant plasma blast signature for many of the PPV non-responders; the authors comment on this and suggest this represents antibody responses to serotypes that they have not analysed in their serology data, and this is probably true. However, if so that completely invalidates the ranking for the PPV recipients (not for the PCV recipients) and means the differences in the immune factors correlated to good/bad response in PCV versus PPV recipients are also not really valid. The PPV data can be used as a sort of negative control, but I don't think can be interpreted was showing PPV poor responders do not have the same immune profile as PCV poor responders. This affects multiple aspects of the text and the figures. They could do the serology for the 10 or so missing serotypes for the PPV recipients; but the number of responders is high so may not be enough differential for all the correlations to have adequate power.

Response: We agree with the reviewer. Ideally, we would have quantified responses to all 23 serotypes in PPSV23. Unfortunately, this was not possible since for a number of reasons (summarized below) we opted to use OPA assays to quantify vaccine responses. Immune responses to pneumococcal vaccines can be determined by either measuring antibody levels with ELISA or antibody function with OPA assays. Many older adults have high antibody levels at baseline prior to vaccination, therefore functional OPA assays represent a more accurate and more dynamic measure of vaccine responsiveness among older adults[8,9]. Furthermore, FDA considers OPA assays as the state-of-the-art indicator of bacterial pneumonia vaccine responses and used only OPA results to license these vaccines[10].

In view of all of the above considerations, we decided to use OPA assays in our study. However, this decision came at a cost in terms of the biological material required to perform this assay. The OPA test requires large amount of serum for every single tested serotype[11]. Due to the frequency of the early visits, participant ages, IRB safety concerns (**see above page 2**) and samples needed for PBMC profiling, we had limited material for quantifying vaccine responses. The volume of recovered serum after blood was processed for other assays varied between 1.3 and 2 mL, which, in the case of some donors, was enough only for the OPA test for the 13 serotypes studied here. Therefore, unfortunately, due to this logistical challenge, we are unable to measure the other 10 serotypes using OPA.

However, we want to emphasize that the 13 serotypes tested here were selected on the basis of being the most prevalent ones in the community at the time of our study (seasons 2017-2018)[12]. In fact, that is the reason why these specific serotypes are selected for PCV13 vaccine formulation. Therefore, our quantification closely captures whether these donors are likely to be protected in the "real" world, although it might not capture the responsiveness to other less prevalent untested serotypes. Also, by quantifying the shared serotypes, we were able to conduct a side-by-side comparative analysis at the serotype level for these two vaccines. In the revised manuscript, we have included these details on the choice of assay and choice of tested serotypes and have rewritten the results for PPSV23 responses to clarify that our ranking strategy is restricted to the prevalent serotypes (hence clinical protection).

To further assess the value of our ranking strategy, we found a small cohort (n=6) where vaccine responses to PPSV23 is quantified using OPA titers for all 23 serotypes[13]. We used these data to compare ranking of donors based on 13 versus 23 serotypes (details in revised Methods, page 31), which revealed a strong correlation between these two ranking strategies (Pearson correlation R= 0.91, p = 0.013, **Fig. L2**). These new analyses suggest that calculating the responsiveness based on the 13 serotypes serves as a good proxy to understand overall responsiveness to the PPSV23 vaccine. We have incorporated this analysis into the revised manuscript (**Fig. s1g in the revised manuscript**) and acknowledged the fact that we could not quantify responsiveness to all serotypes, due to the lack of enough biological material, as a limitation of our study.

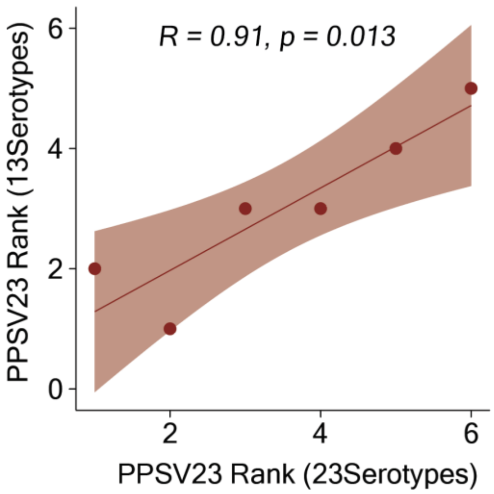

Figure L2. Correlation analyses of PPSV23 Rank derived from all 23 serotypes and the rank derived from the subset of 13 serotypes used in our study.

A few less major issues:
1. Can the authors make it clearer how much of the CYTOX signatures associated with poor response from bulk RNAseq is shown by the scRNAseq to originate from the CD16+ NK cells rather than Th17 etc

Response: To address this question, we first calculated a CYTOX score at the single cell resolution using the average expression of genes that are in the CYTOX module (n=86). The CYTOX score was highest in NK cell populations followed by the cytotoxic $CD8^+$ T cell populations, as would be expected (**Fig. L3a**). When we compared the CYTOX score of strong responders (SR) and weak responders (WR) for the memory $CD4^+$ T cell cluster, we did not detect a significant difference (p=0.79). Next, we zoomed into the memory $CD4^+$ T cell cluster and further clustered these cells and uncovered subclusters that express markers for T_h1, T_h2, T_h17, T_h22, and central memory cells (**Fig. L3b-c**). There were no differences between CYTOX scores of cells from SR and WR in these $CD4^+$ memory subsets (**Fig. L3e**). Furthermore, we did not detect significant differences between SR and WR in terms of the CYTOX scores for other T cell

memory subsets including TEMRAs (**Fig. L3e**). These new analyses, which are included in the revised manuscript (**Fig. s9** in the revised manuscript), further reinforced that the CYTOX signature most likely stems from the CD16$^+$ NK population.

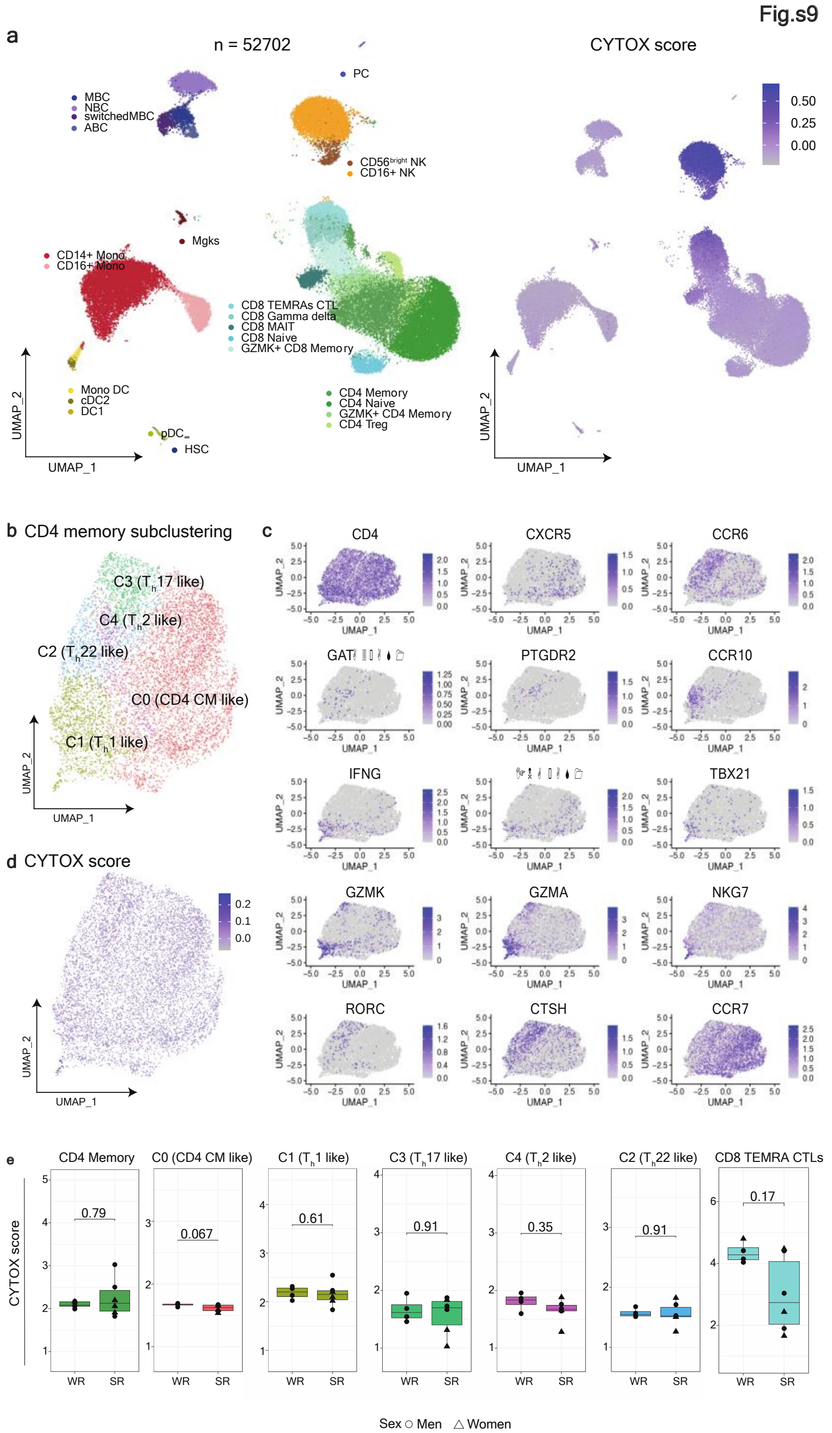

Figure L3. The CYTOX Signature stems from CD16^{+} NK Cells. a) UMAP representation of PBMCs derived from 11 PCV13 donors, consisting of 6 strong responders (SR) and 5 weak responders (WR). This

visualization encompasses 24 clusters from a total of 52,702 cells, each color-coded by their respective immune cell type. An accompanying feature plot elucidates the CYTOX scores at single cell resolution using the expression of 86 genes associated with the PCV13 vaccine responsiveness. These 86 genes are a subset from the CYTOX module which showed a correlation of > 0.5. b) Subclustering of $CD4^+$ memory cells highlight subsets expressing marker genes for C0 (central memory like cells), C1 (T_h1 like), C2 (T_h22 like), C3(T_h17 like) and C4 (T_h2 like). c) Feature plot to show marker gene expression for memory $CD4^+$ subsets. d) A feature plot showing CYTOX score for memory $CD4^+$ T cells. e) Comparison of CYTOX scores for SR and WR for each memory $CD4^+$ subset and $CD8^+$ TEMRA CTLs. A Wilcoxon Rank sum test was employed to assess differences in the CYTOX scores between the PCV13 SR and WR across these CD4 memory subsets and $CD8^+$ TEMRA CTLs.

2. Some data has negatively correlated NK cell numbers with Th17 CD4s; can the authors discuss more whether these are two independent markers of poor response or could be functional related and what mechanisms might be involved?

Response: We agree that this is an important question. However, current understanding of how NK cells regulate T_h cell subsets, including T_h1 and T_h17 cells, is quite limited, especially in humans. Less than a handful of papers have examined the role of NK cells in expanding or controlling T_h17 cells in the settings of autoimmune inflammation, transplantation, pregnancy, or infection[14–18] . Generally, NK cells can modulate T_h17 and T_h1 cell expansion *via* IL-17A or IFNg secretion, or by direct killing of activated T_h cells. Additionally, T_h cell killing by NK cells is sensitive to T_h cell-secreted IL-2 amounts, and T_h cell expression of HLA-E (inhibitory), or NKG2D or DNAM-1 ligands (stimulatory). Thus, it is likely that NK cell regulation of T_h cell expansion is tissue- and disease-specific and mechanistically complex. Due to this knowledge gap, we limited our discussion to published studies in humans and animal models that have demonstrated a negative correlation of NK cell activity and antibody titers in vaccination or disease settings[14,19–22]. Future studies are needed to evaluate the magnitude and mechanisms of NK cell regulation of the T_h cell response in animal models and in humans. This is discussed in the revised manuscript (**page 20**).

3. the Th1/17 ratio and age correlation need to be in figure 3 (and maybe some of the other data eg NCAM1 expression)

Response: Thank you for this suggestion. We have moved both figures to the corresponding main panels in the revised manuscript (**Fig. 3e and 3h**).

4. One for the statistical editor; are they happy adequate correction for multiple comparisons has been specifically for correlations between datasets?

Response: Benjamini Hochberg test is used for multiple test correction when needed. We have clarified this in revised manuscript.

Minor issues:
lines 67 – 68 decline with age – this is not unexpected now given the data we have for all other vaccines

Response: As suggested, we have updated this in the revised manuscript.

lines 69-70 not for improving immunogenicity but for extending range of serotypes covered
lines 110 to 114 could be merged ('no differences between vaccine group or males versus females for….')

Response: As suggested, we have merged these lines in the revised manuscript.

fig 1b and c why is age a range?
(give a age and not age range)

Response: We have provided the age ranges based on the *Nature Immunology* journal guidelines. After consultations with the journal as well as our IRB office, we now provided the actual age of donors in the revised manuscript.

so is rank purely decided on by the OPA data? that is fine but for clarity this needs to be a bit more explicit perhaps

Response: Yes, rank is purely derived from the OPA data using a multivariate approach. This is clarified in the revised manuscript.

interesting how many subjects have really poor responses even to PCV - is this due to high baseline antibody to those serotypes which is perhaps unlikely but needs checking. Talk abt if any ?

Response: We conducted comprehensive new analyses to address this interesting question, which is now included in the manuscript **(pages 15-17).** Previous studies in pneumococcal vaccines, including ours, showed that donors with high baseline antibody levels tend to have lower fold increases upon vaccination[23], which was also observed in our cohort (**Fig s2a, b**). Such donors, who are likely already protected, can affect conclusion related to vaccine responsiveness and vaccine efficacy. However, identification of non-informative (high baseline) donors are particularly challenging for bacterial pneumonia vaccines, due to the variability in serotype baselines (some serotypes have consistently higher baselines for example serotypes 9V, 19A, and 19F in our cohort **Fig. 6a**) and due to the variability in baseline titer levels among different cohorts. To overcome this, our team has previously developed a novel analytical strategy[7], to bin donors into three groups based on their immune responses to bacterial pneumonia vaccines: strong responders (low

baseline, high fold increase in OPA titers), weak responders (low baseline, low fold increase in OPA titers), and non-informative donors (high baseline titers)[7]. Using this strategy (**Methods described in Page 31**), we identified 10 strong, 6 weak, and 3 non-informative donors in the PCV13 arm and 11 strong, 7 weak, and 2 non-informative donors in the PPSV23 arm (**Supplementary Table S9a and S9b** in the revised manuscript). We repeated all the analyses after excluding donors with the high-baseline titers (non-informative) and observed that all the associations with vaccine responsiveness (demographic, cellular, transcriptomic) are still significant and in some instances even stronger (e.g., sex differences). These new analyses are included in the revised manuscript (**Fig. 6, Supplementary Tables S9a and S9b**). In addition, in view of these new analyses, we decided to use the terminology ‘strong’ and ‘weak’ responders throughout the paper, instead of ‘responders’ and ‘non-responders’.

line 321 this text is too early as pre-gene signature associations: 'Together these data establish that the increased frequency of CD16+ NK cells bear the CYTOX signature that is associated with reduced PCV13 responses'

Response: These changes are incorporated in the revised manuscript.

fig 2A and suppl fig 3A some sort of translation of genes for what they encode would be useful

Response: We have provided details on proteins encoded by the genes in **Fig. 2a** and **Fig. s3a** in the revised manuscript (provided in **Supplementary Table S3k**).

supple fig 6b symbols covered over by p value text

Response: This is corrected in the revised manuscript.

line 376 - should be S. pneumoniae
Response: This is corrected in the revised manuscript.

****
Reviewer #3:
Remarks to the Author:
General Comments
At first, I read this paper finding it incremental and confirmatory either of past results or assumptions. But as I read in more detail, I found many gems hidden in the data and in the text. The study is excellent and important, but the write-up needs to be expanded and clarified to draw proper attention to the importance of the work.

Response: We thank the reviewer for his/her enthusiasm for our study and for the constructive feedback!

Specific Comments

1.Title: the very important implication of the data that is missing from the title is that men and women respond differently to vaccine. This should be included in the title and abstract.

Response: We have highlighted the sex differences in PCV13 responses in the abstract of the revised manuscript.

2. Abstract; Needs to draw attention to better PCV13 response in women than men, and underlying mechanisms.

Response: We agree with the reviewer that this is a very exciting finding, however, we were hesitant to add this to the title and abstract since we were close to the statistical significance cutoff ($p = 0.079$) but did not reach it (also pointed out by Reviewer #2). In response to Reviewer #2, we conducted new analyses to uncover and eliminate donors with high baseline titers since they are already immune and non-informative for assessing the efficacy of vaccines (see new **Fig. 6** in the revised manuscript). With the exclusion of these non-informative donors, sex differences reached the statistical significance threshold ($p=0.026$), hence we included the observed sex differences in PCV13 responses in the abstract **(page 2).** Since our study compares responses to both conjugated and unconjugated vaccines, our title highlights the fact that their baseline predictors are distinct, which we believe is appropriate.

Regarding underlying mechanisms, in our previous cross-sectional studies, we showed that T cells of men appear to demonstrate an accelerated aging phenotype when compared to women who had been matched on a variety of clinically relevant criteria [24]**.** We suspect that these differences in the rate of T cell aging might be contributing to the observed sex differences in PCV13 responses. We have elaborated this in the revised manuscript **(page 19)**. Furthermore, in this study, we showed that women may have a benefit over men since on average they have higher frequency of T_h1 and lower frequency of T_h17 cells compared to clinically matched men, which correlated with their improved PCV13 responses. Further research is needed to uncover the mechanistic reasons behind the sex differences in these T cell populations.

3. Intro: I found the repeated reference to T-independent responses to polysaccharide somewhat irritating. Certainly, this is an immunological definition, derived from animal models, but it is not a complete description of the response of healthy adult humans to polysaccharide vaccine. It is even mis-leading as the data in the Results and the subsequent Discussion acknowledge. A proper introduction as to how PCV and PPV are handled in adults, to include discussion of marginal zone and follicular function in spleen, is an essential part of this paper that is missing.

Response: We agree with the reviewer and have dropped references to T-independent responses; indeed, our data suggest that PPSV23 is also affecting ICOS^{+} Tfh cells. In addition, we also included a detailed paragraph (copied below) in the revised manuscript (**pages 2,3**) to further elaborate on this.

"PCV13, a polysaccharide-conjugated pneumococcal vaccine, induces T cell-dependent activation and expansion of B2 follicular cells, promoting the generation of memory B cells and the differentiation of long-lived plasma cells[25]. In contrast, PPSV23 consists of unconjugated pneumococcal polysaccharides, which are usually classified as type 2 T cell-independent antigens based on studies performed in mice and are able to induce primary antibody responses in the absence of CD4^{+} T cell collaboration[26]. It has been shown that responses to unconjugated polysaccharides are predominantly mediated by the activation of a specific subset of IgM^{+} B cells, named marginal zone B cells (MZ). These cells harbor a memory-like phenotype (IgM^{+}IgD^{+}CD27^{+}) displaying hypermutated immunoglobulin genes and showing a high expression of the complement receptor 2 (CD21)[27,28]. MZ B cells are not fully mature in newborns and children under 2 years old, a reason that might explain the failure of unconjugated polysaccharide vaccines to elicit adequate immune responses in this population[27,29–31]. Interestingly, older adults show a marked decrease in the percentages of total memory B cells and MZ B cells, suggesting an association between their decline and poorer serological responses against polysaccharide antigens[32]. Upon activation, MZ B cells terminally differentiate into IgM, IgG, and IgA-secreting plasma cells[33,34]. Although evidence indicates MZ B cell activation and IgM production are T cell-independent, it is possible that IgG and IgA class switching might rely on the presence of noncognate T cells that secrete IL-21 and/or trigger the CD40/CD40L signaling axis[34]."

4. Results: The results are very impressive in their rigour and careful analysis. Really detailed descriptions of both B and T cell function are presented. The immunoglobulin, OPA and plasmablast data might be somewhat incremental on what has been published before, but the RNA work and the T cell descriptions are novel, interesting and of translational importance.

Response: We thank the reviewer for appreciating our work and its translational significance!

5. Discussion: I would like to see a more comprehensive discussion. It is well known and not cited that PPV causes an excess of pneumonia (but not death) in vaccinated elderly adults, probably by immunoglobulin and maybe by B cell depletion. This should be reviewed and discussed.

Response: We agree with the reviewer and have expanded the discussion in the revised manuscript (**pages 20,21**). We are aware of the observation that capsular polysaccharidic antigens in pneumococcal vaccines can neutralize pre-existing antibodies or paralyze B cells, potentially rendering a vaccinee more susceptible to infections[35–37]. This could theoretically contribute to an increased incidence of pneumonia (though not death) in vaccinated older adults. Nonetheless, PCV has shown to effectively prevent pneumococcal pneumonia and invasive pneumococcal disease in older adults at the population level and in large clinical

trials[38,39]. A meta-analysis of 18 randomized trials and over 64,500 individuals found that pneumococcal polysaccharide vaccines reduce the risk of both invasive pneumococcal disease and noninvasive pneumococcal pneumonia[40]. Furthermore, the widespread use of PCV in children prior to its use in older adults has resulted in a decline in the prevalence of certain pneumococcal strains causing pneumonia in the older adult population.

The APC and T-cell role in both PCV and PPV response should be compared. This will allow a proper appreciation of the paper.

Response: In flow cytometry data, we quantified cDC1, DC2, pDC cell subsets longitudinally along with B and T cell subsets. However, we didn't detect significant changes upon vaccination or significant association with PCV13 and PPSV23 responsiveness at the baseline in these subsets (**Fig. 3b** in the revised manuscript) for either vaccine. The only cell type for which we detected a significant change upon vaccination was ICOS$^+$ T_{fh} cells, the frequency of which increased at day 10 for both vaccines (**Fig. 3a** bottom panel in the revised manuscript). In single cell RNA-seq data, we detected DC subsets (monoDC, cDC1, DC2, and pDC), as well as B, CD4$^+$ and CD8$^+$ T cell subsets. Among these subsets, only naïve CD8$^+$ T cells were significantly different between strong and weak responders (p=0.03), where strong responders had more naïve CD8$^+$ T cells, likely due to the effects of aging on vaccine responses. We have included and discussed these results in the revised manuscript (**pages 11,13**).

The suggestion of personalised vaccination should be more fully expanded. Would simple differentiation by gender be enough, or are there additional (simple, available) tests that could pick out the Th1/Th17 dependent functions described?

Response: This is one of the major findings of our study and we have expanded this section in the revised manuscript **(page 21).** In summary, we believe that it will be feasible to establish simple blood-based clinical assays to assess whether an individual has the CYTOX signature. For example, it is feasible to quantify the baseline expression levels of *NCAM1* using qRT-PCR or to quantify the baseline % of CD16$^+$ NK, T_h1 and T_h17 cells using flow cytometry. Although our study suggested that men are more likely to have the CYTOX signature, there are exceptions to this rule, so it will be more precise to assess the CYTOX signature with these simple assays prior to vaccination rather than stratifying the population based on only biological sex. We also observed that the CYTOX signature only affects responsiveness to PCV13, so donors with high CYTOX signature (e.g., high *NCAM1* expression) are more likely to benefit from the PPSV23 vaccine.

In sum, an effective simple strategy is to stratify the population based on the CYTOX signature. Ultimately, these tests could provide this information in a "Point of Care" manner, thus permitting clinicians to act on such information within the context of a clinical encounter. Donors with low CYTOX would then be encouraged to receive PCV13, whereas donors with high CYTOX would be more likely to benefit from

PPSV23. Furthermore, as noted in our responses to Reviewer #1, such strategies seeking to improve clinical outcomes by stratifying the heterogeneity of risk and underlying mechanisms lie at the core of Precision Medicine or Precision Vaccinology.

Journal requested comments
Summary of the key results
Older men and women received either PPV or PCV and subsequent immune responses were described in detail. IgG to polysaccharides, OPA data, plasmablast frequency and function as well as T-cell phenotype and function are described in a uniquely comprehensive study.

Originality and significance: if not novel, please include reference
The originality is not in the question that is addressed in this study, but in the extremely detailed manner in which it is answered. The significance of the paper is a profound translational suggestion that personalised vaccination may be considerably more effective than the current strategy.

Data & methodology: validity of approach, quality of data, quality of presentation
This study succeeds in being as completely thorough in addressing the question as modern methods will allow. The data are very, very extensive and the methodology cutting-edge. The data are presented in a very clear and comprehensive manner.

Appropriate use of statistics and treatment of uncertainties
These seem robust to me, but I would defer to more expert opinion.

Conclusions: robustness, validity, reliability
This is a state-of-the-art paper.
Suggested improvements: experiments, data for possible revision
I have put some suggestions about emphasis, implications and translation in the text; no additions to the experiments or analysis.

References: appropriate credit to previous work?
I've mentioned the concerns about PPV (not mentioned), the presentation of known immunology (impaired responses to PPV, T cell role in "T-independent" responses).

Clarity and context: lucidity of abstract/summary, appropriateness of abstract, introduction and conclusions (these comments already above).

REREFERENCES:

1. Obermoser, G. *et al.* Systems scale interactive exploration reveals quantitative and qualitative differences in response to influenza and pneumococcal vaccines. *Immunity* **38**, 831–844 (2013).
2. Laskowski, T. J., Biederstädt, A. & Rezvani, K. Natural killer cells in antitumour adoptive cell immunotherapy. *Nat. Rev. Cancer* **22**, 557–575 (2022).
3. Smith, D. M., Schafer, J. R., Tullius, B., Witkam, L. & Paust, S. Natural killer cells for antiviral therapy. *Sci. Transl. Med.* **15**, eabl5278 (2023).
4. Cox, A. *et al.* Targeting natural killer cells to enhance vaccine responses. *Trends Pharmacol. Sci.* **42**, 789–801 (2021).
5. Rockwood, K. *et al.* A global clinical measure of fitness and frailty in elderly people. *CMAJ Can. Med. Assoc. J.* **173**, 489–495 (2005).
6. Shah, A. A., Fields, H. & Wallace, M. R. Navigating the Changes in Pneumococcal Immunizations for Adults. *Am. Fam. Physician* **92**, 456–458 (2015).
7. LaFon, D., Kim, Y., Burton, R., Dransfield, M. & Nahm, M. Pneumococcal antibody function for immunologic evaluation: normal results in older adults, and a novel analytical model for vaccine response. *J. Clin. Immunol.* **41**, 1964–1968 (2021).
8. Song, J. Y., Nahm, M. H. & Moseley, M. A. Clinical implications of pneumococcal serotypes: invasive disease potential, clinical presentations, and antibiotic resistance. *J. Korean Med. Sci.* **28**, 4–15 (2013).
9. Durando, P. *et al.* Experience with pneumococcal polysaccharide conjugate vaccine (conjugated to CRM197 carrier protein) in children and adults. *Clin. Microbiol. Infect. Off. Publ. Eur. Soc. Clin. Microbiol. Infect. Dis.* **19 Suppl 1**, 1–9 (2013).
10. Centers for Disease Control and Prevention (CDC). Licensure of 13-valent pneumococcal conjugate vaccine for adults aged 50 years and older. *MMWR Morb. Mortal. Wkly. Rep.* **61**, 394–395 (2012).
11. Burton, R. L. & Nahm, M. H. Development and validation of a fourfold multiplexed opsonization assay (MOPA4) for pneumococcal antibodies. *Clin. Vaccine Immunol. CVI* **13**, 1004–1009 (2006).
12. Knoll, M. D. *et al.* GLOBAL SEROTYPE DISTRIBUTION OF INVASIVE PNEUMOCOCCAL DISEASE IN OLDER CHILDREN AND ADULTS: AGEDD STUDY.

13. Sterrett, S. *et al.* Peripheral CD4 T follicular cells induced by a conjugated pneumococcal vaccine correlate with enhanced opsonophagocytic antibody responses in younger individuals. *Vaccine* **38**, 1778–1786 (2020).
14. Ali, A. *et al.* Natural killer cell immunosuppressive function requires CXCR3-dependent redistribution within lymphoid tissues. *J. Clin. Invest.* **131**, e146686 (2021).
15. Arbelaez, C. A., Palle, P., Charaix, J. & Bettelli, E. STAT1 signaling protects self-reactive T cells from control by innate cells during neuroinflammation. *JCI Insight* **7**, e148222 (2022).
16. Fu, B. *et al.* Natural killer cells promote immune tolerance by regulating inflammatory TH17 cells at the human maternal-fetal interface. *Proc. Natl. Acad. Sci. U. S. A.* **110**, E231-240 (2013).
17. Li, J. *et al.* Natural killer cells regulate Th1/Treg and Th17/Treg balance in chlamydial lung infection. *J. Cell. Mol. Med.* **20**, 1339–1351 (2016).
18. Pallmer, K. & Oxenius, A. Recognition and Regulation of T Cells by NK Cells. *Front. Immunol.* **7**, (2016).
19. Rydyznski, C. E. *et al.* Affinity Maturation Is Impaired by Natural Killer Cell Suppression of Germinal Centers. *Cell Rep.* **24**, 3367-3373.e4 (2018).
20. Rydyznski, C. *et al.* Generation of cellular immune memory and B-cell immunity is impaired by natural killer cells. *Nat. Commun.* **6**, 6375 (2015).
21. Reighard, S. D. *et al.* Therapeutic Targeting of Follicular T Cells with Chimeric Antigen Receptor-Expressing Natural Killer Cells. *Cell Rep. Med.* **1**, 100003 (2020).
22. Waggoner, S. N., Cornberg, M., Selin, L. K. & Welsh, R. M. Natural killer cells act as rheostats modulating antiviral T cells. *Nature* **481**, 394–398 (2012).
23. Hare, N. D., Smith, B. J. & Ballas, Z. K. Antibody response to pneumococcal vaccination as a function of preimmunization titer. *J. Allergy Clin. Immunol.* **123**, 195–200 (2009).
24. Márquez, E. J. *et al.* Sexual-dimorphism in human immune system aging. *Nat. Commun.* **11**, 751 (2020).
25. Rappuoli, R., De Gregorio, E. & Costantino, P. On the mechanisms of conjugate vaccines. *Proc. Natl. Acad. Sci. U. S. A.* **116**, 14–16 (2019).
26. Mond, J. J., Lees, A. & Snapper, C. M. T cell-independent antigens type 2. *Annu. Rev. Immunol.* **13**, 655–692 (1995).

27. Weller, S. *et al.* Human blood IgM 'memory' B cells are circulating splenic marginal zone B cells harboring a prediversified immunoglobulin repertoire. *Blood* **104**, 3647–3654 (2004).
28. Weller, S. *et al.* T-independent responses to polysaccharides in humans mobilize marginal zone B cells prediversified against gut bacterial antigens. *Sci. Immunol.* **8**, eade1413 (2023).
29. Cowan, M. J. *et al.* Pneumococcal polysaccharide immunization in infants and children. *Pediatrics* **62**, 721–727 (1978).
30. Douglas, R. M., Paton, J. C., Duncan, S. J. & Hansman, D. J. Antibody Response to Pneumococcal Vaccination in Children Younger than Five Years of Age. *J. Infect. Dis.* **148**, 131–137 (1983).
31. Timens, W., Boes, A., Rozeboom-Uiterwijk, T. & Poppema, S. Immaturity of the human splenic marginal zone in infancy. Possible contribution to the deficient infant immune response. *J. Immunol. Baltim. Md 1950* **143**, 3200–3206 (1989).
32. Shi, Y. *et al.* Regulation of aged humoral immune defense against pneumococcal bacteria by IgM memory B cell. *J. Immunol. Baltim. Md 1950* **175**, 3262–3267 (2005).
33. Clutterbuck, E. A. *et al.* Pneumococcal conjugate and plain polysaccharide vaccines have divergent effects on antigen-specific B cells. *J. Infect. Dis.* **205**, 1408–1416 (2012).
34. Weill, J.-C., Weller, S. & Reynaud, C.-A. Human marginal zone B cells. *Annu. Rev. Immunol.* **27**, 267–285 (2009).
35. Pichichero, M. E. Immunological paralysis to pneumococcal polysaccharide in man. *Lancet Lond. Engl.* **2**, 468–471 (1985).
36. Sood, S. K. & Daum, R. S. Disease caused by Haemophilus influenzae type b in the immediate period after homologous immunization: immunologic investigation. *Pediatrics* **85**, 698–704 (1990).
37. Daum, R. S. *et al.* Decline in serum antibody to the capsule of Haemophilus influenzae type b in the immediate postimmunization period. *J. Pediatr.* **114**, 742–747 (1989).
38. Shapiro, E. D. *et al.* The protective efficacy of polyvalent pneumococcal polysaccharide vaccine. *N. Engl. J. Med.* **325**, 1453–1460 (1991).
39. Jackson, L. A. *et al.* Effectiveness of pneumococcal polysaccharide vaccine in older adults. *N. Engl. J. Med.* **348**, 1747–1755 (2003).

40. Moberley, S., Holden, J., Tatham, D. P. & Andrews, R. M. Vaccines for preventing pneumococcal infection in adults. *Cochrane Database Syst. Rev.* **2013**, CD000422 (2013).

Decision Letter, first revision:

15th Oct 2023

Dear Duygu,

Thank you for submitting your revised manuscript "Distinct baseline immune characteristics associated with responses to conjugated and unconjugated pneumococcal polysaccharide vaccines in older adults" (NI-A35702A). It has now been seen by two of the original referees and their comments are below. The reviewers find that the paper has improved in revision, and therefore we'll be happy in principle to publish it in Nature Immunology, pending minor revisions to comply with our editorial and formatting guidelines.

We will now perform detailed checks on your paper and will send you a checklist detailing our editorial and formatting requirements in about a week. Please do not upload the final materials and make any revisions until you receive this additional information from us.

If you had not uploaded a Word file for the current version of the manuscript, we will need one before beginning the editing process; please email that to immunology@us.nature.com at your earliest convenience.

Thank you again for your interest in Nature Immunology Please do not hesitate to contact me if you have any questions.

Kind regards,

Laurie

Laurie A. Dempsey, Ph.D.
Senior Editor
Nature Immunology
l.dempsey@us.nature.com
ORCID: 0000-0002-3304-796X

Reviewer #2 (Remarks to the Author):

The authors have made great efforts to address my (and the other reviewers') comments. I think they

have successfully addressed the points raised (specifically the male / female difference and ability to identify weak/strong responders for PPV23 group).

Reviewer #3 (Remarks to the Author):

Review of R1
This paper is a clear and comprehensive comparison of the immunological response to PPV and PCV in elderly people.

This comparison has been done before, but never in such a complete and in-depth manner. The very extensive analyses are presented in a clear and comprehensive manner, allowing translational conclusions to be drawn.

The data are compelling, the analysis clear and complete.

The conclusion is robust, with a clear description of the similarity and differences between the two vaccine responses.

One suggestion for an added discussion - are the baseline differences in the responder groups likely to be constitutive or a temporary response to recent infection? This would affect the translational implication and so if there are reasons to suspect that they are not transitory, these should be drawn out.

I also have a few minor comments/typos for revision.
Line 55 "were FDA" (past tense)
Line 84 insert "against IPD" for clarity
Line 532, 536 and 598 missing prepositions (the, the, a respectively)
line 609 polysaccharide

The references might benefit from the work of Neil French (not the reviewer!) in describing the response to vaccine in people with HIV (core papers in Lancet and NEJM) as this is an immunocompromised group with a very stark difference in response to PPV (harm) and PCV (benefit). I would not insist on this point if the authors and editors feel differently.

Clarity and context: Now a very clear TI explanation in the introduction and discussion, thank you.

Final Decision Letter:

Dear Duygu,

I am delighted to accept your manuscript entitled "Distinct baseline immune characteristics associated with responses to conjugated and unconjugated pneumococcal polysaccharide vaccines in older adults" for publication in an upcoming issue of Nature Immunology.

Over the next few weeks, your paper will be copyedited to ensure that it conforms to Nature Immunology style. Once your paper is typeset, you will receive an email with a link to choose the

appropriate publishing options for your paper and our Author Services team will be in touch regarding any additional information that may be required.

After the grant of rights is completed, you will receive a link to your electronic proof via email with a request to make any corrections within 48 hours. If, when you receive your proof, you cannot meet this deadline, please inform us at rjsproduction@springernature.com immediately.

You will not receive your proofs until the publishing agreement has been received through our system.

Due to the importance of these deadlines, we ask that you please let us know now whether you will be difficult to contact over the next month. If this is the case, we ask you provide us with the contact information (email, phone and fax) of someone who will be able to check the proofs on your behalf, and who will be available to address any last-minute problems.

Acceptance is conditional on the data in the manuscript not being published elsewhere, or announced in the print or electronic media, until the embargo/publication date. These restrictions are not intended to deter you from presenting your data at academic meetings and conferences, but any enquiries from the media about papers not yet scheduled for publication should be referred to us.

Please note that <i>Nature Immunology</i> is a Transformative Journal (TJ). Authors may publish their research with us through the traditional subscription access route or make their paper immediately open access through payment of an article-processing charge (APC). Authors will not be required to make a final decision about access to their article until it has been accepted. Find out more about Transformative Journals.

Authors may need to take specific actions to achieve compliance with funder and institutional open access mandates. If your research is supported by a funder that requires immediate open access (e.g. according to Plan S principles) then you should select the gold OA route, and we will direct you to the compliant route where possible. For authors selecting the subscription publication route, the journal's standard licensing terms will need to be accepted, including self-archiving policies. Those licensing terms will supersede any other terms that the author or any third party may assert apply to any version of the manuscript.

If you have any questions about our publishing options, costs, Open Access requirements, or our legal forms, please contact ASJournals@springernature.com

Your paper will be published online soon after we receive your corrections and will appear in print in the next available issue. Content is published online weekly on Mondays and Thursdays, and the embargo is set at 16:00 London time (GMT)/11:00 am US Eastern time (EST) on the day of publication. Now is the time to inform your Public Relations or Press Office about your paper, as they might be interested in promoting its publication. This will allow them time to prepare an accurate and satisfactory press release. Include your manuscript tracking number (NI-A35702B) and the name of the journal, which they will need when they contact our office.

About one week before your paper is published online, we shall be distributing a press release to news organizations worldwide, which may very well include details of your work. We are happy for your institution or funding agency to prepare its own press release, but it must mention the embargo date and Nature Immunology. Our Press Office will contact you closer to the time of publication, but if you or your Press Office have any enquiries in the meantime, please contact press@nature.com.

Also, if you have any spectacular or outstanding figures or graphics associated with your manuscript - though not necessarily included with your submission - we'd be delighted to consider them as candidates for our cover. Simply send an electronic version (accompanied by a hard copy) to us with a possible cover caption enclosed.

To assist our authors in disseminating their research to the broader community, our SharedIt initiative provides you with a unique shareable link that will allow anyone (with or without a subscription) to read the published article. Recipients of the link with a subscription will also be able to download and print the PDF.

As soon as your article is published, you will receive an automated email with your shareable link.

You can now use a single sign-on for all your accounts, view the status of all your manuscript submissions and reviews, access usage statistics for your published articles and download a record of your refereeing activity for the Nature journals.

If you have not already done so, we strongly recommend that you upload the step-by-step protocols used in this manuscript to the Protocol Exchange. Protocol Exchange is an open online resource that allows researchers to share their detailed experimental know-how. All uploaded protocols are made freely available, assigned DOIs for ease of citation and fully searchable through nature.com. Protocols can be linked to any publications in which they are used and will be linked to from your article. You can also establish a dedicated page to collect all your lab Protocols. By uploading your Protocols to Protocol Exchange, you are enabling researchers to more readily reproduce or adapt the methodology you use, as well as increasing the visibility of your protocols and papers. Upload your Protocols at www.nature.com/protocolexchange/. Further information can be found at www.nature.com/protocolexchange/about .

Please note that we encourage the authors to self-archive their manuscript (the accepted version before copy editing) in their institutional repository, and in their funders' archives, six months after publication. Nature Portfolio recognizes the efforts of funding bodies to increase access of the research they fund, and strongly encourages authors to participate in such efforts. For information about our editorial policy, including license agreement and author copyright, please visit www.nature.com/ni/about/ed_policies/index.html

An online order form for reprints of your paper is available at https://www.nature.com/reprints/author-reprints.html. Please let your coauthors and your institutions' public affairs office know that they are also welcome to order reprints by this method.

Kind regards,

natureresearch

Laurie

Laurie A. Dempsey, Ph.D.
Senior Editor
Nature Immunology
l.dempsey@us.nature.com
ORCID: 0000-0002-3304-796X
